# Supplementary material for: Visualization of multidrug-resistant bacterial infection trends in the intensive care units
Source: PLoS One. 2025 Aug 28;20(8):e0330765. doi: 10.1371/journal.pone.0330765 (PMC12393710; doi:10.1371/journal.pone.0330765)
Supplement: S1 File — (DOCX) [file pone.0330765.s001.docx]

**Supplementary materials 1**


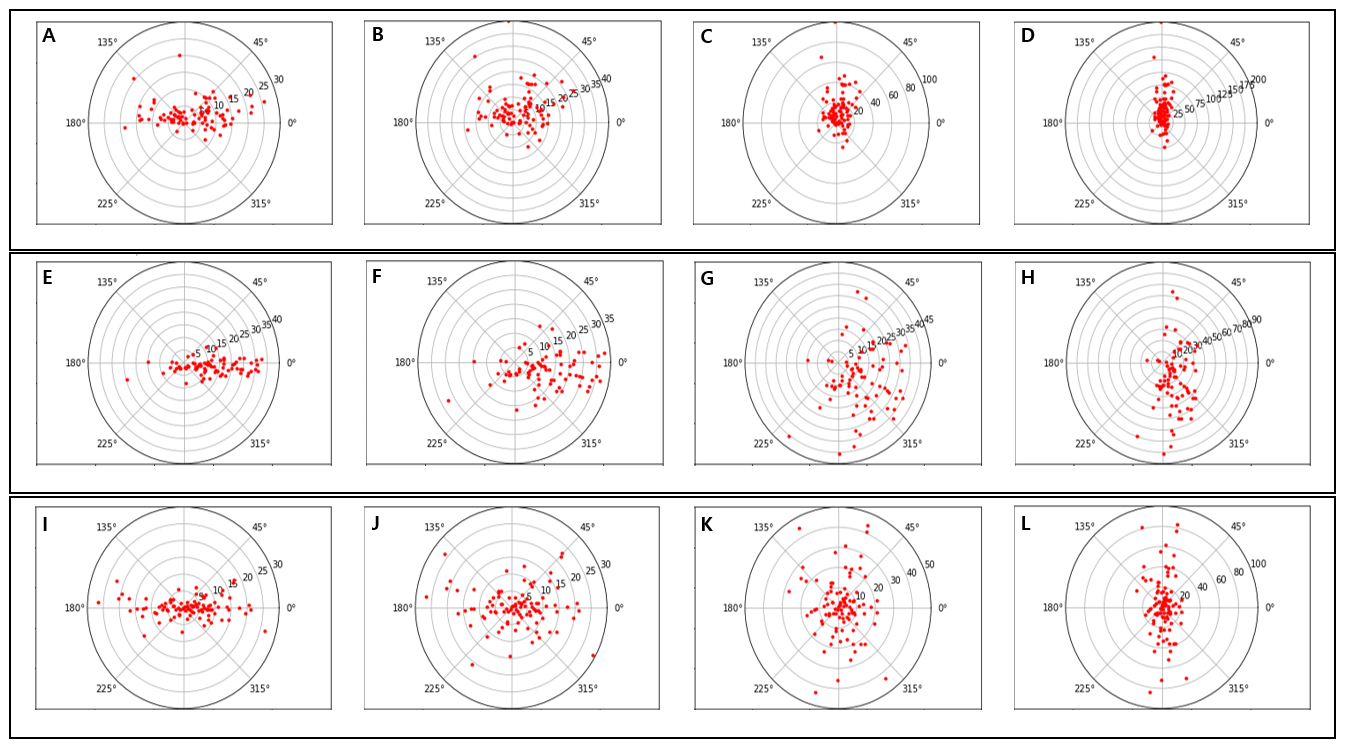


**Figure S1. Data distribution tendency according to scale-up factor**

**A**. Randomized data group 1 with scale-up magnitude 10. **B**. Randomized data group 1 with scale-up magnitude 20. **C**. Randomized data group 1 with scale-up magnitude 50. **D**. Randomized data group 1 with scale-up magnitude 100. **E**. Randomized data group 2 with scale-up magnitude 10. **F**. Randomized data group 2 with scale-up magnitude 20. **G**. Randomized data group 2 with scale-up magnitude 50. **H**. Randomized data group 2 with scale-up magnitude 100. **I**. Randomized data group 3 with scale-up magnitude 10. **J**. Randomized data group 3 with scale-up magnitude 20. **K**. Randomized data group 3 with scale-up magnitude 50. **L**. Randomized data group 3 with scale-up magnitude 100.
