# Supplementary material for: Visualization of multidrug-resistant bacterial infection trends in the intensive care units
Source: PLoS One. 2025 Aug 28;20(8):e0330765. doi: 10.1371/journal.pone.0330765 (PMC12393710; doi:10.1371/journal.pone.0330765)
Supplement: S2 File — (DOCX) [file pone.0330765.s002.docx]

**Supplementary materials 2**

**Vector calculation formulas**

Step 1. At the ICU level, we categorized a single pathogen based on its carbapenem susceptibility and calculated the overall rate of infections (pneumonia, UTI, and BSI) by time period (year, quarter). The formula used for these calculations is provided below.

Overall infection rate = number of infections (pneumonia, UTI, or BSI) / total patient–days × 1000

Step 2. The central distribution points for each of the carbapenem-susceptible and carbapenem-resistant groups were calculated. The formula used for these calculations is as follows.

x : time (year, quarter)

(The 5-year observation period was divided into a total of 20 quarters, which were sequentially matched in order from 1 to 20.)

average of x = (sum of existed x) / (number of existed x)

y : overall infection rate

average of y = (sum of existed y) / (number of existed y)

Step 3. The length and angle of the vector were calculated using the formula provided below. (In this paper, a scale-up factor of 20 was selected.)

Length of vector =sqrt[(carbapenem-resistant group’s average of x – carbapenem-susceptible group’s average of x)^2^+{(carbapenem-resistant group’s average of y * scale-up factor) – (carbapenem-susceptible group’s average of y * scale-up factor)}^2^]

Angle of vector = (360/2π) * arctan[{(carbapenem-resistant group’s average of y * scale-up factor) – (carbapenem-susceptible group’s average of y * scale-up factor)}/(carbapenem-resistant group’s average of x – carbapenem-susceptible group’s average of x)]
