# Supplementary material for: Visualization of multidrug-resistant bacterial infection trends in the intensive care units
Source: PLoS One. 2025 Aug 28;20(8):e0330765. doi: 10.1371/journal.pone.0330765 (PMC12393710; doi:10.1371/journal.pone.0330765)
Supplement: S3 File — (DOCX) [file pone.0330765.s003.docx]

**Supplementary materials 3**

**Python script for data analysis**

(1) Individual ICU : Overall infection rate & quarter graph

Repeat the same method for pathogens (AB, KP, and PA).

Repeat the same method for the first 5 years and the last 5 years.

import pandas as pd

import matplotlib.pyplot as plt

icu_data_s_path = '/ICU_data_S.xlsx'

icu_data_r_path = '/ICU_data_R.xlsx'

icu_data_s = pd.read_excel(icu_data_s_path)

icu_data_r = pd.read_excel(icu_data_r_path)

merged_data = pd.merge(icu_data_s, icu_data_r, on=['ICU_code', 'Quarter'],

suffixes=('_CS', '_CR'))

icu_codes = merged_data['ICU_code'].unique()

for icu_code in icu_codes:

subset = merged_data[merged_data['ICU_code'] == icu_code]

plt.figure(figsize=(10, 6))

plt.scatter(subset['Quarter'], subset['Overall_infection_rate_CS'], marker='x')

plt.scatter(subset['Quarter'], subset['Overall_infection_rate_CR'], marker='o')

plt.xlabel('Time (quarter)')

plt.ylabel('Overall infection rate')

plt.xlim(0, 20)

plt.ylim(0, 10)

plt.legend(['CS', 'CR'])

plt.title(f'Overall Infection Rate by Quarter for ICU Code {icu_code}')

plt.show()

(2) Individual ICU : Distribution center with error bar graph

Repeat the same method for pathogens (AB, KP, and PA).

Repeat the same method for the first 5 years and the last 5 years.

Please change the column name [Overall_infection_rate_CS] and

[Overall_infection_rate_CR] to [Overall_infection_rate] in both ‘ICU_data_S’

and ‘ICU_data_R’ files, and then run the code below."

import pandas as pd

import numpy as np

from scipy import stats

import matplotlib.pyplot as plt

file_path_S = 'ICU_data_S.xlsx'

file_path_R = 'ICU_data_R.xlsx'

data_S = pd.read_excel(file_path_S)

data_R = pd.read_excel(file_path_R)

data_S = data_S.dropna(subset=['Quarter', 'Overall_infection_rate'])

data_R = data_R.dropna(subset=['Quarter', 'Overall_infection_rate'])

def calculate_statistics(data, icu_code):

group = data[data['ICU_code'] == icu_code]

x_mean = group['Quarter'].mean()

y_mean = group['Overall_infection_rate'].mean()

count = group['Overall_infection_rate'].count()

error_bar = stats.sem(group['Overall_infection_rate']) * stats.t.ppf((1 + 0.95) / 2.,

count - 1)

return x_mean, y_mean, count, error_bar

icu_codes_S = data_S['ICU_code'].unique()

icu_codes_R = data_R['ICU_code'].unique()

results = []

icu_codes = set(icu_codes_S).union(set(icu_codes_R))

for icu_code in icu_codes:

x_S, y_S, error_S = None, None, None

x_R, y_R, error_R = None, None, None

if icu_code in icu_codes_S:

x_S, y_S, _, error_S = calculate_statistics(data_S, icu_code)

if icu_code in icu_codes_R:

x_R, y_R, _, error_R = calculate_statistics(data_R, icu_code)

results.append([icu_code, x_S, y_S, error_S, x_R, y_R, error_R])

results_df = pd.DataFrame(results, columns=['ICU_code', 'x_S', 'y_S', 'error_S', 'x_R', 'y_R',

'error_R'])

output_path = '/ICU_code_statistics.xlsx'

results_df.to_excel(output_path, index=False)

import pandas as pd

import numpy as np

from scipy import stats

import matplotlib.pyplot as plt

file_path = '/ICU_code_statistics.xlsx'

df = pd.read_excel(file_path)

icu_codes = df['ICU_code'].unique()

for icu_code in icu_codes:

subset = df[df['ICU_code'] == icu_code]

plt.figure(figsize=(10, 6))

plt.errorbar(subset['x_S'], subset['y_S'], yerr=subset['error_S'], fmt='x',

label='Average of CS')

plt.errorbar(subset['x_R'], subset['y_R'], yerr=subset['error_R'], fmt='o',

label='Average of CR')

plt.title(f'ICU Code: {icu_code}')

plt.xlabel('Time (quarter)')

plt.ylabel('Overall infection rate')

plt.xlim(0, 20)

plt.ylim(0, 10)

plt.legend()

plt.savefig(f'{icu_code}_infection_rate_plot.png')

plt.show()

(3) Individual ICU : Vectorization graph

Repeat the same method for pathogens (AB, KP, and PA).

Repeat the same method for the first 5 years and the last 5 years.

import pandas as pd

import numpy as np

import matplotlib.pyplot as plt

file_path = '/ICU_code_statistics.xlsx'

df = pd.read_excel(file_path)

scale_up_factor = 20

df['y_S_scale'] = df['y_S'] * scale_up_factor

df['y_R_scale'] = df['y_R'] * scale_up_factor

df['length'] = np.sqrt((df['x_R'] - df['x_S'])**2 + (df['y_R_scale'] - df['y_S_scale'])**2)

df['angle'] = (360 / (2 * np.pi)) * np.arctan((df['y_R_scale'] - df['y_S_scale']) /

(df['x_R'] - df['x_S']))

df.loc[df['x_R'] - df['x_S'] < 0, 'angle'] += 180

mean_length = df['length'].mean()

df['mean_length'] = mean_length

output_file_path = 'AB/Pre/results_df_vector.xlsx'

df.to_excel(output_file_path, index=False)

import pandas as pd

import matplotlib.pyplot as plt

import numpy as np

file_path = '/results_df_vector.xlsx'

df = pd.read_excel(file_path)

def plot_vector_with_dot(row):

length = row['length']

angle = np.deg2rad(row['angle'])

end_x = length * np.cos(angle)

end_y = length * np.sin(angle)

fig, ax = plt.subplots(subplot_kw={'projection': 'polar'})

ax.set_ylim(0, 80)

ax.annotate('', xy=(angle, length), xytext=(0, 0),

arrowprops=dict(facecolor='red', shrink=0.05, width=0.5, headwidth=8))

ax.plot(angle, length, 'ro')

ax.set_title(f"ICU_code: {row['ICU_code']}")

plt.show()

for index, row in df.iterrows():

plot_vector_with_dot(row)

(4) Total ICU: End points of vectors graph

Repeat the same method for pathogens (AB, KP, and PA).

Repeat the same method for the first 5 years and the last 5 years.

import numpy as np

import pandas as pd

import matplotlib.pyplot as plt

import matplotlib.patches as patches

file_path = '/results_df_vector.xlsx'

df = pd.read_excel(file_path)

Sx = df['x_S']

Sy = df['y_S']

Rx = df['x_R']

Ry = df['y_R']

V_length = df['length']

V_angle = df['angle']

plt.title("Total ICU vectorization graph")

plt.axes(polar=True)

plt.plot(V_angle, V_length, "r.")

plt.ylim(0, 80)

plt.show()

(5) Category classification

Repeat the same method for pathogens (AB, KP, and PA).

Repeat the same method for the first 5 years and the last 5 years.

* Cutoff_length calculation with Excel :

cutoff_legnth **=** [(mean_length_AB_first * count_AB_first + mean_legnth_KP_first * count_KP_first + mean_length_PA_first * count_PA_first) + (mean_length_AB_last * count_AB_last + mean_legnth_KP_last * count_KP_last + mean_length_PA_last * count_PA_last)] / [(count_AB_first + count_KP_first + count_PA_first) + (count_AB_last + count_KP_last + count_PA_last)]

import pandas as pd

file_path = '/results_df_vector.xlsx'

df = pd.read_excel(file_path)

total_ICU_count = df['ICU_code'].count()

cutoff_length = 29.32 # number calculated with Excel

red_category = df[(df['angle'] >= 0) & (df['angle'] <= 90) &

df['length'] < cutoff_length)].shape[0]

yellow_category = df[((df['angle'] >= 270) & (df['angle'] <= 360) | (df['angle'] >= -90) &

(df['angle'] < 0)) & (df['length'] > cutoff_length)].shape[0]

green_category = df[(df['angle'] > 90) & (df['angle'] < 270)].shape[0]

results = {

'Total_ICU_Count': [total_ICU_count],

'Red_Category_Count': [red_category],

'Yellow_Category_Count': [yellow_category],

'Green_Category_Count': [green_category]

}

results_df = pd.DataFrame(results)

output_file_path = '/ICU_category_counts.xlsx'

results_df.to_excel(output_file_path, index=False)

output_file_path
