# Supplementary figures and images for: Visualization of multidrug-resistant bacterial infection trends in the intensive care units (part 2 of 2)
Source: PLoS One. 2025 Aug 28;20(8):e0330765. doi: 10.1371/journal.pone.0330765 (PMC12393710; doi:10.1371/journal.pone.0330765)

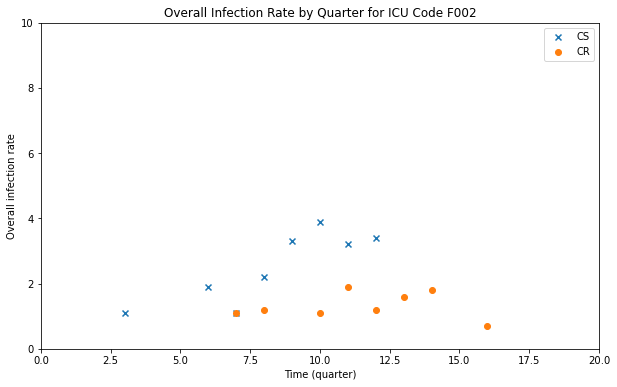

Supplement: S4 File — (ZIP) [file pone.0330765.s004.zip › Synthetic dataset/Result/KP/Post/Figure1-1/Figure 2025-01-08 223117 (16).png]

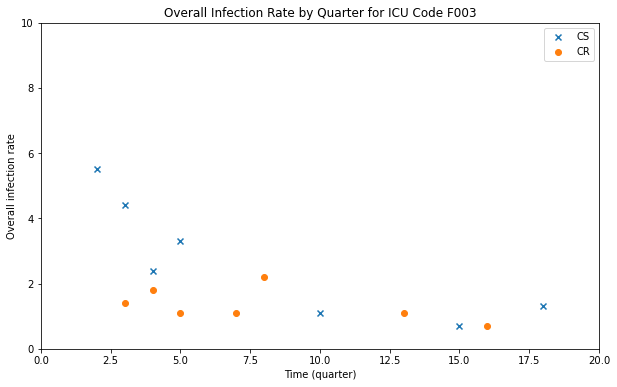

Supplement: S4 File — (ZIP) [file pone.0330765.s004.zip › Synthetic dataset/Result/KP/Post/Figure1-1/Figure 2025-01-08 223117 (17).png]

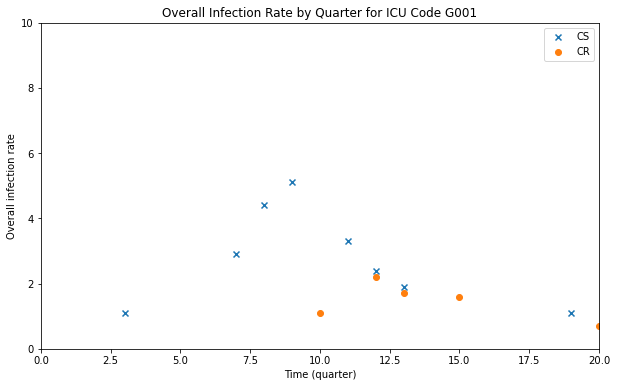

Supplement: S4 File — (ZIP) [file pone.0330765.s004.zip › Synthetic dataset/Result/KP/Post/Figure1-1/Figure 2025-01-08 223117 (18).png]

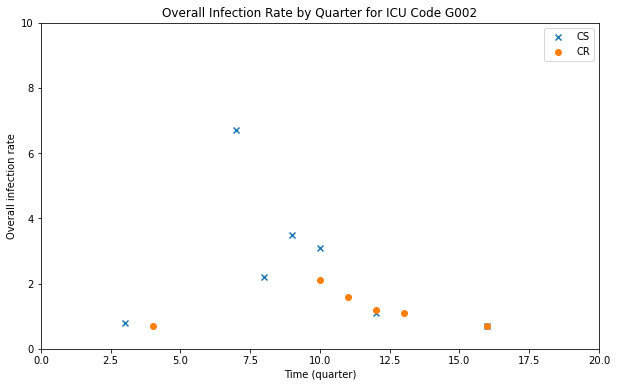

Supplement: S4 File — (ZIP) [file pone.0330765.s004.zip › Synthetic dataset/Result/KP/Post/Figure1-1/Figure 2025-01-08 223117 (19).png]

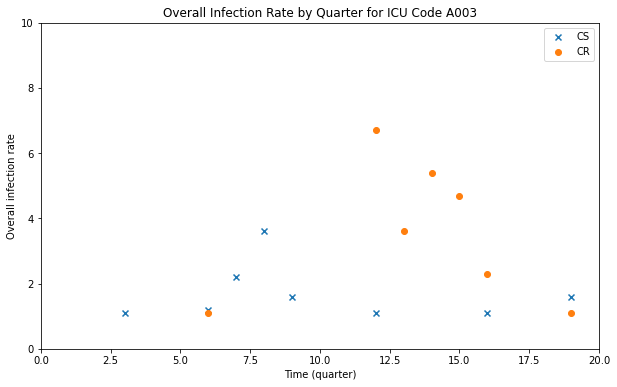

Supplement: S4 File — (ZIP) [file pone.0330765.s004.zip › Synthetic dataset/Result/KP/Post/Figure1-1/Figure 2025-01-08 223117 (2).png]

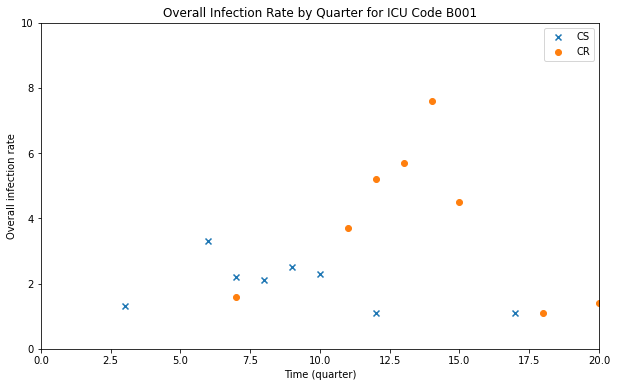

Supplement: S4 File — (ZIP) [file pone.0330765.s004.zip › Synthetic dataset/Result/KP/Post/Figure1-1/Figure 2025-01-08 223117 (3).png]

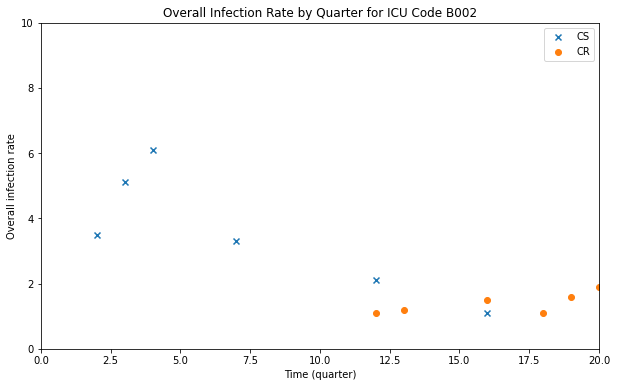

Supplement: S4 File — (ZIP) [file pone.0330765.s004.zip › Synthetic dataset/Result/KP/Post/Figure1-1/Figure 2025-01-08 223117 (4).png]

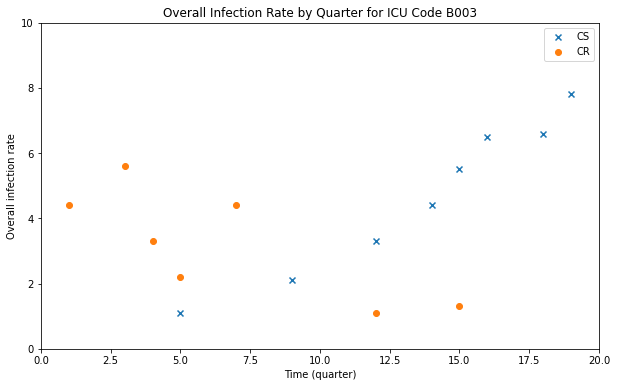

Supplement: S4 File — (ZIP) [file pone.0330765.s004.zip › Synthetic dataset/Result/KP/Post/Figure1-1/Figure 2025-01-08 223117 (5).png]

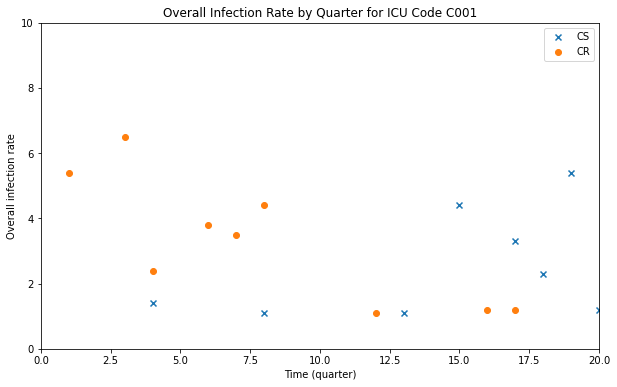

Supplement: S4 File — (ZIP) [file pone.0330765.s004.zip › Synthetic dataset/Result/KP/Post/Figure1-1/Figure 2025-01-08 223117 (6).png]

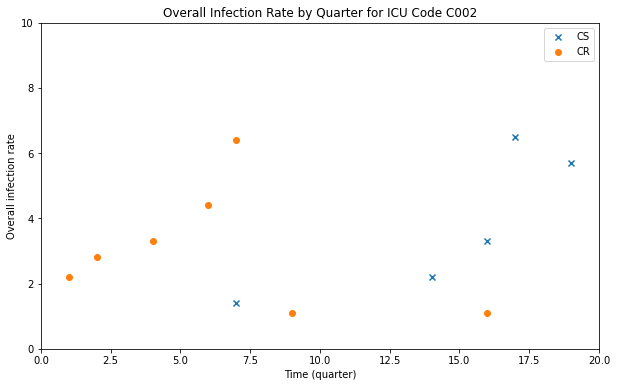

Supplement: S4 File — (ZIP) [file pone.0330765.s004.zip › Synthetic dataset/Result/KP/Post/Figure1-1/Figure 2025-01-08 223117 (7).png]

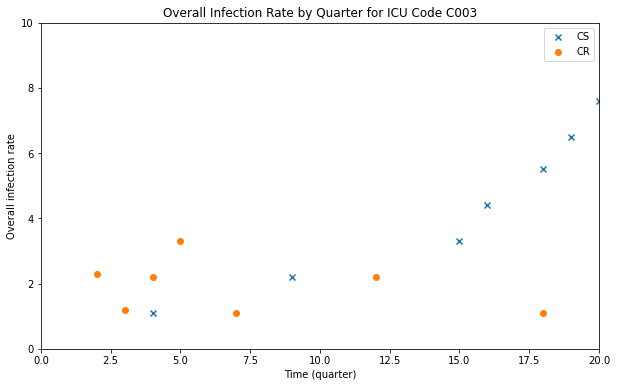

Supplement: S4 File — (ZIP) [file pone.0330765.s004.zip › Synthetic dataset/Result/KP/Post/Figure1-1/Figure 2025-01-08 223117 (8).png]

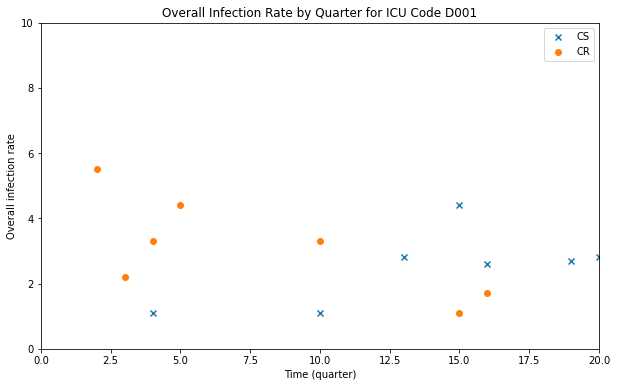

Supplement: S4 File — (ZIP) [file pone.0330765.s004.zip › Synthetic dataset/Result/KP/Post/Figure1-1/Figure 2025-01-08 223117 (9).png]

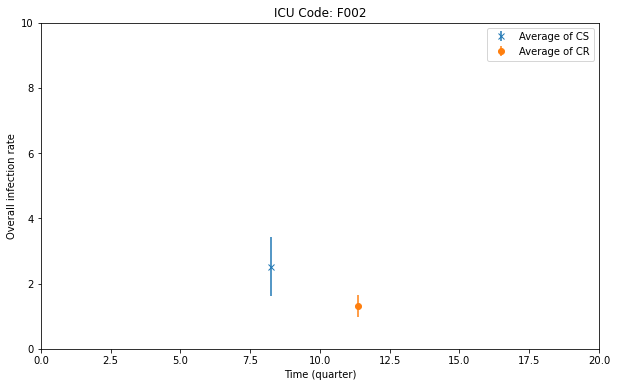

Supplement: S4 File — (ZIP) [file pone.0330765.s004.zip › Synthetic dataset/Result/KP/Post/Figure1-2/Figure 2025-01-11 093409 (0).png]

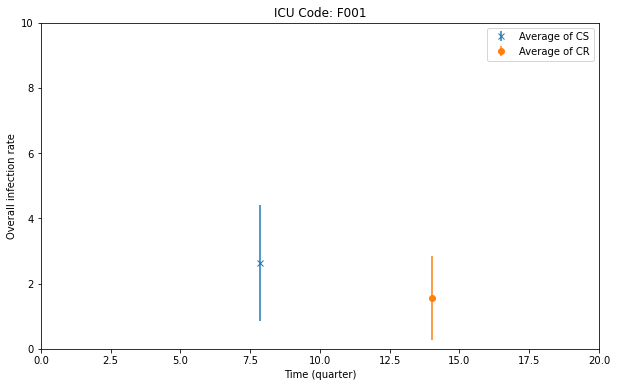

Supplement: S4 File — (ZIP) [file pone.0330765.s004.zip › Synthetic dataset/Result/KP/Post/Figure1-2/Figure 2025-01-11 093409 (1).png]

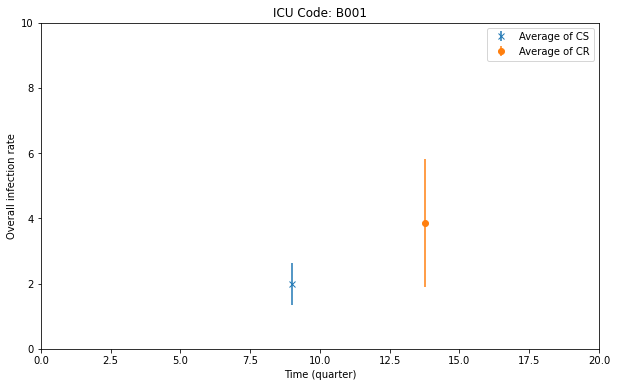

Supplement: S4 File — (ZIP) [file pone.0330765.s004.zip › Synthetic dataset/Result/KP/Post/Figure1-2/Figure 2025-01-11 093409 (10).png]

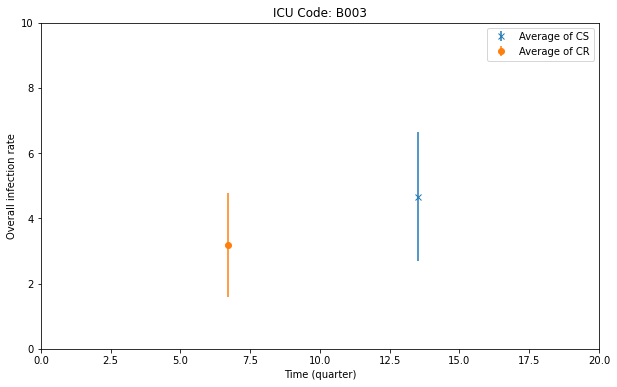

Supplement: S4 File — (ZIP) [file pone.0330765.s004.zip › Synthetic dataset/Result/KP/Post/Figure1-2/Figure 2025-01-11 093409 (11).png]

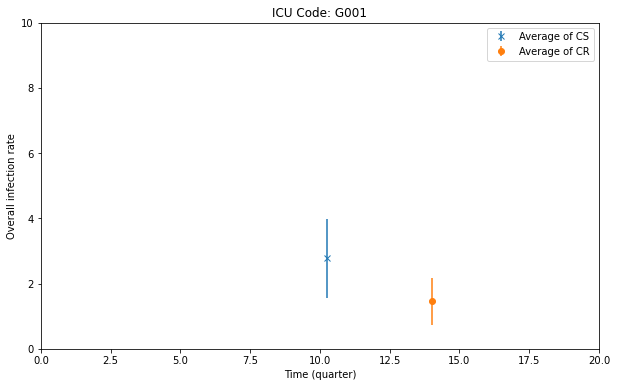

Supplement: S4 File — (ZIP) [file pone.0330765.s004.zip › Synthetic dataset/Result/KP/Post/Figure1-2/Figure 2025-01-11 093409 (12).png]

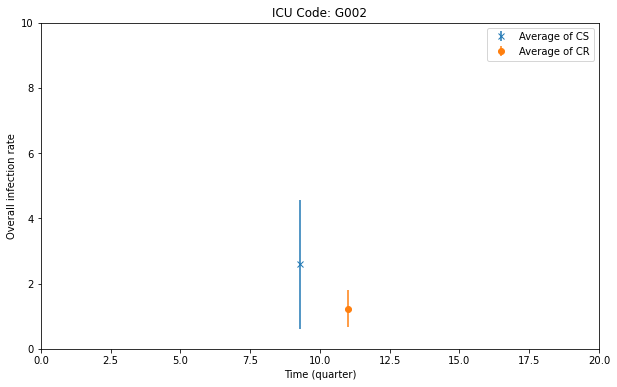

Supplement: S4 File — (ZIP) [file pone.0330765.s004.zip › Synthetic dataset/Result/KP/Post/Figure1-2/Figure 2025-01-11 093409 (13).png]

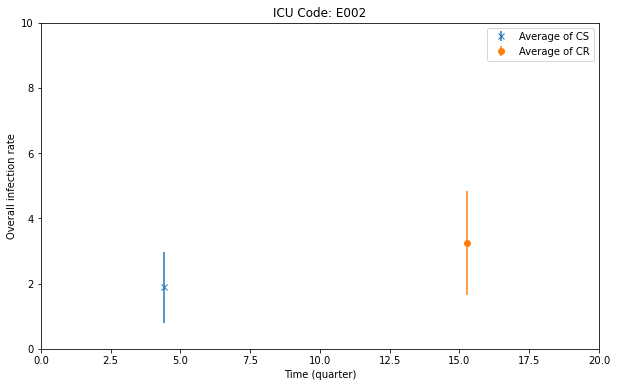

Supplement: S4 File — (ZIP) [file pone.0330765.s004.zip › Synthetic dataset/Result/KP/Post/Figure1-2/Figure 2025-01-11 093409 (14).png]

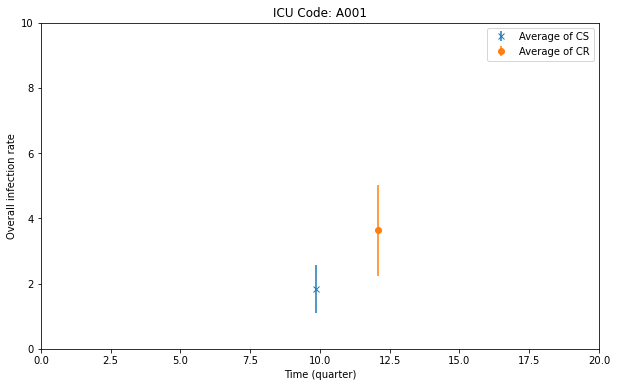

Supplement: S4 File — (ZIP) [file pone.0330765.s004.zip › Synthetic dataset/Result/KP/Post/Figure1-2/Figure 2025-01-11 093409 (15).png]

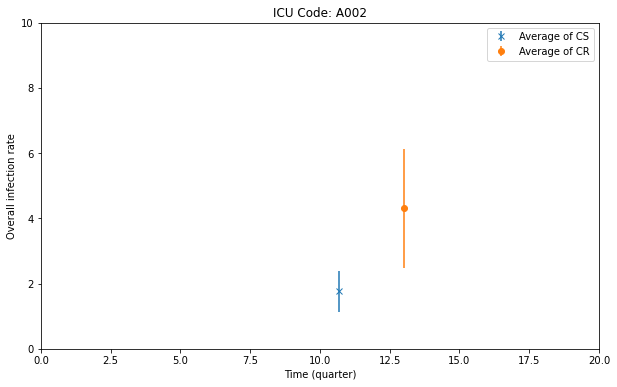

Supplement: S4 File — (ZIP) [file pone.0330765.s004.zip › Synthetic dataset/Result/KP/Post/Figure1-2/Figure 2025-01-11 093409 (16).png]

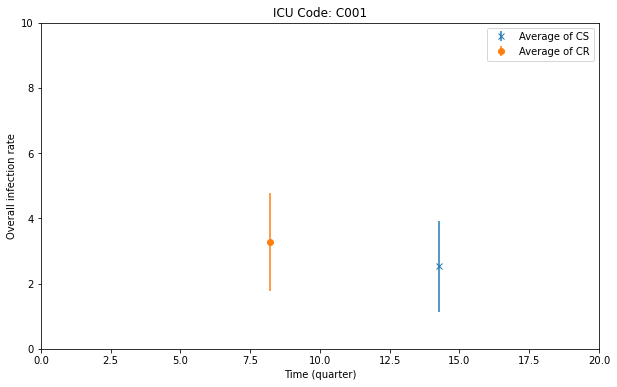

Supplement: S4 File — (ZIP) [file pone.0330765.s004.zip › Synthetic dataset/Result/KP/Post/Figure1-2/Figure 2025-01-11 093409 (17).png]

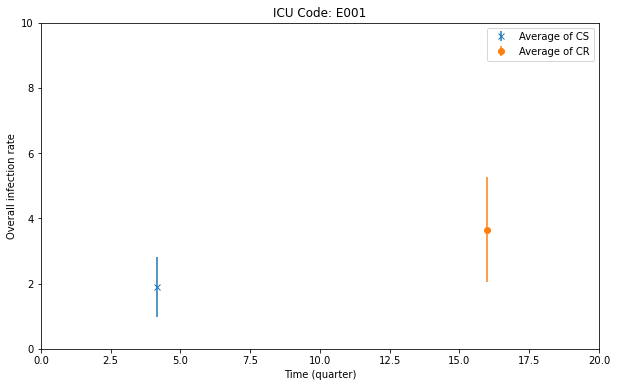

Supplement: S4 File — (ZIP) [file pone.0330765.s004.zip › Synthetic dataset/Result/KP/Post/Figure1-2/Figure 2025-01-11 093409 (18).png]

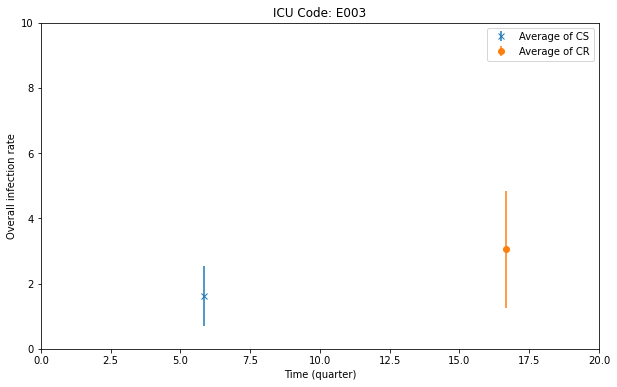

Supplement: S4 File — (ZIP) [file pone.0330765.s004.zip › Synthetic dataset/Result/KP/Post/Figure1-2/Figure 2025-01-11 093409 (19).png]

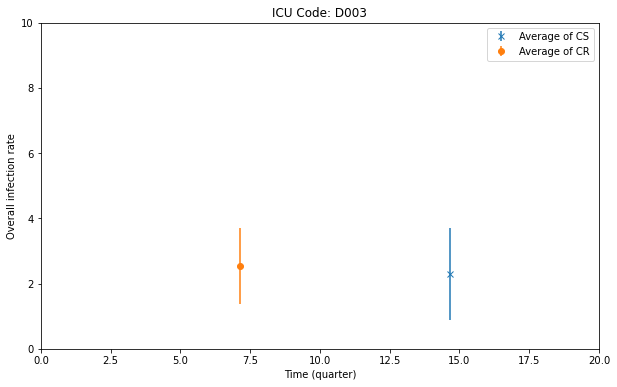

Supplement: S4 File — (ZIP) [file pone.0330765.s004.zip › Synthetic dataset/Result/KP/Post/Figure1-2/Figure 2025-01-11 093409 (2).png]

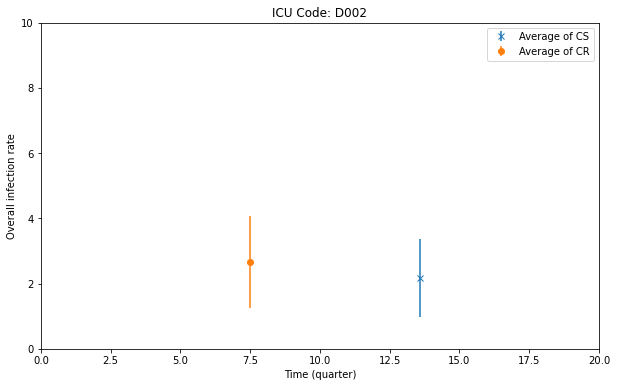

Supplement: S4 File — (ZIP) [file pone.0330765.s004.zip › Synthetic dataset/Result/KP/Post/Figure1-2/Figure 2025-01-11 093409 (3).png]

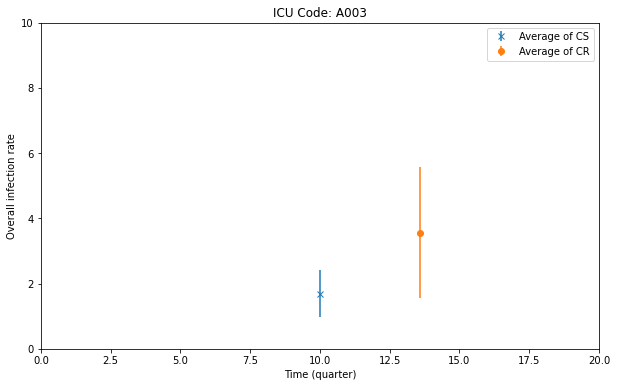

Supplement: S4 File — (ZIP) [file pone.0330765.s004.zip › Synthetic dataset/Result/KP/Post/Figure1-2/Figure 2025-01-11 093409 (4).png]

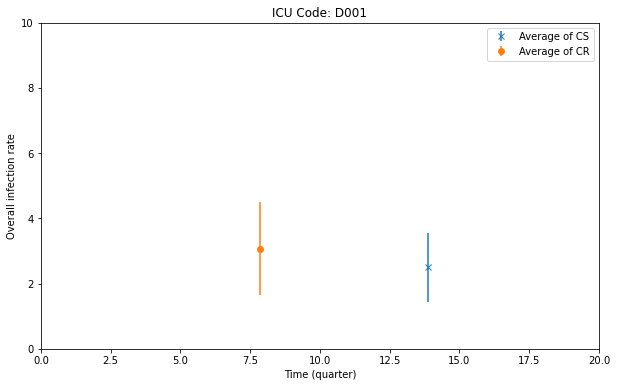

Supplement: S4 File — (ZIP) [file pone.0330765.s004.zip › Synthetic dataset/Result/KP/Post/Figure1-2/Figure 2025-01-11 093409 (5).png]

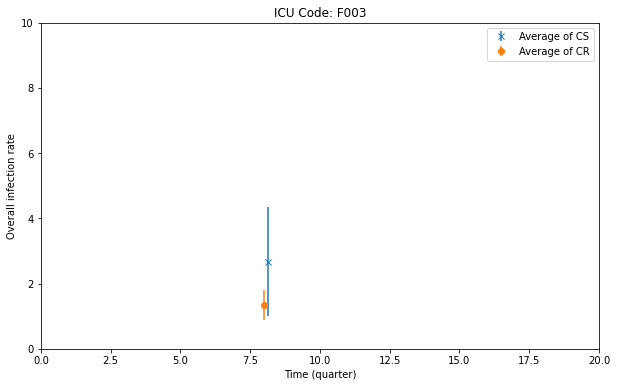

Supplement: S4 File — (ZIP) [file pone.0330765.s004.zip › Synthetic dataset/Result/KP/Post/Figure1-2/Figure 2025-01-11 093409 (6).png]

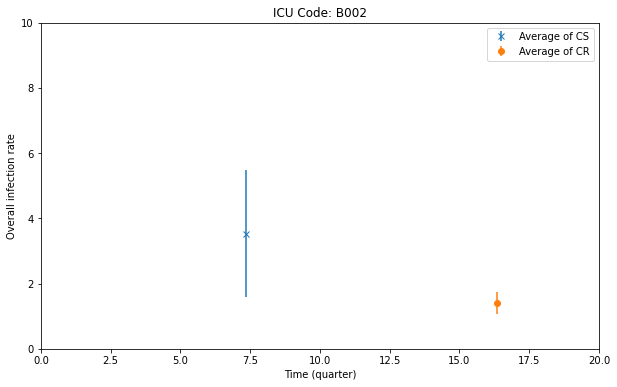

Supplement: S4 File — (ZIP) [file pone.0330765.s004.zip › Synthetic dataset/Result/KP/Post/Figure1-2/Figure 2025-01-11 093409 (7).png]

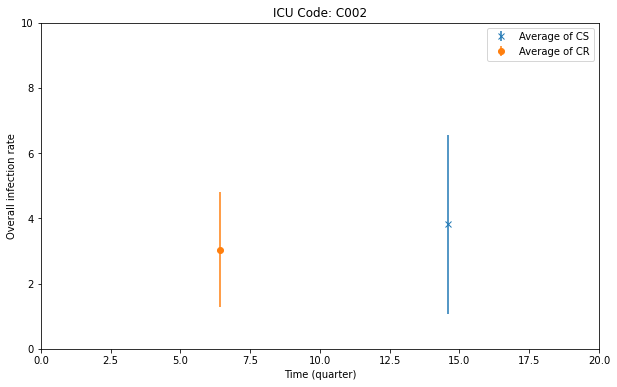

Supplement: S4 File — (ZIP) [file pone.0330765.s004.zip › Synthetic dataset/Result/KP/Post/Figure1-2/Figure 2025-01-11 093409 (8).png]

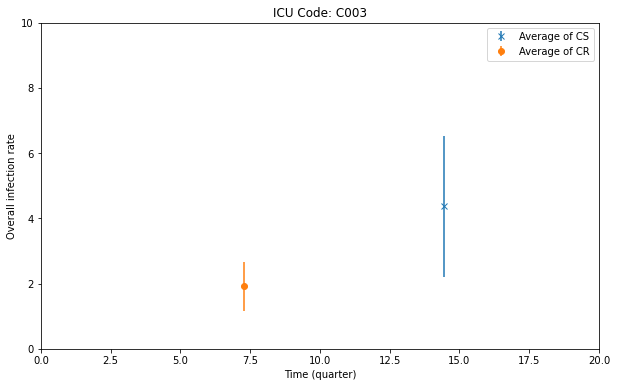

Supplement: S4 File — (ZIP) [file pone.0330765.s004.zip › Synthetic dataset/Result/KP/Post/Figure1-2/Figure 2025-01-11 093409 (9).png]

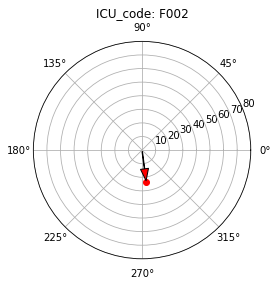

Supplement: S4 File — (ZIP) [file pone.0330765.s004.zip › Synthetic dataset/Result/KP/Post/Figure1-3/Figure 2025-01-11 104158 (0).png]

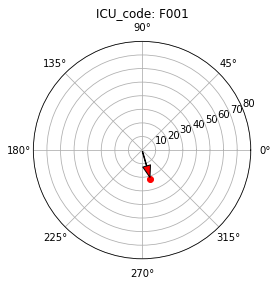

Supplement: S4 File — (ZIP) [file pone.0330765.s004.zip › Synthetic dataset/Result/KP/Post/Figure1-3/Figure 2025-01-11 104158 (1).png]

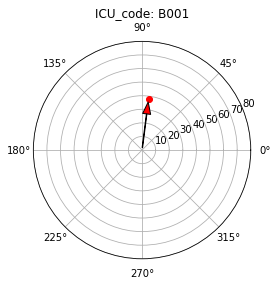

Supplement: S4 File — (ZIP) [file pone.0330765.s004.zip › Synthetic dataset/Result/KP/Post/Figure1-3/Figure 2025-01-11 104158 (10).png]

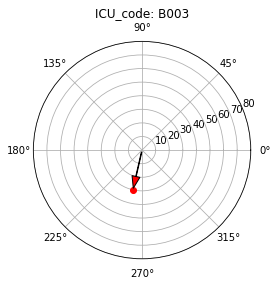

Supplement: S4 File — (ZIP) [file pone.0330765.s004.zip › Synthetic dataset/Result/KP/Post/Figure1-3/Figure 2025-01-11 104158 (11).png]

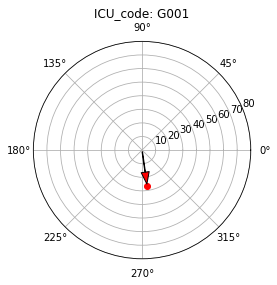

Supplement: S4 File — (ZIP) [file pone.0330765.s004.zip › Synthetic dataset/Result/KP/Post/Figure1-3/Figure 2025-01-11 104158 (12).png]

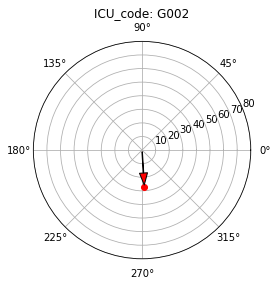

Supplement: S4 File — (ZIP) [file pone.0330765.s004.zip › Synthetic dataset/Result/KP/Post/Figure1-3/Figure 2025-01-11 104158 (13).png]

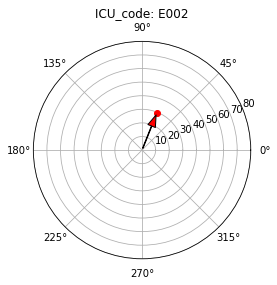

Supplement: S4 File — (ZIP) [file pone.0330765.s004.zip › Synthetic dataset/Result/KP/Post/Figure1-3/Figure 2025-01-11 104158 (14).png]

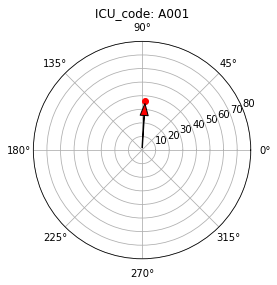

Supplement: S4 File — (ZIP) [file pone.0330765.s004.zip › Synthetic dataset/Result/KP/Post/Figure1-3/Figure 2025-01-11 104158 (15).png]

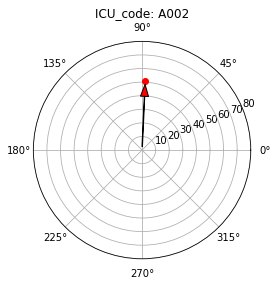

Supplement: S4 File — (ZIP) [file pone.0330765.s004.zip › Synthetic dataset/Result/KP/Post/Figure1-3/Figure 2025-01-11 104158 (16).png]

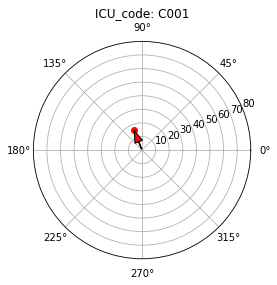

Supplement: S4 File — (ZIP) [file pone.0330765.s004.zip › Synthetic dataset/Result/KP/Post/Figure1-3/Figure 2025-01-11 104158 (17).png]

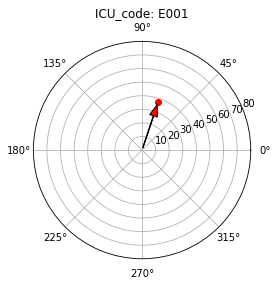

Supplement: S4 File — (ZIP) [file pone.0330765.s004.zip › Synthetic dataset/Result/KP/Post/Figure1-3/Figure 2025-01-11 104158 (18).png]

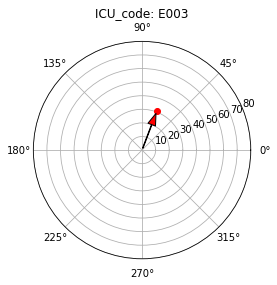

Supplement: S4 File — (ZIP) [file pone.0330765.s004.zip › Synthetic dataset/Result/KP/Post/Figure1-3/Figure 2025-01-11 104158 (19).png]

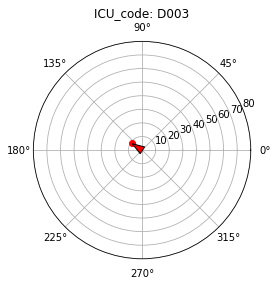

Supplement: S4 File — (ZIP) [file pone.0330765.s004.zip › Synthetic dataset/Result/KP/Post/Figure1-3/Figure 2025-01-11 104158 (2).png]

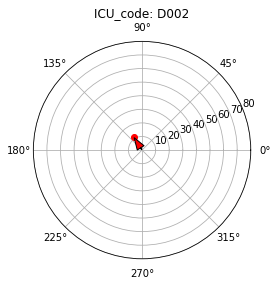

Supplement: S4 File — (ZIP) [file pone.0330765.s004.zip › Synthetic dataset/Result/KP/Post/Figure1-3/Figure 2025-01-11 104158 (3).png]

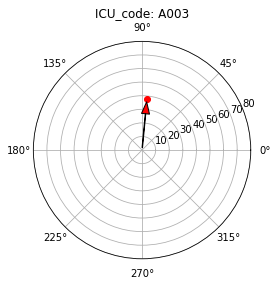

Supplement: S4 File — (ZIP) [file pone.0330765.s004.zip › Synthetic dataset/Result/KP/Post/Figure1-3/Figure 2025-01-11 104158 (4).png]

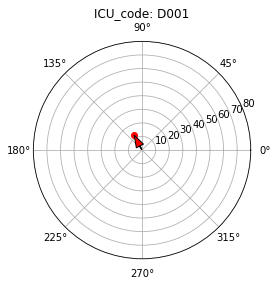

Supplement: S4 File — (ZIP) [file pone.0330765.s004.zip › Synthetic dataset/Result/KP/Post/Figure1-3/Figure 2025-01-11 104158 (5).png]

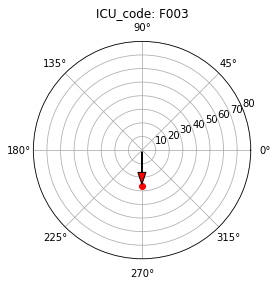

Supplement: S4 File — (ZIP) [file pone.0330765.s004.zip › Synthetic dataset/Result/KP/Post/Figure1-3/Figure 2025-01-11 104158 (6).png]

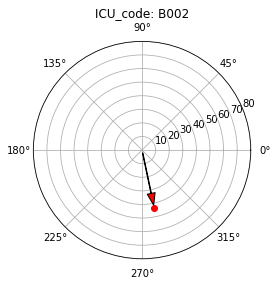

Supplement: S4 File — (ZIP) [file pone.0330765.s004.zip › Synthetic dataset/Result/KP/Post/Figure1-3/Figure 2025-01-11 104158 (7).png]

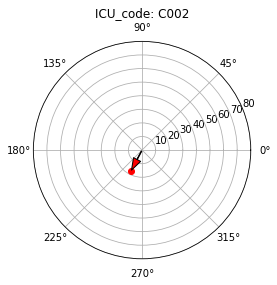

Supplement: S4 File — (ZIP) [file pone.0330765.s004.zip › Synthetic dataset/Result/KP/Post/Figure1-3/Figure 2025-01-11 104158 (8).png]

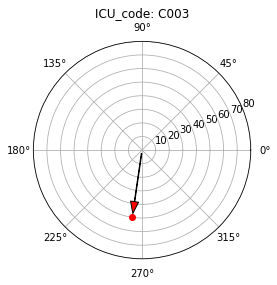

Supplement: S4 File — (ZIP) [file pone.0330765.s004.zip › Synthetic dataset/Result/KP/Post/Figure1-3/Figure 2025-01-11 104158 (9).png]

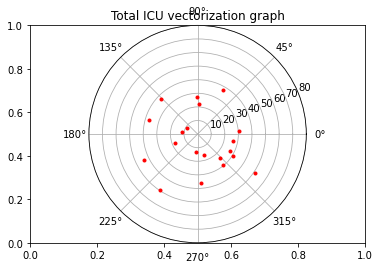

Supplement: S4 File — (ZIP) [file pone.0330765.s004.zip › Synthetic dataset/Result/KP/Post/Figure4/Figure 2025-01-11 104829 (0).png]

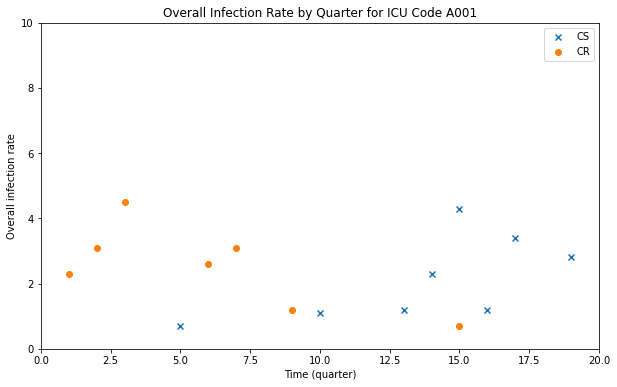

Supplement: S4 File — (ZIP) [file pone.0330765.s004.zip › Synthetic dataset/Result/KP/Pre/Figure1-1/Figure 2025-01-08 220830 (0).png]

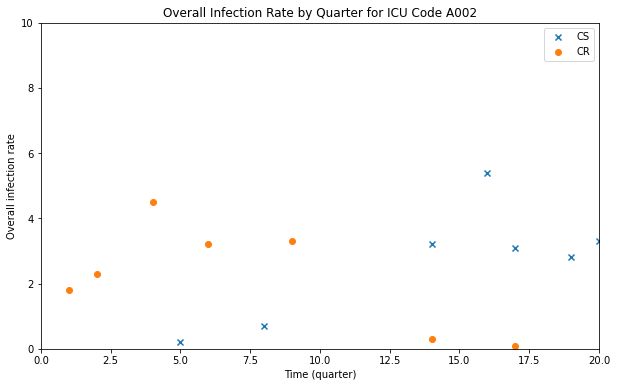

Supplement: S4 File — (ZIP) [file pone.0330765.s004.zip › Synthetic dataset/Result/KP/Pre/Figure1-1/Figure 2025-01-08 220830 (1).png]

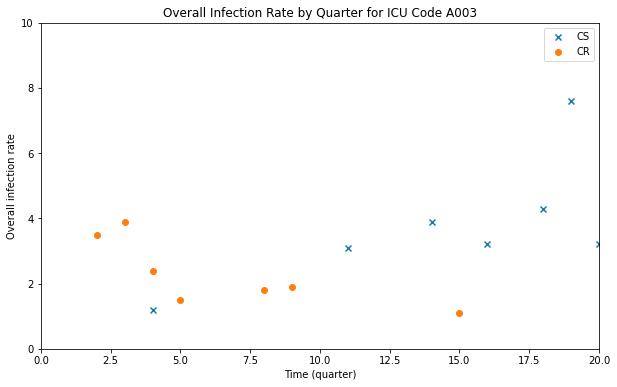

Supplement: S4 File — (ZIP) [file pone.0330765.s004.zip › Synthetic dataset/Result/KP/Pre/Figure1-1/Figure 2025-01-08 220830 (2).png]

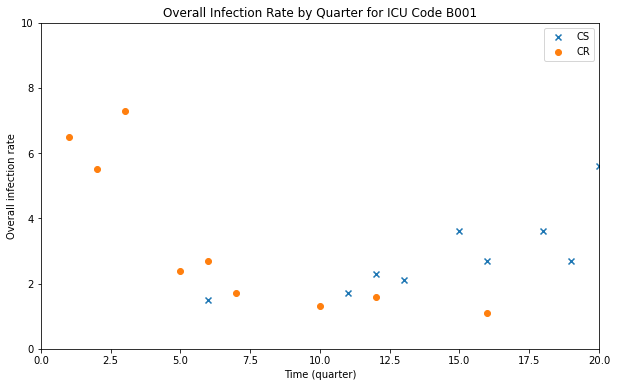

Supplement: S4 File — (ZIP) [file pone.0330765.s004.zip › Synthetic dataset/Result/KP/Pre/Figure1-1/Figure 2025-01-08 220830 (3).png]

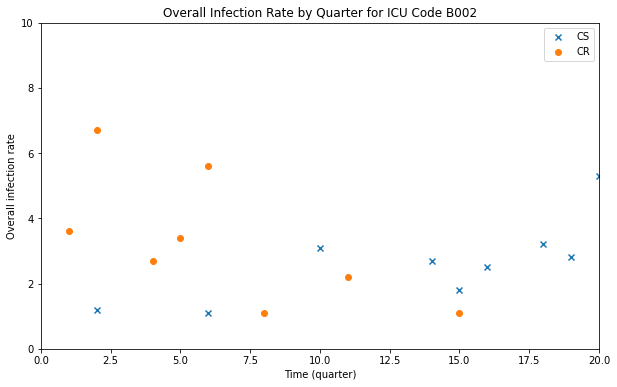

Supplement: S4 File — (ZIP) [file pone.0330765.s004.zip › Synthetic dataset/Result/KP/Pre/Figure1-1/Figure 2025-01-08 220830 (4).png]

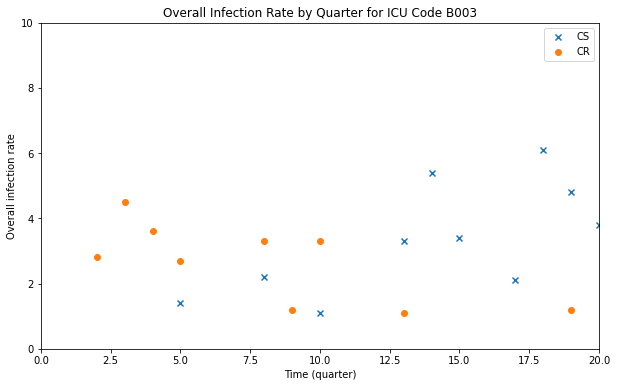

Supplement: S4 File — (ZIP) [file pone.0330765.s004.zip › Synthetic dataset/Result/KP/Pre/Figure1-1/Figure 2025-01-08 220830 (5).png]

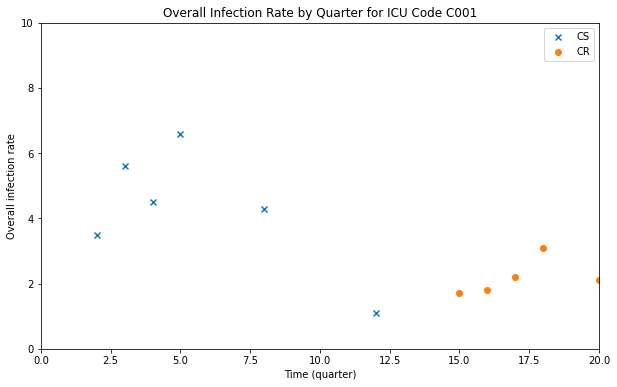

Supplement: S4 File — (ZIP) [file pone.0330765.s004.zip › Synthetic dataset/Result/KP/Pre/Figure1-1/Figure 2025-01-08 220830 (6).png]

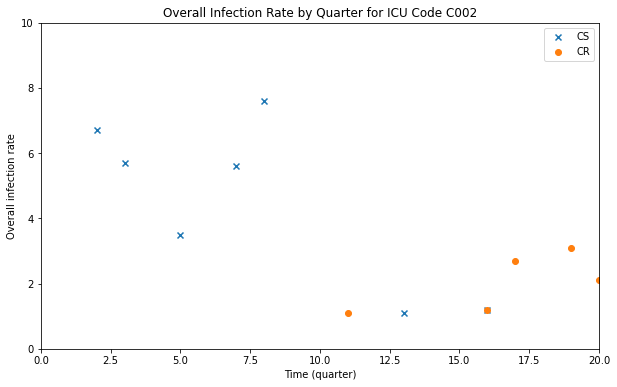

Supplement: S4 File — (ZIP) [file pone.0330765.s004.zip › Synthetic dataset/Result/KP/Pre/Figure1-1/Figure 2025-01-08 220830 (7).png]

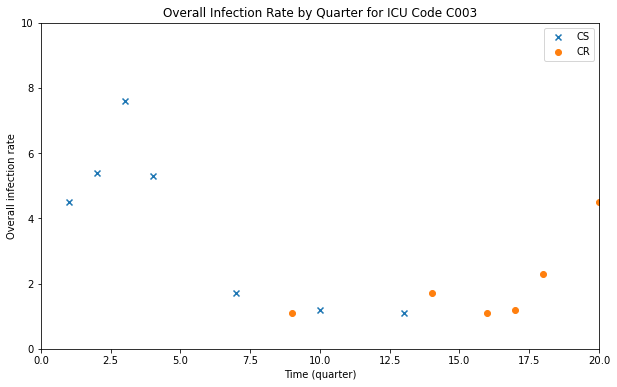

Supplement: S4 File — (ZIP) [file pone.0330765.s004.zip › Synthetic dataset/Result/KP/Pre/Figure1-1/Figure 2025-01-08 220830 (8).png]

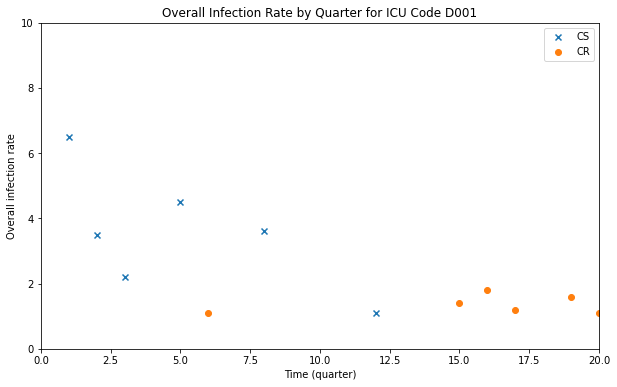

Supplement: S4 File — (ZIP) [file pone.0330765.s004.zip › Synthetic dataset/Result/KP/Pre/Figure1-1/Figure 2025-01-08 220830 (9).png]

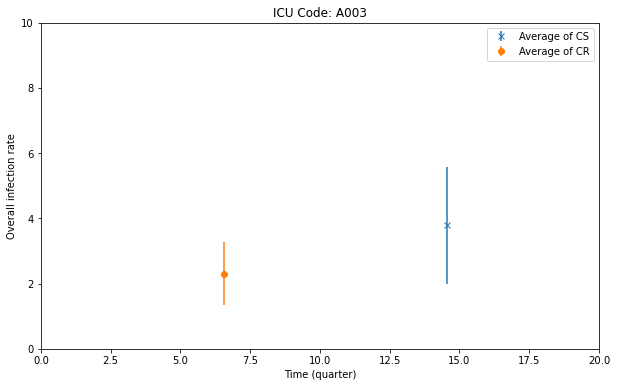

Supplement: S4 File — (ZIP) [file pone.0330765.s004.zip › Synthetic dataset/Result/KP/Pre/Figure1-2/Figure 2025-01-11 093311 (0).png]

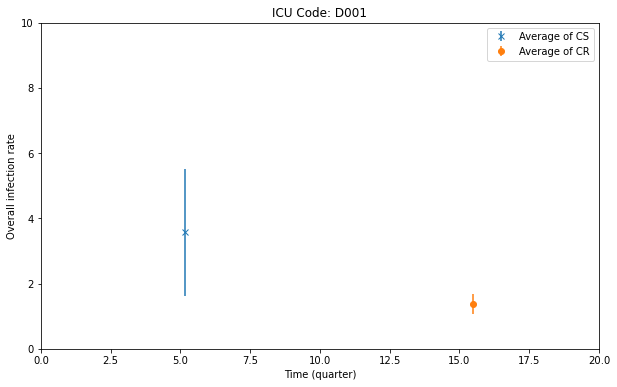

Supplement: S4 File — (ZIP) [file pone.0330765.s004.zip › Synthetic dataset/Result/KP/Pre/Figure1-2/Figure 2025-01-11 093311 (1).png]

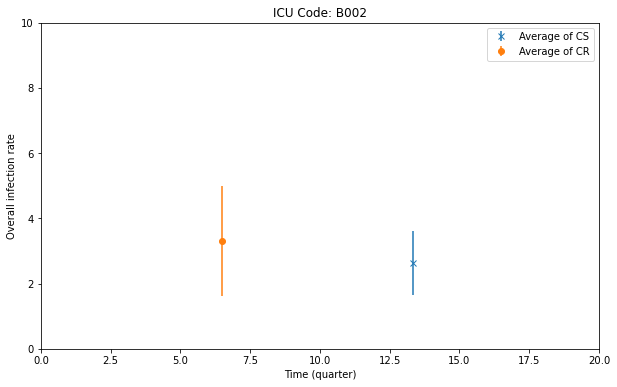

Supplement: S4 File — (ZIP) [file pone.0330765.s004.zip › Synthetic dataset/Result/KP/Pre/Figure1-2/Figure 2025-01-11 093311 (2).png]

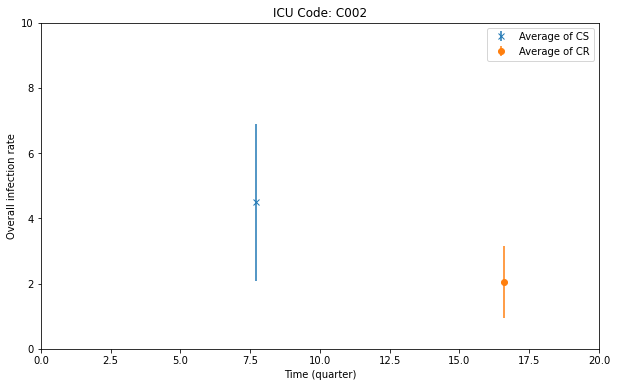

Supplement: S4 File — (ZIP) [file pone.0330765.s004.zip › Synthetic dataset/Result/KP/Pre/Figure1-2/Figure 2025-01-11 093311 (3).png]

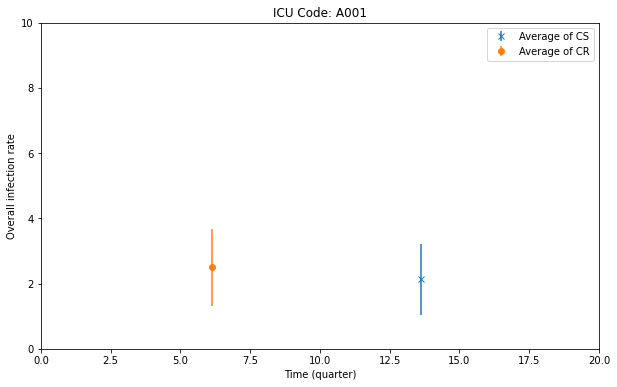

Supplement: S4 File — (ZIP) [file pone.0330765.s004.zip › Synthetic dataset/Result/KP/Pre/Figure1-2/Figure 2025-01-11 093311 (4).png]

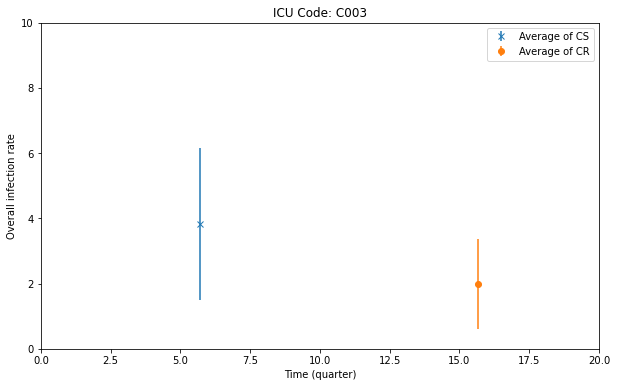

Supplement: S4 File — (ZIP) [file pone.0330765.s004.zip › Synthetic dataset/Result/KP/Pre/Figure1-2/Figure 2025-01-11 093311 (5).png]
