# Supplementary figures and images for: Visualization of multidrug-resistant bacterial infection trends in the intensive care units (part 1 of 2)
Source: PLoS One. 2025 Aug 28;20(8):e0330765. doi: 10.1371/journal.pone.0330765 (PMC12393710; doi:10.1371/journal.pone.0330765)

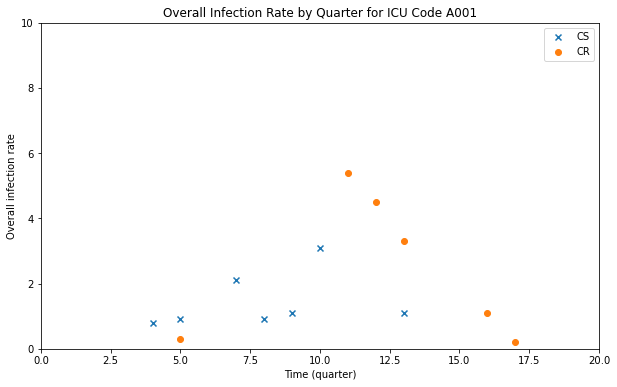

Supplement: S4 File — (ZIP) [file pone.0330765.s004.zip › Synthetic dataset/Result/AB/Post/Figure1-1/Figure 2025-01-08 234001 (0).png]

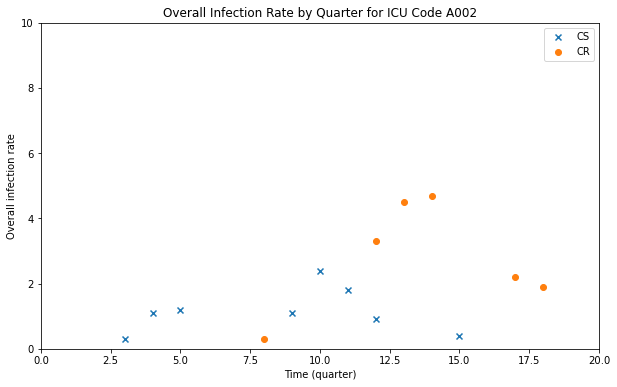

Supplement: S4 File — (ZIP) [file pone.0330765.s004.zip › Synthetic dataset/Result/AB/Post/Figure1-1/Figure 2025-01-08 234001 (1).png]

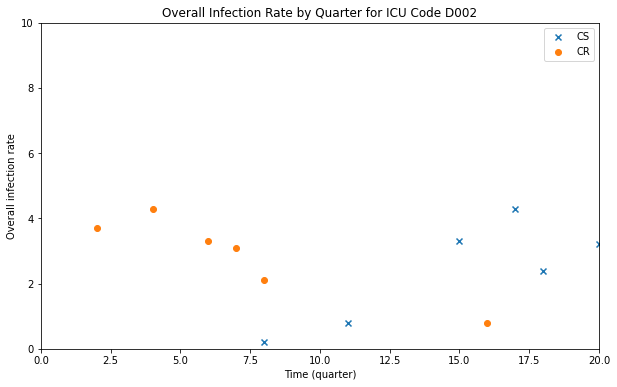

Supplement: S4 File — (ZIP) [file pone.0330765.s004.zip › Synthetic dataset/Result/AB/Post/Figure1-1/Figure 2025-01-08 234001 (10).png]

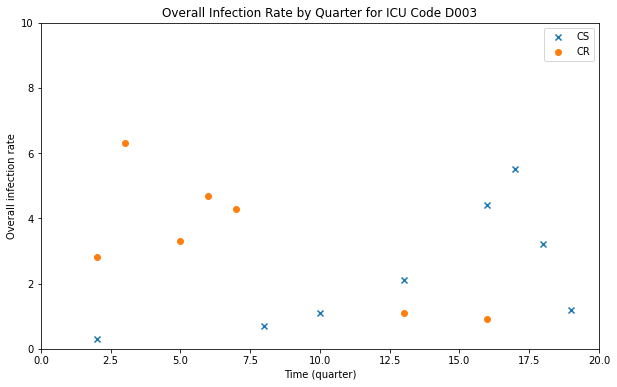

Supplement: S4 File — (ZIP) [file pone.0330765.s004.zip › Synthetic dataset/Result/AB/Post/Figure1-1/Figure 2025-01-08 234001 (11).png]

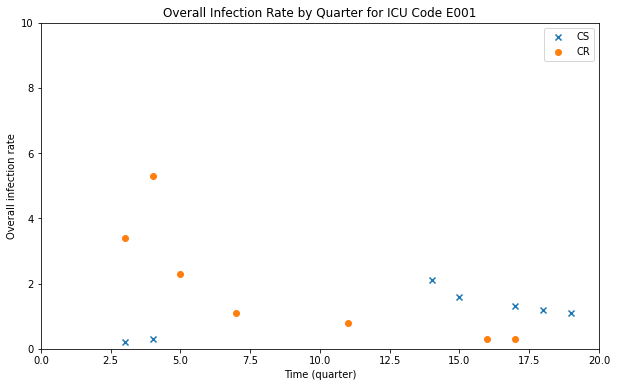

Supplement: S4 File — (ZIP) [file pone.0330765.s004.zip › Synthetic dataset/Result/AB/Post/Figure1-1/Figure 2025-01-08 234001 (12).png]

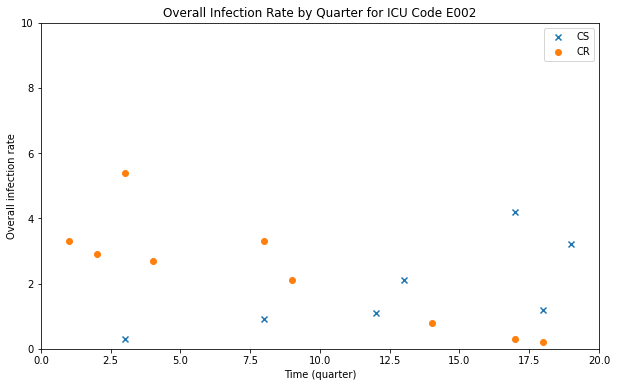

Supplement: S4 File — (ZIP) [file pone.0330765.s004.zip › Synthetic dataset/Result/AB/Post/Figure1-1/Figure 2025-01-08 234001 (13).png]

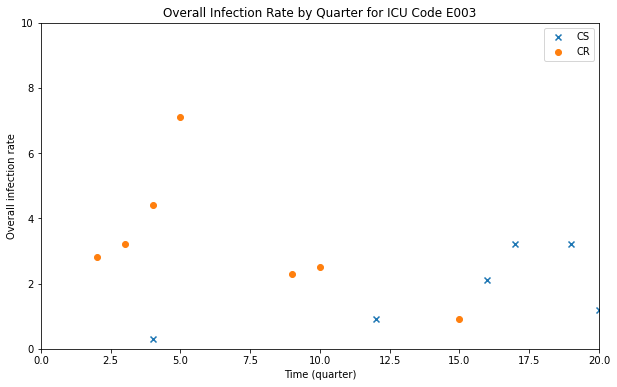

Supplement: S4 File — (ZIP) [file pone.0330765.s004.zip › Synthetic dataset/Result/AB/Post/Figure1-1/Figure 2025-01-08 234001 (14).png]

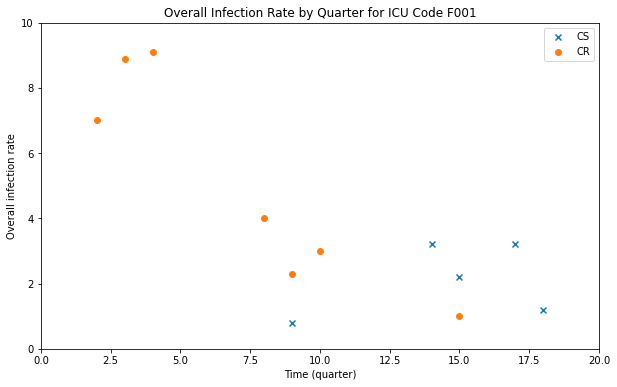

Supplement: S4 File — (ZIP) [file pone.0330765.s004.zip › Synthetic dataset/Result/AB/Post/Figure1-1/Figure 2025-01-08 234001 (15).png]

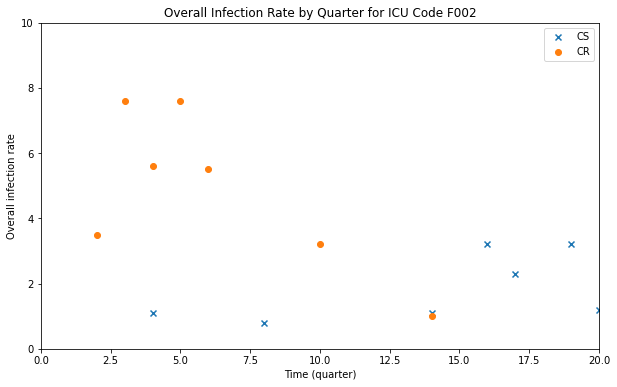

Supplement: S4 File — (ZIP) [file pone.0330765.s004.zip › Synthetic dataset/Result/AB/Post/Figure1-1/Figure 2025-01-08 234001 (16).png]

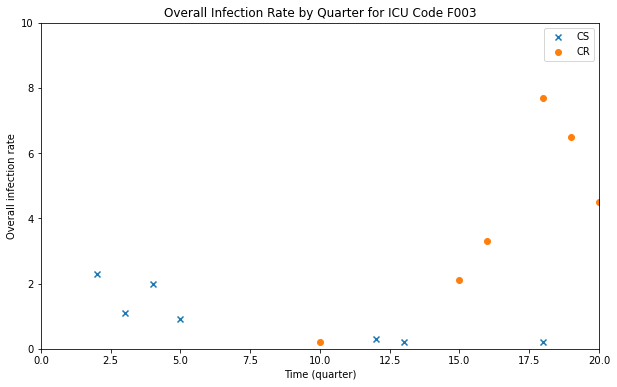

Supplement: S4 File — (ZIP) [file pone.0330765.s004.zip › Synthetic dataset/Result/AB/Post/Figure1-1/Figure 2025-01-08 234001 (17).png]

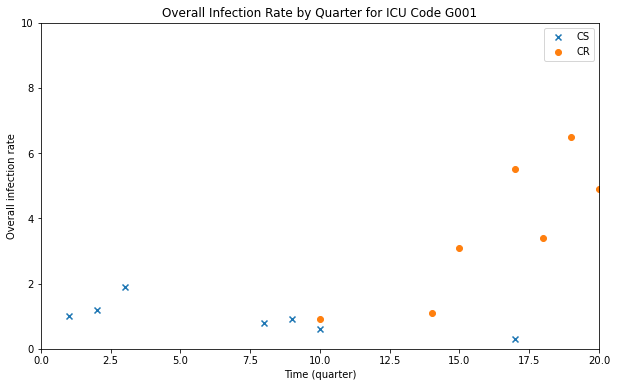

Supplement: S4 File — (ZIP) [file pone.0330765.s004.zip › Synthetic dataset/Result/AB/Post/Figure1-1/Figure 2025-01-08 234001 (18).png]

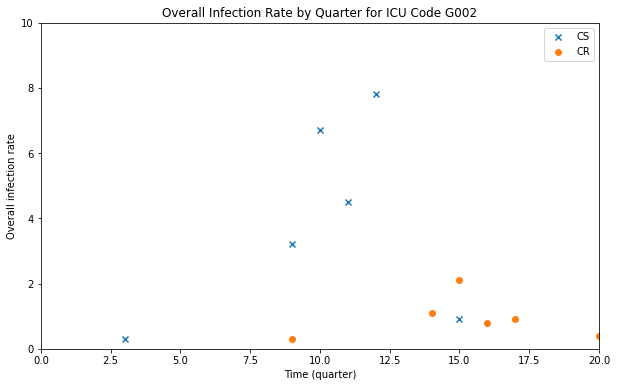

Supplement: S4 File — (ZIP) [file pone.0330765.s004.zip › Synthetic dataset/Result/AB/Post/Figure1-1/Figure 2025-01-08 234001 (19).png]

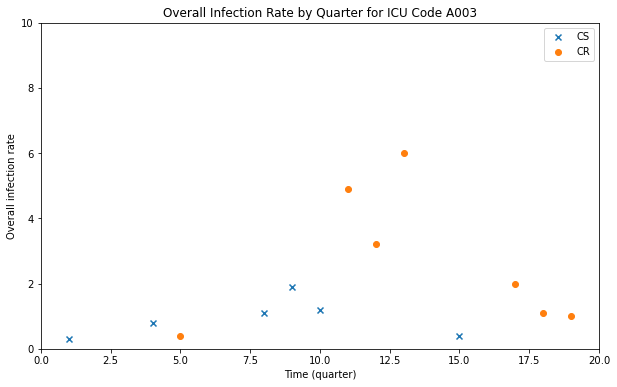

Supplement: S4 File — (ZIP) [file pone.0330765.s004.zip › Synthetic dataset/Result/AB/Post/Figure1-1/Figure 2025-01-08 234001 (2).png]

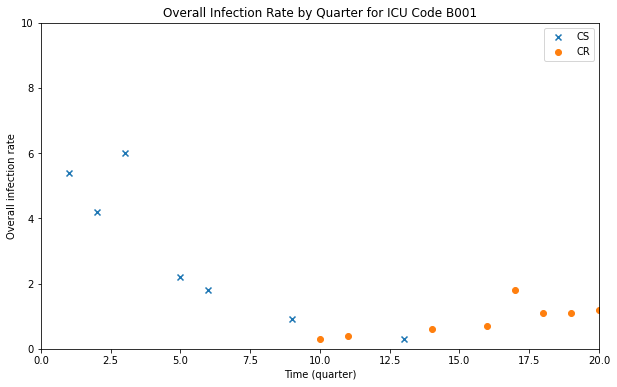

Supplement: S4 File — (ZIP) [file pone.0330765.s004.zip › Synthetic dataset/Result/AB/Post/Figure1-1/Figure 2025-01-08 234001 (3).png]

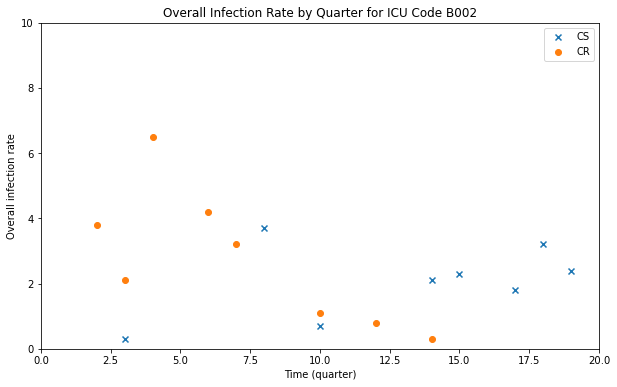

Supplement: S4 File — (ZIP) [file pone.0330765.s004.zip › Synthetic dataset/Result/AB/Post/Figure1-1/Figure 2025-01-08 234001 (4).png]

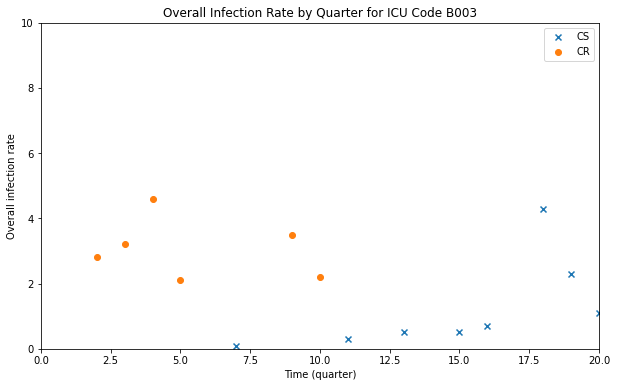

Supplement: S4 File — (ZIP) [file pone.0330765.s004.zip › Synthetic dataset/Result/AB/Post/Figure1-1/Figure 2025-01-08 234001 (5).png]

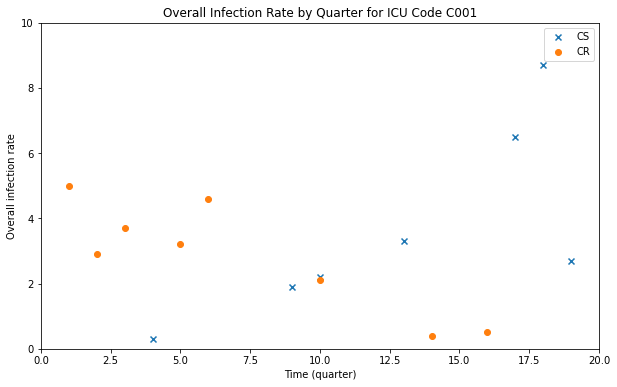

Supplement: S4 File — (ZIP) [file pone.0330765.s004.zip › Synthetic dataset/Result/AB/Post/Figure1-1/Figure 2025-01-08 234001 (6).png]

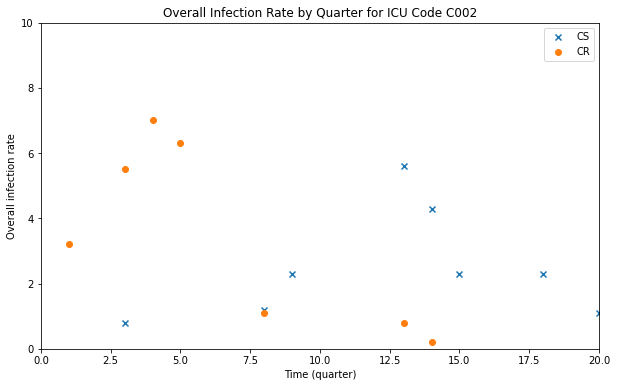

Supplement: S4 File — (ZIP) [file pone.0330765.s004.zip › Synthetic dataset/Result/AB/Post/Figure1-1/Figure 2025-01-08 234001 (7).png]

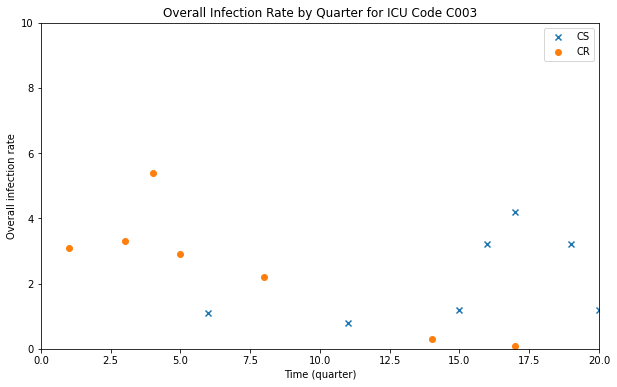

Supplement: S4 File — (ZIP) [file pone.0330765.s004.zip › Synthetic dataset/Result/AB/Post/Figure1-1/Figure 2025-01-08 234001 (8).png]

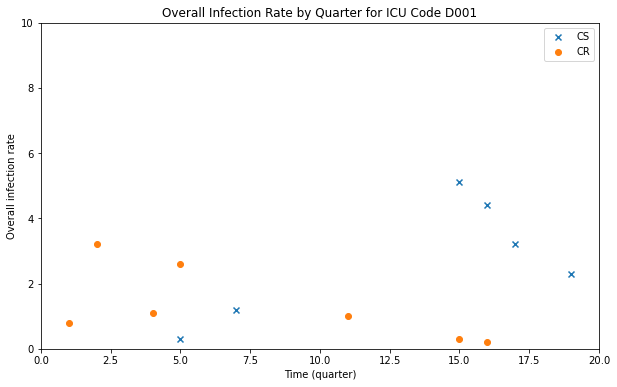

Supplement: S4 File — (ZIP) [file pone.0330765.s004.zip › Synthetic dataset/Result/AB/Post/Figure1-1/Figure 2025-01-08 234001 (9).png]

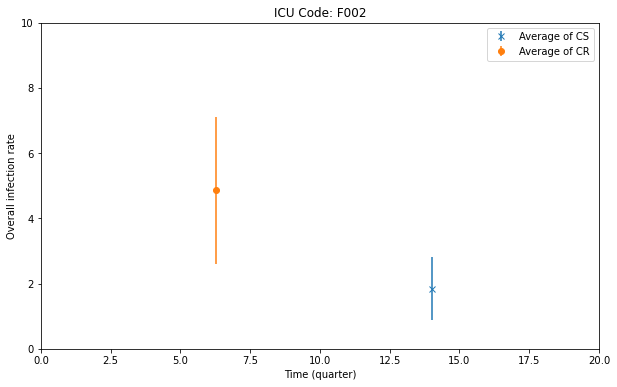

Supplement: S4 File — (ZIP) [file pone.0330765.s004.zip › Synthetic dataset/Result/AB/Post/Figure1-2/Figure 2025-01-11 093723 (0).png]

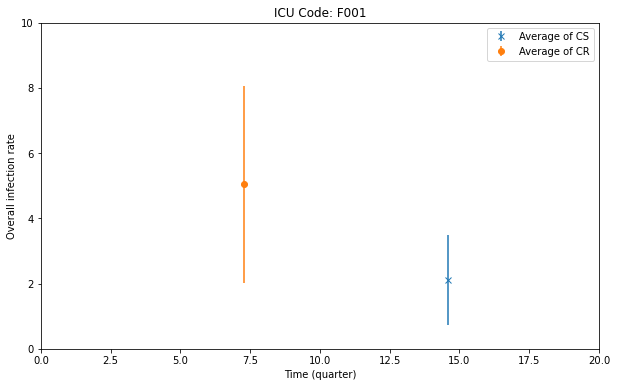

Supplement: S4 File — (ZIP) [file pone.0330765.s004.zip › Synthetic dataset/Result/AB/Post/Figure1-2/Figure 2025-01-11 093723 (1).png]

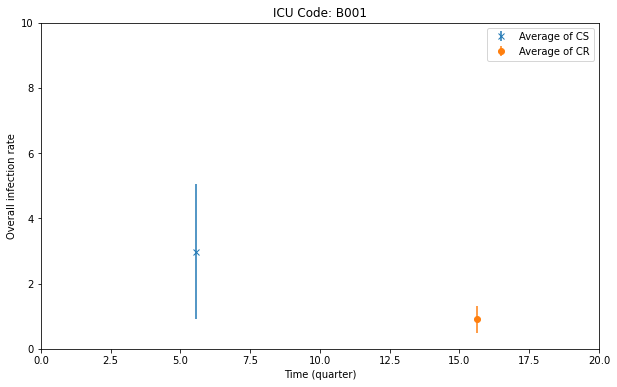

Supplement: S4 File — (ZIP) [file pone.0330765.s004.zip › Synthetic dataset/Result/AB/Post/Figure1-2/Figure 2025-01-11 093723 (10).png]

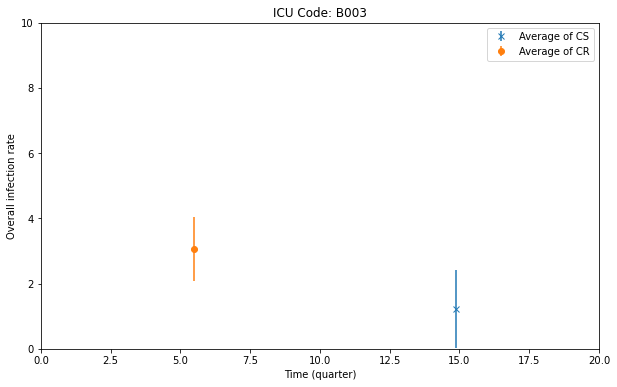

Supplement: S4 File — (ZIP) [file pone.0330765.s004.zip › Synthetic dataset/Result/AB/Post/Figure1-2/Figure 2025-01-11 093723 (11).png]

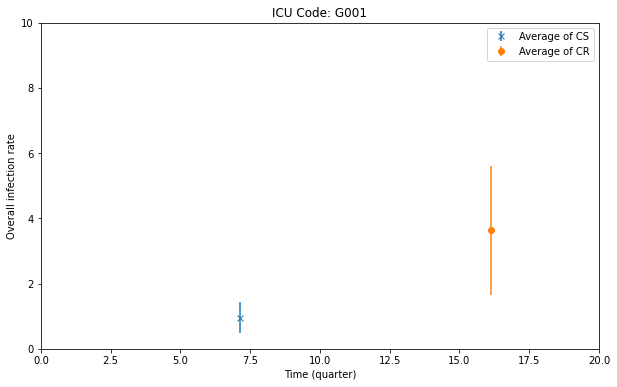

Supplement: S4 File — (ZIP) [file pone.0330765.s004.zip › Synthetic dataset/Result/AB/Post/Figure1-2/Figure 2025-01-11 093723 (12).png]

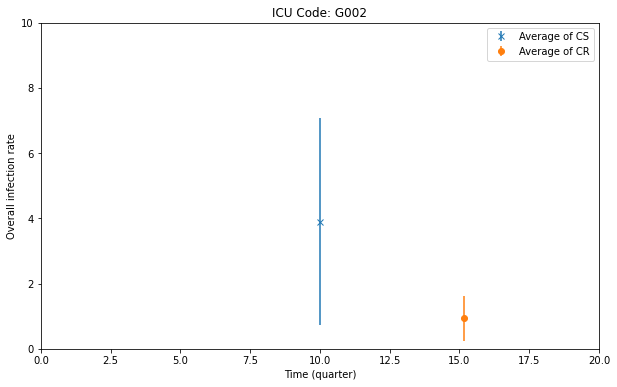

Supplement: S4 File — (ZIP) [file pone.0330765.s004.zip › Synthetic dataset/Result/AB/Post/Figure1-2/Figure 2025-01-11 093723 (13).png]

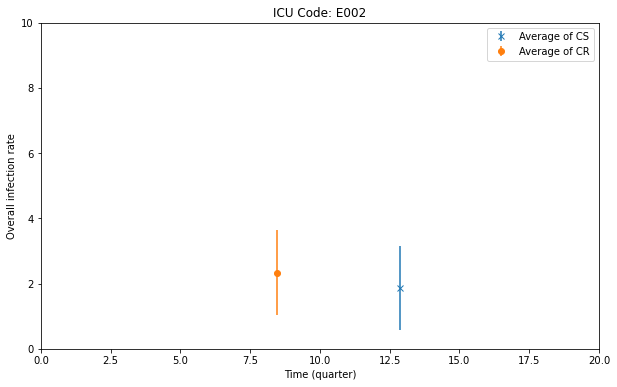

Supplement: S4 File — (ZIP) [file pone.0330765.s004.zip › Synthetic dataset/Result/AB/Post/Figure1-2/Figure 2025-01-11 093723 (14).png]

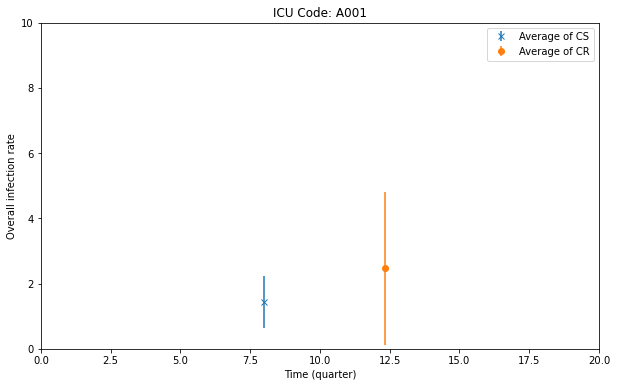

Supplement: S4 File — (ZIP) [file pone.0330765.s004.zip › Synthetic dataset/Result/AB/Post/Figure1-2/Figure 2025-01-11 093723 (15).png]

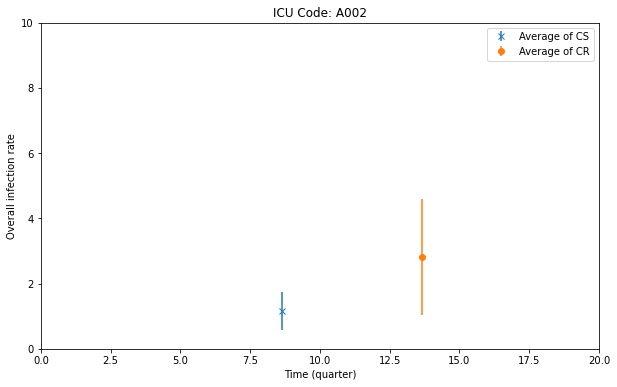

Supplement: S4 File — (ZIP) [file pone.0330765.s004.zip › Synthetic dataset/Result/AB/Post/Figure1-2/Figure 2025-01-11 093723 (16).png]

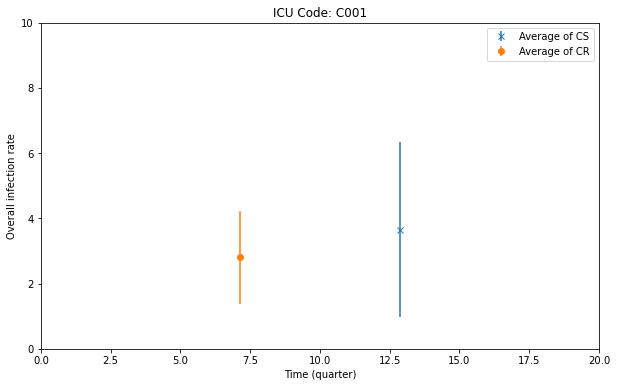

Supplement: S4 File — (ZIP) [file pone.0330765.s004.zip › Synthetic dataset/Result/AB/Post/Figure1-2/Figure 2025-01-11 093723 (17).png]

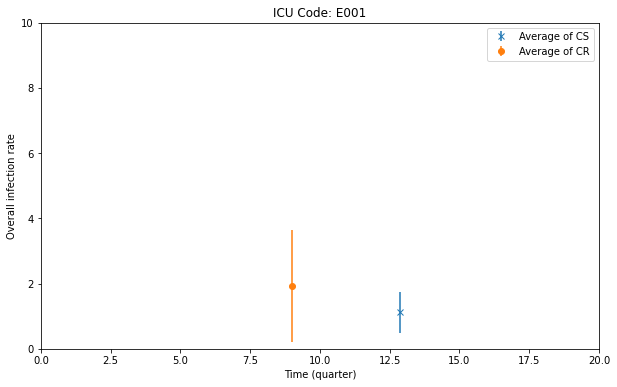

Supplement: S4 File — (ZIP) [file pone.0330765.s004.zip › Synthetic dataset/Result/AB/Post/Figure1-2/Figure 2025-01-11 093723 (18).png]

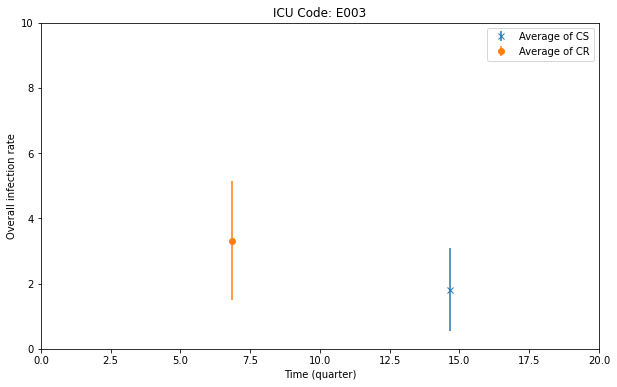

Supplement: S4 File — (ZIP) [file pone.0330765.s004.zip › Synthetic dataset/Result/AB/Post/Figure1-2/Figure 2025-01-11 093723 (19).png]

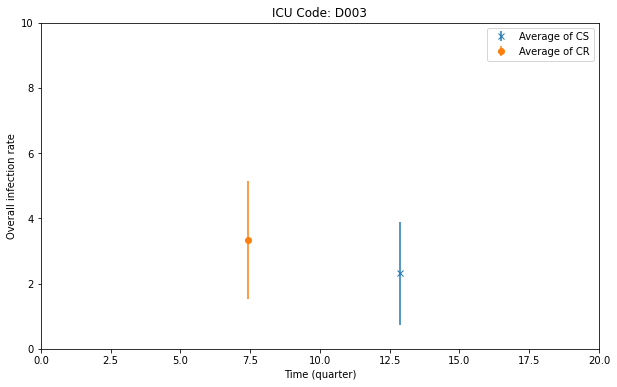

Supplement: S4 File — (ZIP) [file pone.0330765.s004.zip › Synthetic dataset/Result/AB/Post/Figure1-2/Figure 2025-01-11 093723 (2).png]

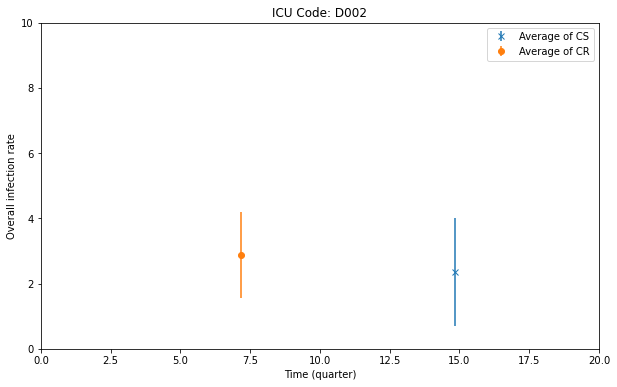

Supplement: S4 File — (ZIP) [file pone.0330765.s004.zip › Synthetic dataset/Result/AB/Post/Figure1-2/Figure 2025-01-11 093723 (3).png]

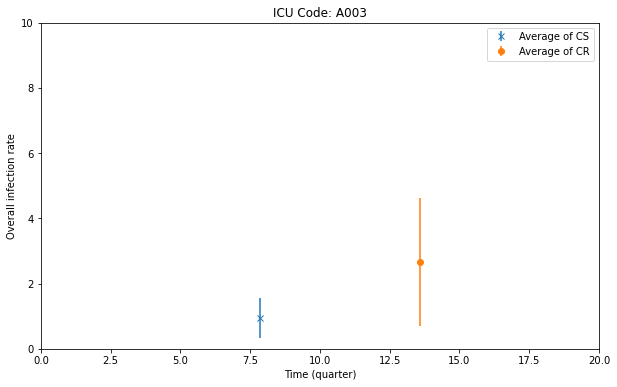

Supplement: S4 File — (ZIP) [file pone.0330765.s004.zip › Synthetic dataset/Result/AB/Post/Figure1-2/Figure 2025-01-11 093723 (4).png]

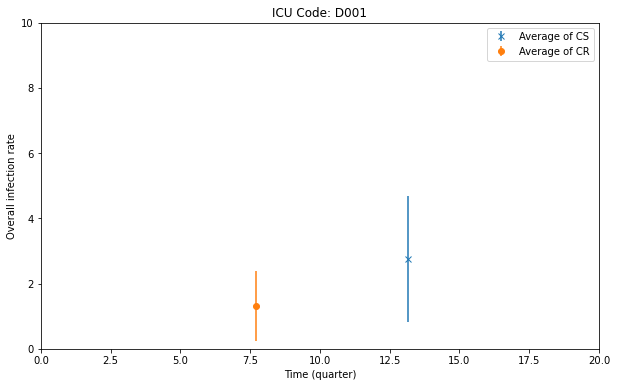

Supplement: S4 File — (ZIP) [file pone.0330765.s004.zip › Synthetic dataset/Result/AB/Post/Figure1-2/Figure 2025-01-11 093723 (5).png]

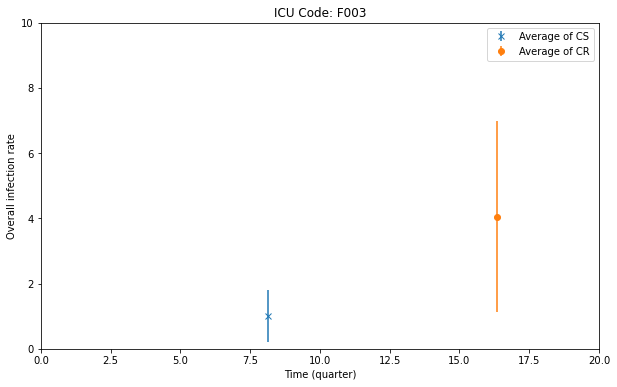

Supplement: S4 File — (ZIP) [file pone.0330765.s004.zip › Synthetic dataset/Result/AB/Post/Figure1-2/Figure 2025-01-11 093723 (6).png]

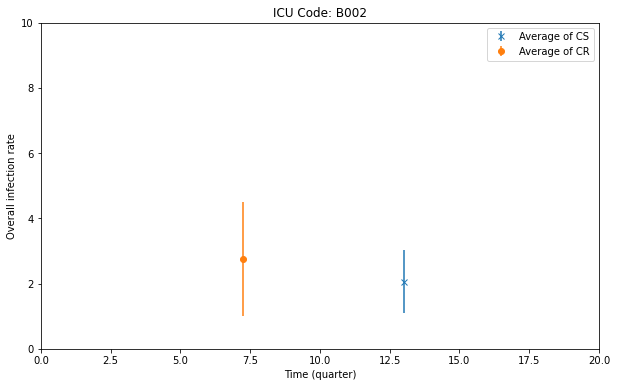

Supplement: S4 File — (ZIP) [file pone.0330765.s004.zip › Synthetic dataset/Result/AB/Post/Figure1-2/Figure 2025-01-11 093723 (7).png]

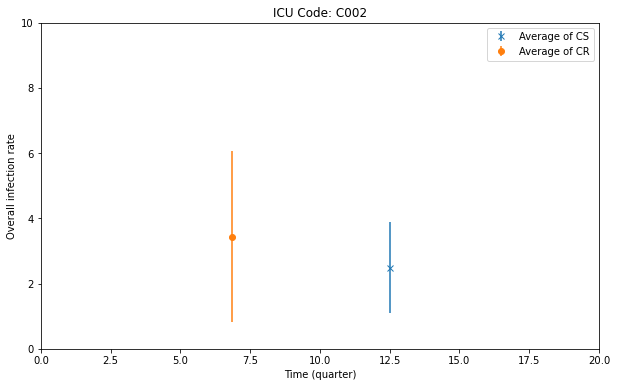

Supplement: S4 File — (ZIP) [file pone.0330765.s004.zip › Synthetic dataset/Result/AB/Post/Figure1-2/Figure 2025-01-11 093723 (8).png]

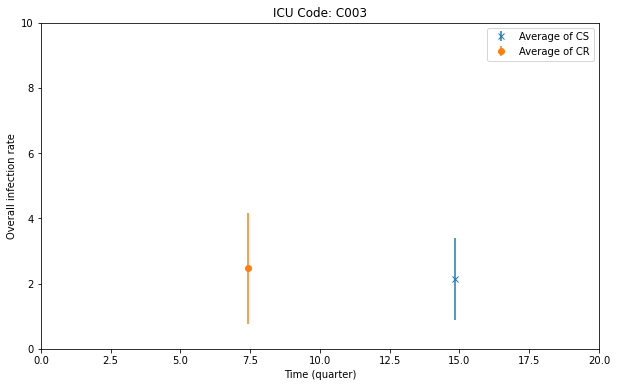

Supplement: S4 File — (ZIP) [file pone.0330765.s004.zip › Synthetic dataset/Result/AB/Post/Figure1-2/Figure 2025-01-11 093723 (9).png]

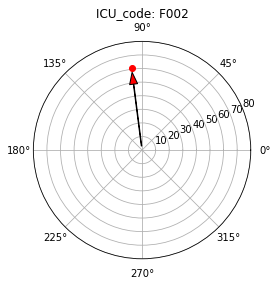

Supplement: S4 File — (ZIP) [file pone.0330765.s004.zip › Synthetic dataset/Result/AB/Post/Figure1-3/Figure 2025-01-11 104456 (0).png]

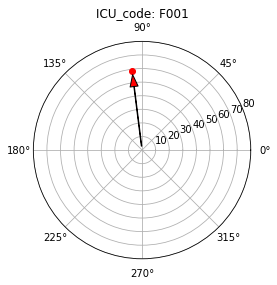

Supplement: S4 File — (ZIP) [file pone.0330765.s004.zip › Synthetic dataset/Result/AB/Post/Figure1-3/Figure 2025-01-11 104456 (1).png]

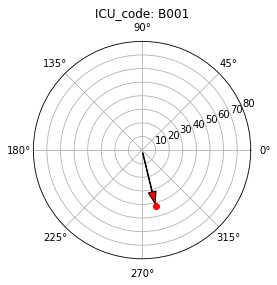

Supplement: S4 File — (ZIP) [file pone.0330765.s004.zip › Synthetic dataset/Result/AB/Post/Figure1-3/Figure 2025-01-11 104456 (10).png]

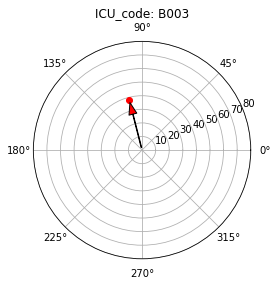

Supplement: S4 File — (ZIP) [file pone.0330765.s004.zip › Synthetic dataset/Result/AB/Post/Figure1-3/Figure 2025-01-11 104456 (11).png]

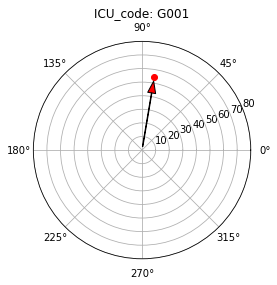

Supplement: S4 File — (ZIP) [file pone.0330765.s004.zip › Synthetic dataset/Result/AB/Post/Figure1-3/Figure 2025-01-11 104456 (12).png]

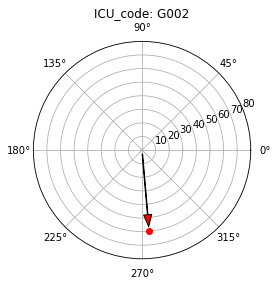

Supplement: S4 File — (ZIP) [file pone.0330765.s004.zip › Synthetic dataset/Result/AB/Post/Figure1-3/Figure 2025-01-11 104456 (13).png]

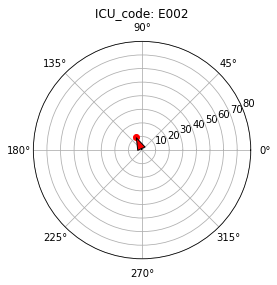

Supplement: S4 File — (ZIP) [file pone.0330765.s004.zip › Synthetic dataset/Result/AB/Post/Figure1-3/Figure 2025-01-11 104456 (14).png]

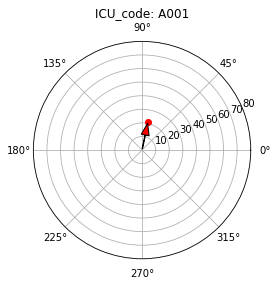

Supplement: S4 File — (ZIP) [file pone.0330765.s004.zip › Synthetic dataset/Result/AB/Post/Figure1-3/Figure 2025-01-11 104456 (15).png]

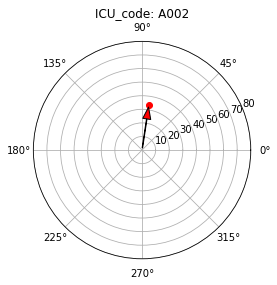

Supplement: S4 File — (ZIP) [file pone.0330765.s004.zip › Synthetic dataset/Result/AB/Post/Figure1-3/Figure 2025-01-11 104456 (16).png]

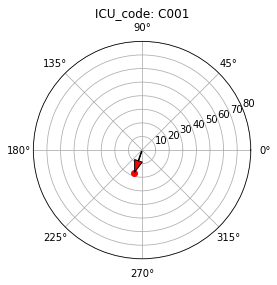

Supplement: S4 File — (ZIP) [file pone.0330765.s004.zip › Synthetic dataset/Result/AB/Post/Figure1-3/Figure 2025-01-11 104456 (17).png]

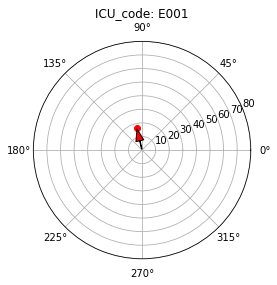

Supplement: S4 File — (ZIP) [file pone.0330765.s004.zip › Synthetic dataset/Result/AB/Post/Figure1-3/Figure 2025-01-11 104456 (18).png]

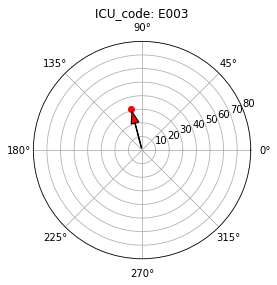

Supplement: S4 File — (ZIP) [file pone.0330765.s004.zip › Synthetic dataset/Result/AB/Post/Figure1-3/Figure 2025-01-11 104456 (19).png]

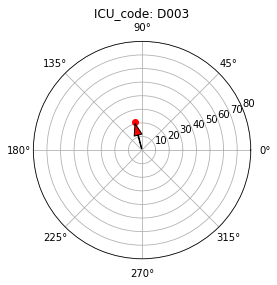

Supplement: S4 File — (ZIP) [file pone.0330765.s004.zip › Synthetic dataset/Result/AB/Post/Figure1-3/Figure 2025-01-11 104456 (2).png]

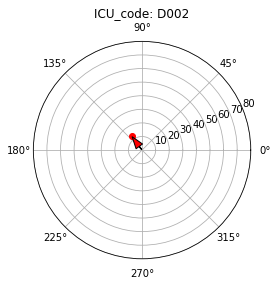

Supplement: S4 File — (ZIP) [file pone.0330765.s004.zip › Synthetic dataset/Result/AB/Post/Figure1-3/Figure 2025-01-11 104456 (3).png]

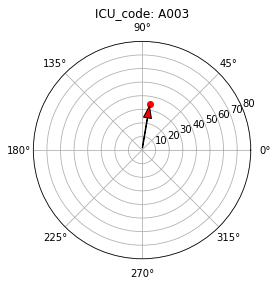

Supplement: S4 File — (ZIP) [file pone.0330765.s004.zip › Synthetic dataset/Result/AB/Post/Figure1-3/Figure 2025-01-11 104456 (4).png]

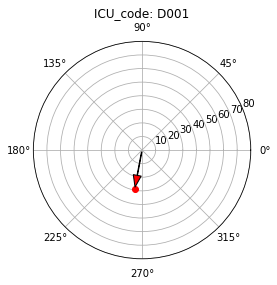

Supplement: S4 File — (ZIP) [file pone.0330765.s004.zip › Synthetic dataset/Result/AB/Post/Figure1-3/Figure 2025-01-11 104456 (5).png]

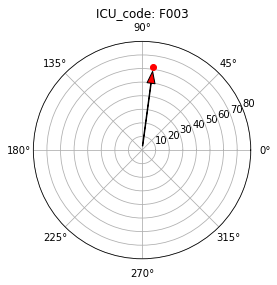

Supplement: S4 File — (ZIP) [file pone.0330765.s004.zip › Synthetic dataset/Result/AB/Post/Figure1-3/Figure 2025-01-11 104456 (6).png]

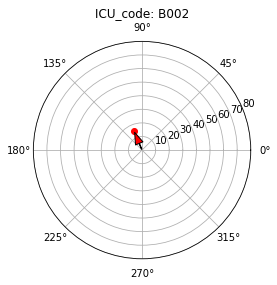

Supplement: S4 File — (ZIP) [file pone.0330765.s004.zip › Synthetic dataset/Result/AB/Post/Figure1-3/Figure 2025-01-11 104456 (7).png]

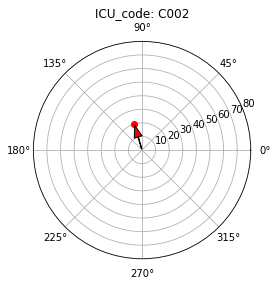

Supplement: S4 File — (ZIP) [file pone.0330765.s004.zip › Synthetic dataset/Result/AB/Post/Figure1-3/Figure 2025-01-11 104456 (8).png]

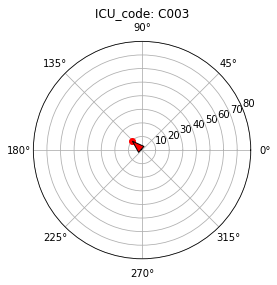

Supplement: S4 File — (ZIP) [file pone.0330765.s004.zip › Synthetic dataset/Result/AB/Post/Figure1-3/Figure 2025-01-11 104456 (9).png]

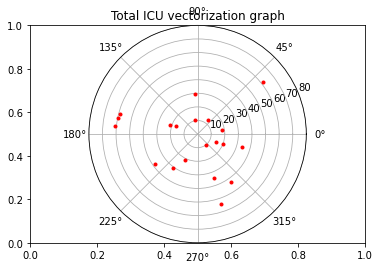

Supplement: S4 File — (ZIP) [file pone.0330765.s004.zip › Synthetic dataset/Result/AB/Post/Figure4/Figure 2025-01-11 104923 (0).png]

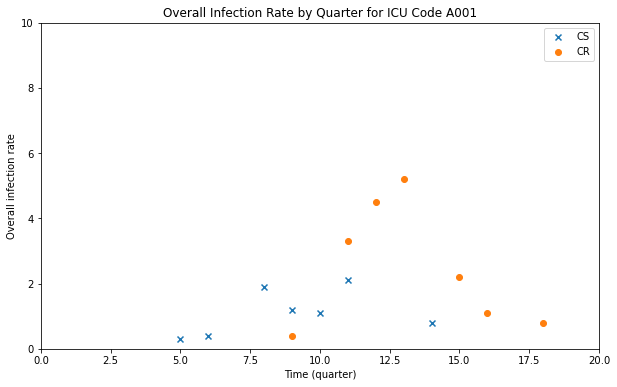

Supplement: S4 File — (ZIP) [file pone.0330765.s004.zip › Synthetic dataset/Result/AB/Pre/Figure1-1/Figure 2025-01-08 233935 (0).png]

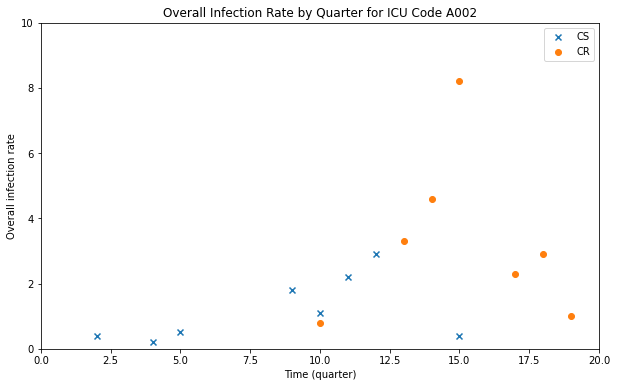

Supplement: S4 File — (ZIP) [file pone.0330765.s004.zip › Synthetic dataset/Result/AB/Pre/Figure1-1/Figure 2025-01-08 233935 (1).png]

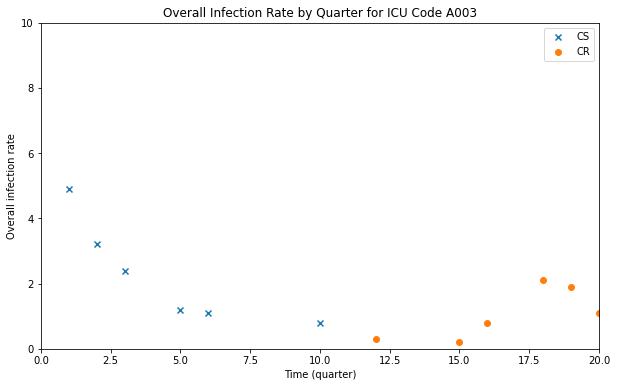

Supplement: S4 File — (ZIP) [file pone.0330765.s004.zip › Synthetic dataset/Result/AB/Pre/Figure1-1/Figure 2025-01-08 233935 (2).png]

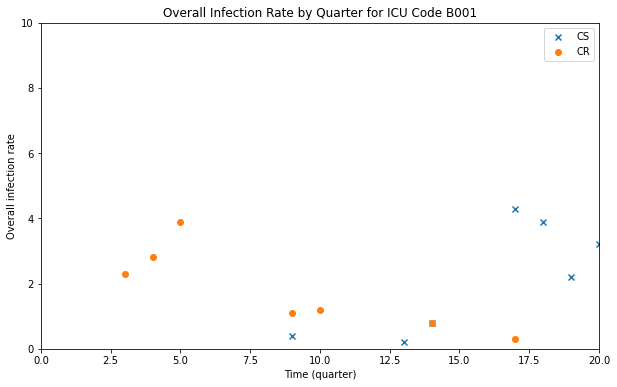

Supplement: S4 File — (ZIP) [file pone.0330765.s004.zip › Synthetic dataset/Result/AB/Pre/Figure1-1/Figure 2025-01-08 233935 (3).png]

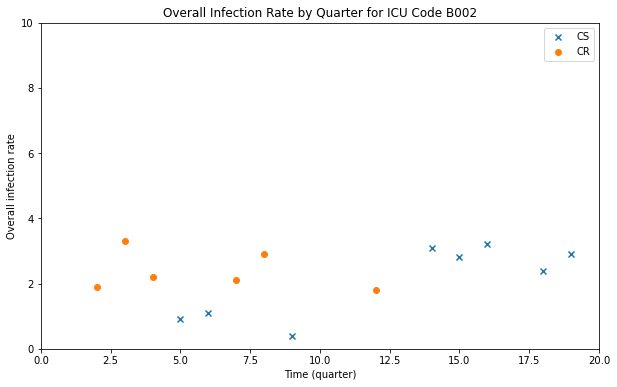

Supplement: S4 File — (ZIP) [file pone.0330765.s004.zip › Synthetic dataset/Result/AB/Pre/Figure1-1/Figure 2025-01-08 233935 (4).png]

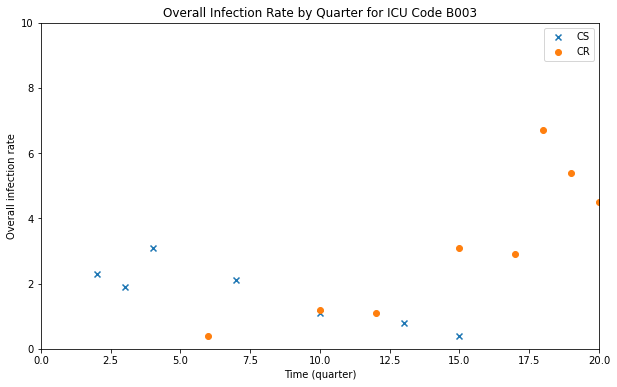

Supplement: S4 File — (ZIP) [file pone.0330765.s004.zip › Synthetic dataset/Result/AB/Pre/Figure1-1/Figure 2025-01-08 233935 (5).png]

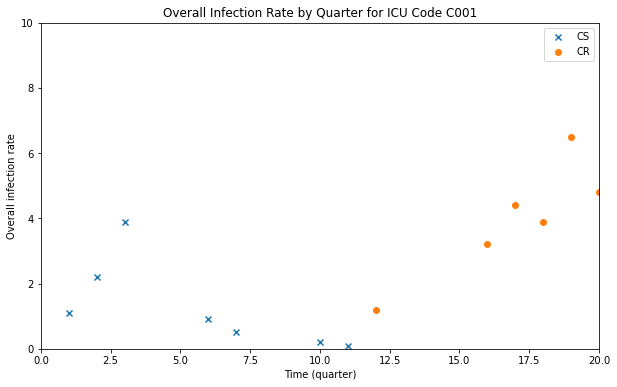

Supplement: S4 File — (ZIP) [file pone.0330765.s004.zip › Synthetic dataset/Result/AB/Pre/Figure1-1/Figure 2025-01-08 233935 (6).png]

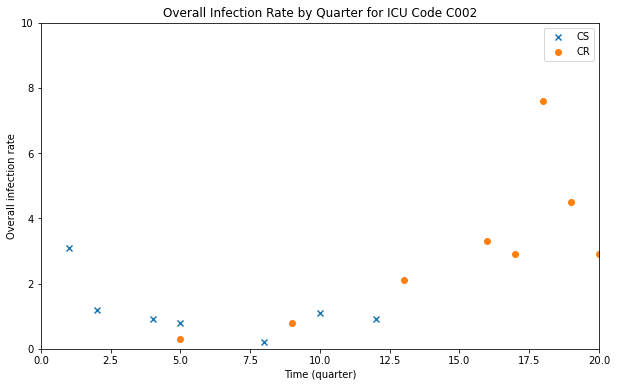

Supplement: S4 File — (ZIP) [file pone.0330765.s004.zip › Synthetic dataset/Result/AB/Pre/Figure1-1/Figure 2025-01-08 233935 (7).png]

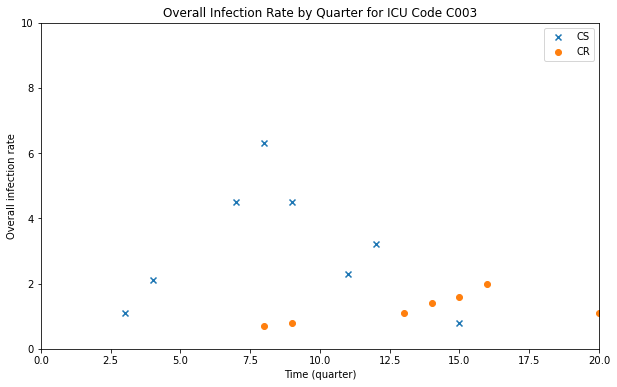

Supplement: S4 File — (ZIP) [file pone.0330765.s004.zip › Synthetic dataset/Result/AB/Pre/Figure1-1/Figure 2025-01-08 233935 (8).png]

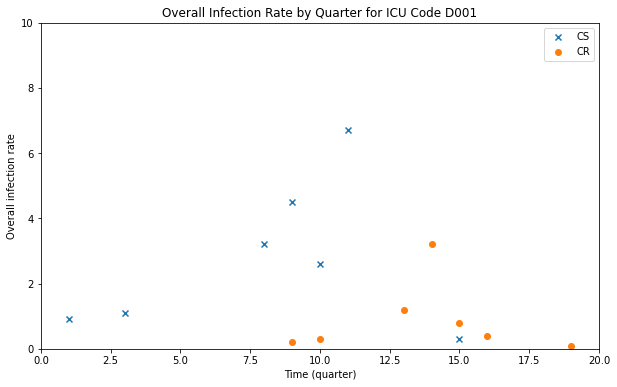

Supplement: S4 File — (ZIP) [file pone.0330765.s004.zip › Synthetic dataset/Result/AB/Pre/Figure1-1/Figure 2025-01-08 233935 (9).png]

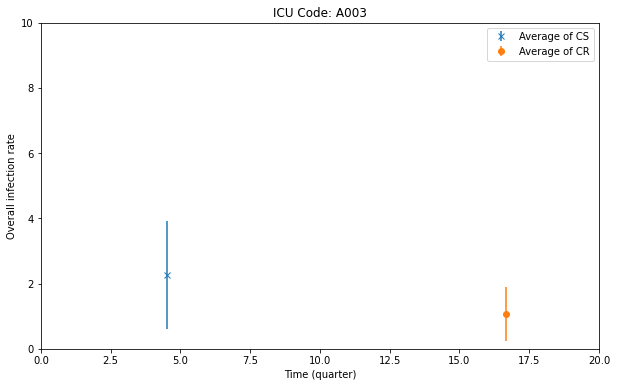

Supplement: S4 File — (ZIP) [file pone.0330765.s004.zip › Synthetic dataset/Result/AB/Pre/Figure1-2/Figure 2025-01-11 093640 (0).png]

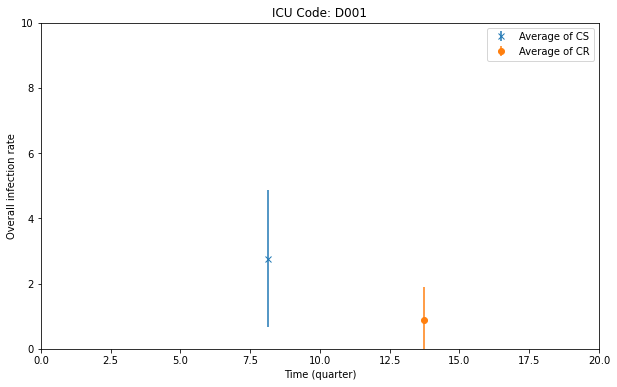

Supplement: S4 File — (ZIP) [file pone.0330765.s004.zip › Synthetic dataset/Result/AB/Pre/Figure1-2/Figure 2025-01-11 093640 (1).png]

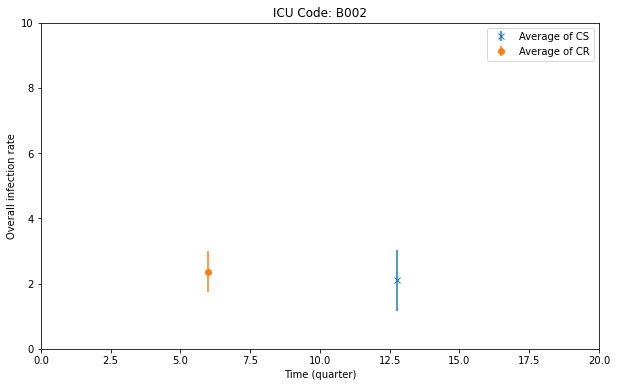

Supplement: S4 File — (ZIP) [file pone.0330765.s004.zip › Synthetic dataset/Result/AB/Pre/Figure1-2/Figure 2025-01-11 093640 (2).png]

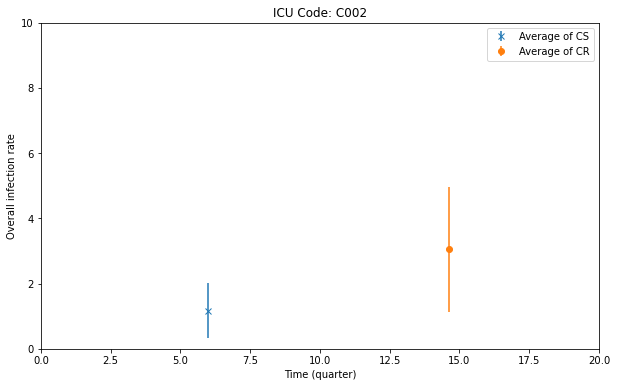

Supplement: S4 File — (ZIP) [file pone.0330765.s004.zip › Synthetic dataset/Result/AB/Pre/Figure1-2/Figure 2025-01-11 093640 (3).png]

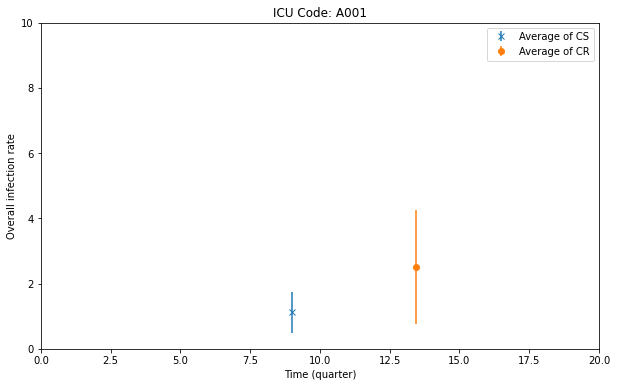

Supplement: S4 File — (ZIP) [file pone.0330765.s004.zip › Synthetic dataset/Result/AB/Pre/Figure1-2/Figure 2025-01-11 093640 (4).png]

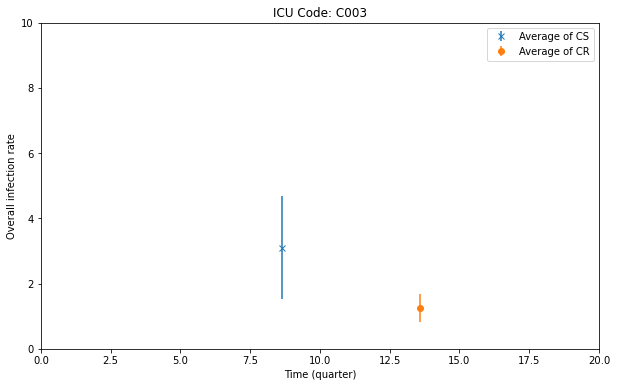

Supplement: S4 File — (ZIP) [file pone.0330765.s004.zip › Synthetic dataset/Result/AB/Pre/Figure1-2/Figure 2025-01-11 093640 (5).png]

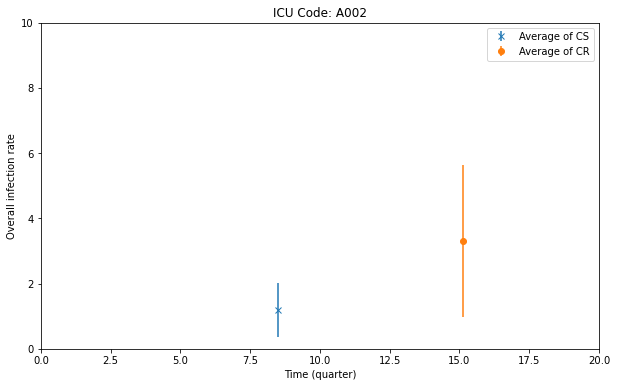

Supplement: S4 File — (ZIP) [file pone.0330765.s004.zip › Synthetic dataset/Result/AB/Pre/Figure1-2/Figure 2025-01-11 093640 (6).png]

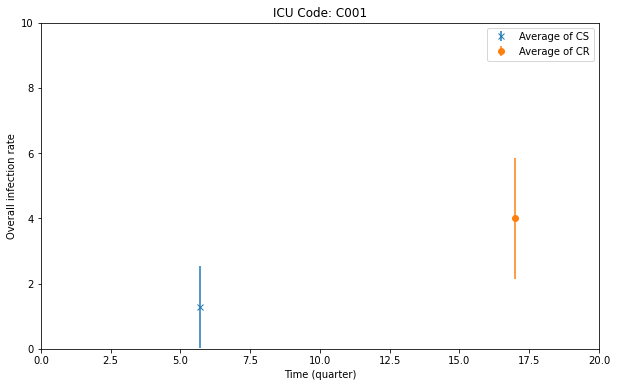

Supplement: S4 File — (ZIP) [file pone.0330765.s004.zip › Synthetic dataset/Result/AB/Pre/Figure1-2/Figure 2025-01-11 093640 (7).png]

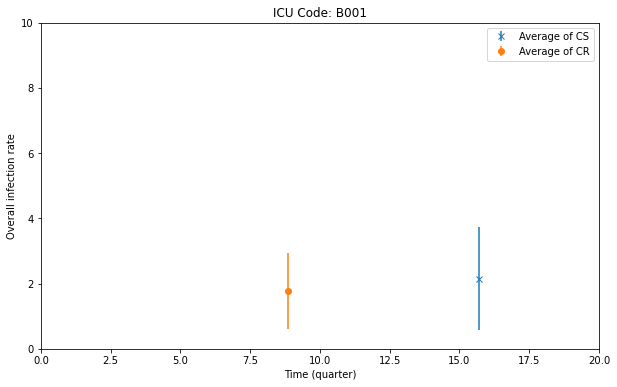

Supplement: S4 File — (ZIP) [file pone.0330765.s004.zip › Synthetic dataset/Result/AB/Pre/Figure1-2/Figure 2025-01-11 093640 (8).png]

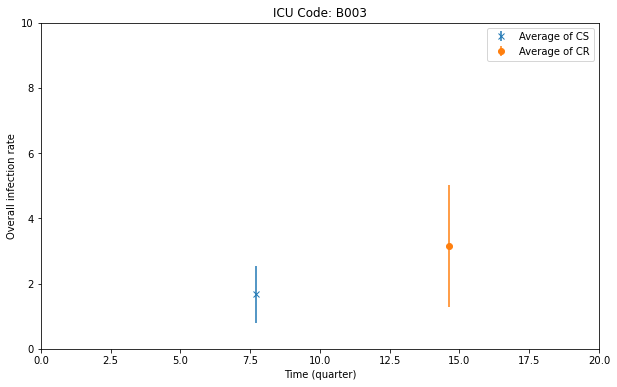

Supplement: S4 File — (ZIP) [file pone.0330765.s004.zip › Synthetic dataset/Result/AB/Pre/Figure1-2/Figure 2025-01-11 093640 (9).png]

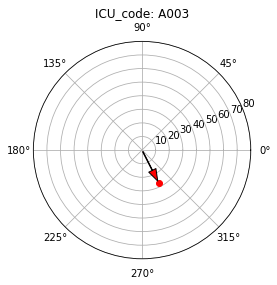

Supplement: S4 File — (ZIP) [file pone.0330765.s004.zip › Synthetic dataset/Result/AB/Pre/Figure1-3/Figure 2025-01-11 104418 (0).png]

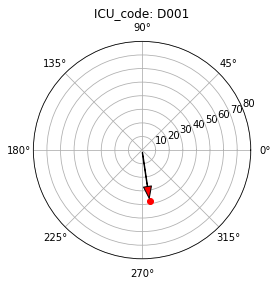

Supplement: S4 File — (ZIP) [file pone.0330765.s004.zip › Synthetic dataset/Result/AB/Pre/Figure1-3/Figure 2025-01-11 104418 (1).png]

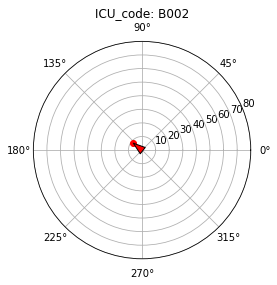

Supplement: S4 File — (ZIP) [file pone.0330765.s004.zip › Synthetic dataset/Result/AB/Pre/Figure1-3/Figure 2025-01-11 104418 (2).png]

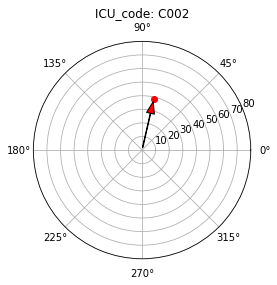

Supplement: S4 File — (ZIP) [file pone.0330765.s004.zip › Synthetic dataset/Result/AB/Pre/Figure1-3/Figure 2025-01-11 104418 (3).png]

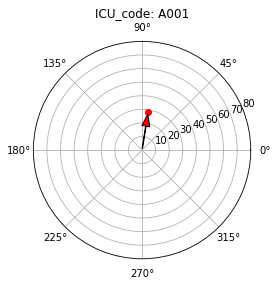

Supplement: S4 File — (ZIP) [file pone.0330765.s004.zip › Synthetic dataset/Result/AB/Pre/Figure1-3/Figure 2025-01-11 104418 (4).png]

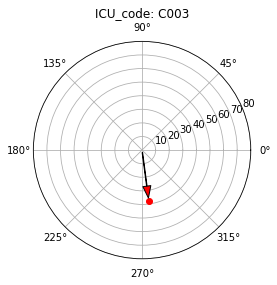

Supplement: S4 File — (ZIP) [file pone.0330765.s004.zip › Synthetic dataset/Result/AB/Pre/Figure1-3/Figure 2025-01-11 104418 (5).png]

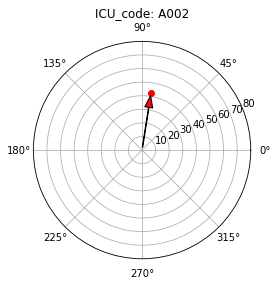

Supplement: S4 File — (ZIP) [file pone.0330765.s004.zip › Synthetic dataset/Result/AB/Pre/Figure1-3/Figure 2025-01-11 104418 (6).png]

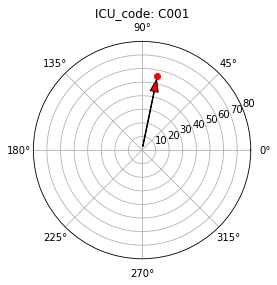

Supplement: S4 File — (ZIP) [file pone.0330765.s004.zip › Synthetic dataset/Result/AB/Pre/Figure1-3/Figure 2025-01-11 104418 (7).png]

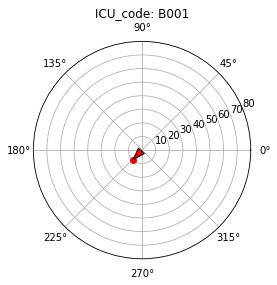

Supplement: S4 File — (ZIP) [file pone.0330765.s004.zip › Synthetic dataset/Result/AB/Pre/Figure1-3/Figure 2025-01-11 104418 (8).png]

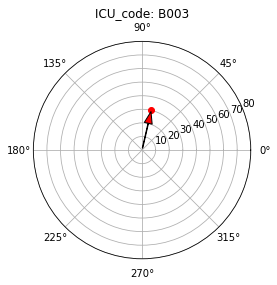

Supplement: S4 File — (ZIP) [file pone.0330765.s004.zip › Synthetic dataset/Result/AB/Pre/Figure1-3/Figure 2025-01-11 104418 (9).png]

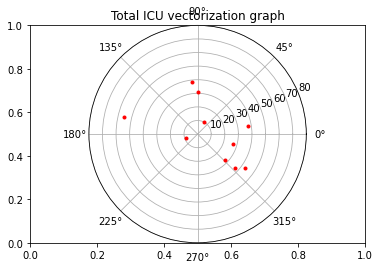

Supplement: S4 File — (ZIP) [file pone.0330765.s004.zip › Synthetic dataset/Result/AB/Pre/Figure4/Figure 2025-01-11 104804 (0).png]

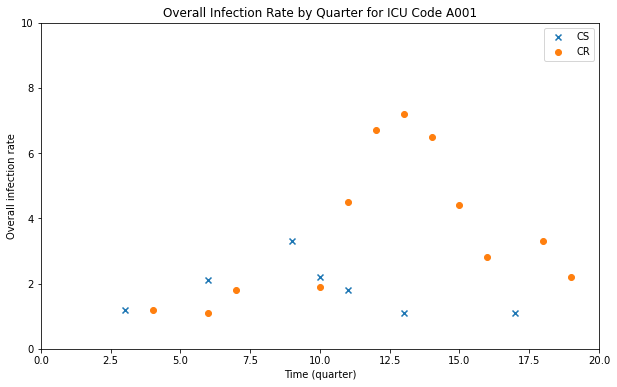

Supplement: S4 File — (ZIP) [file pone.0330765.s004.zip › Synthetic dataset/Result/KP/Post/Figure1-1/Figure 2025-01-08 223117 (0).png]

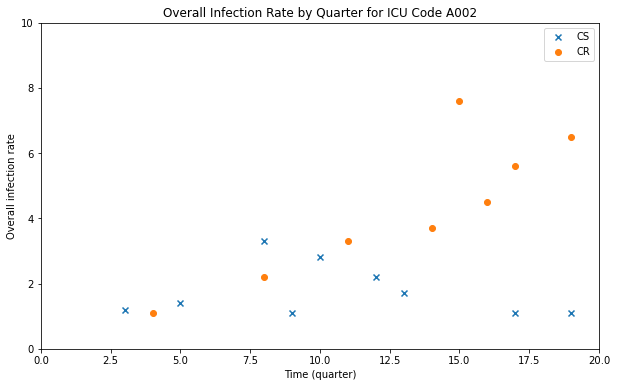

Supplement: S4 File — (ZIP) [file pone.0330765.s004.zip › Synthetic dataset/Result/KP/Post/Figure1-1/Figure 2025-01-08 223117 (1).png]

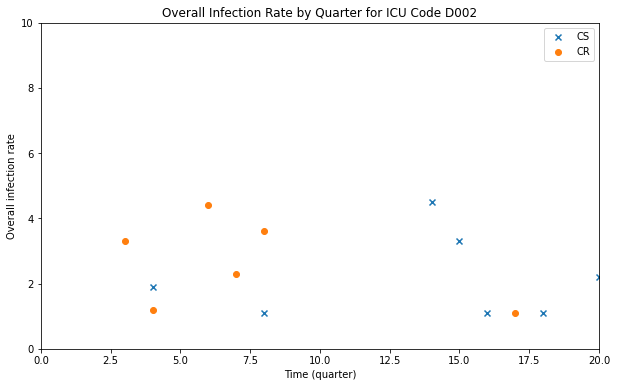

Supplement: S4 File — (ZIP) [file pone.0330765.s004.zip › Synthetic dataset/Result/KP/Post/Figure1-1/Figure 2025-01-08 223117 (10).png]

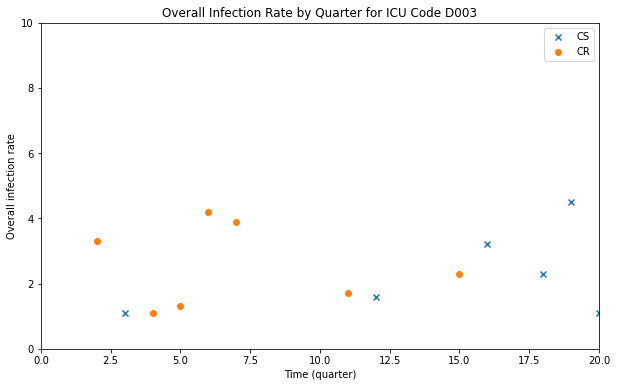

Supplement: S4 File — (ZIP) [file pone.0330765.s004.zip › Synthetic dataset/Result/KP/Post/Figure1-1/Figure 2025-01-08 223117 (11).png]

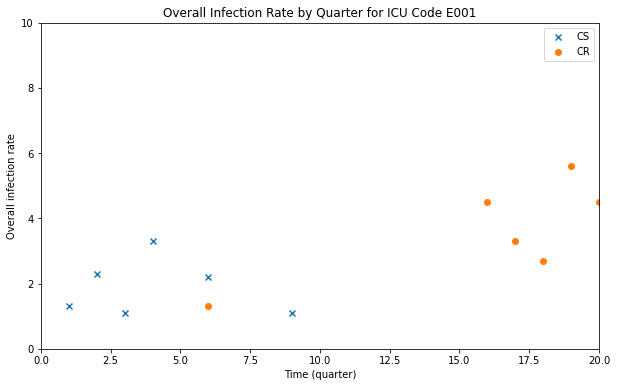

Supplement: S4 File — (ZIP) [file pone.0330765.s004.zip › Synthetic dataset/Result/KP/Post/Figure1-1/Figure 2025-01-08 223117 (12).png]

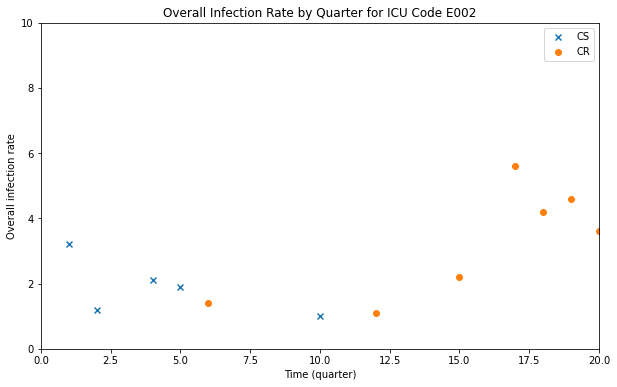

Supplement: S4 File — (ZIP) [file pone.0330765.s004.zip › Synthetic dataset/Result/KP/Post/Figure1-1/Figure 2025-01-08 223117 (13).png]

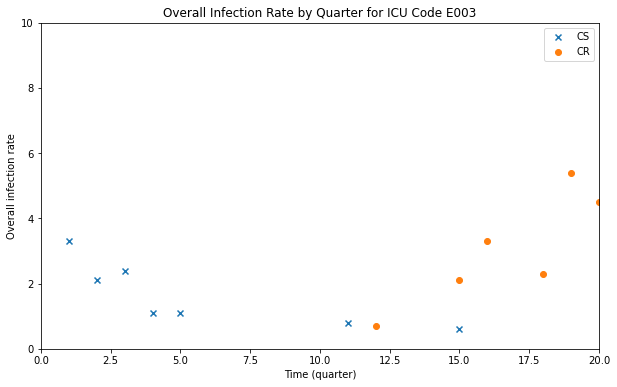

Supplement: S4 File — (ZIP) [file pone.0330765.s004.zip › Synthetic dataset/Result/KP/Post/Figure1-1/Figure 2025-01-08 223117 (14).png]

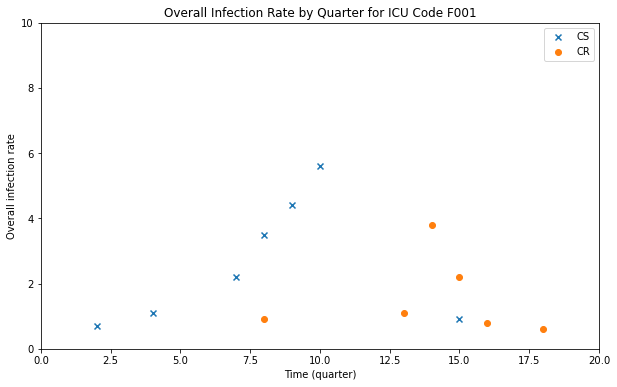

Supplement: S4 File — (ZIP) [file pone.0330765.s004.zip › Synthetic dataset/Result/KP/Post/Figure1-1/Figure 2025-01-08 223117 (15).png]
