# Supplementary material for: Visualization of multidrug-resistant bacterial infection trends in the intensive care units
Source: PLoS One. 2025 Aug 28;20(8):e0330765. doi: 10.1371/journal.pone.0330765 (PMC12393710; doi:10.1371/journal.pone.0330765)
Supplement: Attachment — Submitted filename: Response to Reviewers.doc [file pone.0330765.s005.doc]

6 August 2024

Dwij Raj Bhatta, PhD

Academic Editor

PLOS ONE

Re: PONE-D-24-03240,

“Visualization of multidrug-resistant bacterial infection trends in the intensive care units”

**Dr. Dwij Raj Bhatta:**

On behalf of the authors, I would like to thank you and the reviewers for helpful comments on our submitted manuscript, “Visualization of multidrug-resistant bacterial infection trends in the intensive care units”. We have carefully reviewed the comments and have revised the manuscript accordingly.

**Reviewer #1’s comments:**

**MAJOR COMMENTS**

**Q1. The main concern on the study, as also stated by the Authors, is the lack of a validation of the method for the graphical representation of the data. Authors defined this method as “new infection control indicator” but no evidence was provided.**

**Response:**

We agree with the reviewer’s comment. Our study aimed to develop the new indicators for visualizing the trends of multidrug-resistant bacterial infection monitored by KONIS. Although this new method was developed out of practical necessity, further validation studies and the judgment of readers will be necessary to fully evaluate its usefulness. We have clearly described this limitation in the revised manuscript as follows:

**In line 54 (abstract) of the revised manuscript,**

**Original:** Graph transformation allowed the observation of the multidrug-resistant bacterial infection trends in ICUs. Arrow categories are useful in infection control and identifying factors for reducing infections.

**Revised:** Graph transformation allowed the observation of MDR bacterial infection trends in ICUs. Further studies should aim to confirm whether our arrow indicators are useful for infection control and in identifying factors for reducing infections.

**In line 328 of the revised manuscript,**

**Original:** If validated in subsequent studies through a comparison with existing methods, the arrows and arrow categories presented in this study may be used as infection control indicators. Furthermore, it may be possible to compare the characteristics of the ICUs in each category to determine the key factors involved in the reduction of resistant bacteria.

**Revised:** Existing data was transformed through a series of processes to visualize it in a single graph, making it easier to understand the situation across hundreds of ICUs. Although the method employed in this study may seem unfamiliar due to its novelty, potential validation through future comparisons with conventional methods could position it as a new infection control indicator. However, to fully evaluate its usefulness, further validation studies and the judgment of readers will be necessary.

**Q2. The Authors described the following graphical representation method: “An arrow was drawn with the average of the CS bacterial infection rate as the starting point and that of the CR bacterial infection rate as the endpoint. Then, the length and angle of the arrow were measured. In this process, the overall infection rate was scaled up 20 times”. However, not enough clear reasons for such a representation, and, particularly, mathematical basis for that were provided. How can readers understand the elements of representation and not see them as an arbitrary choice of the authors? What mathematical models underlie the graphic representation?**

**Response:**

Thank you for the careful comments. From a macroscopic perspective, we hypothesized that there is a physical force that transforms the susceptible bacterial population into a resistant bacterial population. We believed this force reflects various factors, including antimicrobial stewardship and infection control. Since vectors are commonly used to represent force in physics, we applied this concept in our study. In physics, when a force acts on an object, it can be considered to act on the center of mass of that object. Similarly, we considered the force to act on the distribution centers of the susceptible and resistant bacterial populations. To create the vectors, we performed the following process. First, the overall rate of infections (pneumonia, UTI, and BSI) that occurred during the observation period in each ICU was plotted according to the pathogen involved (AB, KP, and PA). These rates were then delineated according to the carbapenem susceptibility status (Fig 1. A, D, and G). The overall infection rate was calculated as follows: number of infections (pneumonia, UTI, or BSI) / total patient–days × 1000. Using the overall infection rate graph, the average CS and CR bacterial infection rates during the observation period were plotted together with the error range for each ICU (Fig 1. B, E, and H). An arrow was drawn with the average of the CS bacterial infection rate as the starting point and that of the CR bacterial infection rate as the endpoint. Then, the length and angle of the arrow were measured. In this process, the overall infection rate was scaled up 20 times for the optimal readability. This approach was not based on a specific mathematical model but rather utilized general concepts from physics. We implemented scale-up at magnitudes of 10, 20, 50, and 100. For example, as the scale-up magnitude increases, the absolute values of the slopes of all data also increase, causing the data to cluster closer to a vertical line, which complicates visual interpretation (Figure S1 D, H, and L, below). And if a small scale-up magnitude is selected, all data points are clustered closer to a horizontal line (Figure S1, A, E, and I, below). However, since the quadrant to which each data point belongs does not change with different scale-up magnitudes, selecting an appropriate scale-up value for visual interpretation is manageable. Maintaining a consistent scale-up value across comparisons and observations should be feasible.

**Figure S1. Data distribution tendency according to scale-up factor**

**A**. Randomized data group 1 with scale-up magnitude 10. **B**. Randomized data group 1 with scale-up magnitude 20. **C**. Randomized data group 1 with scale-up magnitude 50. **D**. Randomized data group 1 with scale-up magnitude 100. **E**. Randomized data group 2 with scale-up magnitude 10. **F**. Randomized data group 2 with scale-up magnitude 20. **G**. Randomized data group 2 with scale-up magnitude 50. **H**. Randomized data group 2 with scale-up magnitude 100. **I**. Randomized data group 3 with scale-up magnitude 10. **J**. Randomized data group 3 with scale-up magnitude 20. **K**. Randomized data group 3 with scale-up magnitude 50. **L**. Randomized data group 3 with scale-up magnitude 100.

We have revised Figure 2 for better reader understanding. The process to create the vectors is clarified as follows:

**In line 134 of the revised manuscript,**

**Original:**

“First, the overall rate of infections (pneumonia, UTI, and BSI) that occurred during the observation period in each ICU was plotted according to the pathogen involved (AB, KP, and PA).”

**Revised:**

From a macroscopic perspective, it was hypothesized that a physical force exists which transforms the susceptible bacterial population into a resistant bacterial population. This force was believed to reflect various factors, including antimicrobial stewardship and infection control. Since vectors are commonly used to represent force in physics, this concept was applied in the study. In physics, when a force acts on an object, it is considered to act on the center of mass of that object. Similarly, the force was considered to act on the distribution centers of the susceptible and resistant bacterial populations. To create the vectors, we performed the following process. First, the overall rate of infections (pneumonia, UTI, and BSI) that occurred during the observation period in each ICU was plotted according to the pathogen involved (AB, KP, and PA).

**In line 149 of the revised manuscript,**

**Original:**

In this process, the overall infection rate was scaled up 20 times.

**Revised:**

In this process, the overall infection rate was scaled up 20 times for the optimal readability (supplementary material #1).

**In line 158 of the revised manuscript,**

**Original:** The length of the arrow correlates with the speed of transition from antibiotic-susceptible bacteria to antibiotic-resistant bacteria. A shorter length indicates a rapid transition, while a longer length indicates a slow transition (Fig 2 D). The angle of the arrow angle signifies changes in the overall infection rate. A large positive angle denoted a significant increase in the overall infection rate, while a small positive angle signified a mild increase in the overall infection rate. A negative angle indicated a decrease in the overall infection rate (Fig 2 E).

**Revised:** A left-sided arrow indicates a change from resistance to susceptibility, whereas a right-sided arrow indicates a change from susceptibility to resistance. The length of the arrow correlates with the speed of transition from antibiotic-susceptible bacteria to antibiotic-resistant bacteria. A shorter length indicates a rapid transition, while a longer length indicates a slow transition (Fig 2A). An upward arrow indicates an increase in the overall infection rate, while a downward arrow indicates a decrease. The closer the angle of the arrow is to 90°, the greater the increase in overall infection rates. Conversely, the closer the angle of the arrow is to 270°, the greater the decrease in overall infection rates. For example, an arrow with an angle of 60° indicates a greater increase in the overall infection rate than an arrow with an angle of 30° (Fig 2B).

**Figure 2.** Angle and length of arrow and ICU category.

**Q3. Statistical method: Python script should be provided to better understand the study design and method for calculation.**

**Response:**

We agree with the reviewer comment. In this paper, we primarily used Python and Microsoft Excel. According to reviewer's suggestion, we have provided the Python and Excel scripts in the supplementary materials of revised manuscript.

**In line 192 of the revised manuscript,**

**Original:**

Python was used for the overall data extraction process, classification, and graph drawing. Tables and some graphs were created using Microsoft Excel and SPSS (Student’s t-test, Mann-Whitney test, chi-square test, Fisher’s exact test). And some figures were created using Microsoft Power point.

**Revised:**

Python and Microsoft Excel were used for the overall data extraction process, classification, and graph drawing (supplementary material #2). Tables and some graphs were created using Microsoft Excel, Microsoft Power point and SPSS (Student’s t-test, Mann-Whitney test, chi-square test, Fisher’s exact test).

**Q4. Criteria for stratification in red, yellow and green groups should be provided.**

**Response:**

Thank you for valuable comments. The criteria for dividing the categories (red, yellow, green) are the angle and length of the vectors. In this paper, the length of the vector represents the transition speed from the susceptible bacterial population to the resistant bacterial population from a macroscopic perspective. A shorter length indicates a faster transition, while a longer length indicates a slower transition. If we consider it in terms of force, a shorter length implies a greater force, and a longer length implies a lesser force. Although this is contrary to the common representation in physics where a greater force is depicted by a longer vector, we chose not to make any additional transformations because considering length of vector as the distance between the two populations was easier to understand than taking the reciprocal. The cutoff length used to divide the categories in this paper was set at 10, which is approximate to the average length of all data. Depending on the characteristics of the data from individual countries or regions, it is necessary to select an appropriate cutoff length. Maintaining a consistent cutoff length value across comparisons and observations should be feasible. In this paper, the angle of the vector represents the change in infection rate during the transition from the susceptible bacterial population to the resistant bacterial population from a macroscopic perspective. A large positive angle indicates a significantly increased infection rate, a small positive angle indicates a mildly increased infection rate, and a negative angle indicates a decreased infection rate. In this paper, we broadly categorized the vectors as follows: an angle of 0-90 degrees represents an increase in the infection rate, an angle of 270-360 degrees represents a decrease in the infection rate, and an angle of 90-270 degrees represents a reverse transition from the resistant bacterial population to the susceptible bacterial population. Combining the meanings of the vector's length and angle, we defined the red category as vectors shorter than the cutoff length with angles between 0-90 degrees, which can be thought of as populations with a fast transition speed from susceptible to resistant bacteria and an increasing infection rate. The yellow category was defined as vectors longer than the cutoff length with angles between 270-360 degrees, representing populations with a slow transition speed and a decreasing infection rate. The green category was defined as vectors with angles between 90-270 degrees, regardless of length, representing populations undergoing a reverse transition from resistant to susceptible bacteria. We have provided these information in abstract and method section of the revised manuscript as follows:

**In line 41 (abstract) of the revised manuscript,**

ICUs are categorized into red (rapid shift from susceptible to resistant bacteria and increased infection rate), yellow (slow shift from susceptible to resistant bacteria and decreased infections), and green (shift from resistant to susceptible bacteria) groups based on bacterial transition and infection rate trends.

**In line 169 of the revised manuscript,**

Based on the angles and lengths of the arrows for each ICU, we have categorized ICUs into red, yellow, and green groups (Fig 2C). The red category (aggressive ICU group) was defined as ICUs where the bacteria underwent a rapid transition from susceptible to resistant strains accompanied by an increase in the infection rate. In the graph, the arrows were notably short (<10), and the angle fell between 0° and 90°. The yellow category (indolent ICU group) was defined as ICUs where the bacteria underwent a slow transition from susceptible to resistant strains accompanied by a simultaneous decrease in infection rates. In the graph, the arrows were notably long (≥10), and the angle fell between 270° and 360°. The cutoff length of transition speed used to define the red and yellow categories was set at 10, which is approximately the average length of all arrows. Lastly, the green category (reverse ICU group) was defined as ICUs where bacteria exhibited a reversed trend, from resistant to susceptible strains. In the graph, the angle of the arrow fell between 90° and 270°, regardless of length.

**Q5. Advantages of the graphical representation of the study compared to traditional methods should be largely discuss.**

**Response:**

Over time, the number of ICUs participating in KONIS has steadily increased, with almost 300 ICUs participating by 2022. By collecting data from these ICUs and analyzing with traditional methods, we can determine the overall infection incidence rate and the proportion of resistant bacteria, as well as observe changes over time. However, there are several inconveniences associated with traditional methods. First, since the overall infection incidence rate and the proportion of resistant bacteria are representative values from all ICUs participating in KONIS rather than from individual ICUs, it is difficult to determine these values for each ICU and assess the current state of infection control. Additionally, analyzing with traditional methods requires plotting graphs for each ICU, reflecting data from as many as 17 years, which is practically very challenging. Second, it is difficult to simultaneously track and understand the situation in over 300 ICUs using traditional methods. It is hard to grasp 300 graphs at a glance, and even more challenging to continually track them. The new method presented in this paper was developed out of practical necessity, as it has become increasingly difficult to understand and track the situation of surveillance targets with the growing number of ICUs participating in KONIS. Although validations for our new indicators are further needed, it may aid in operating surveillance systems like KONIS.

**In line 288 of the revised manuscript,**

**Original:**

Through this process, we were able to easily observe the trends of infections and resistant bacteria in hundreds of ICUs under surveillance. This stands out as a strength of this study. If verified through comparison with conventional methods, it may be used as an indicator for infection control in the future. Furthermore, it may be possible to compare the characteristics of ICUs classified using our method to determine the key factors involved in reducing infections and resistant bacteria in the ICU.

**Revised:**

Through this process, we were able to easily observe the trends of infections and resistant bacteria in hundreds of ICUs under surveillance. This stands out as a strength of this study. Over time, the number of ICUs participating in KONIS has steadily increased, with almost 300 ICUs participating by 2022. Data from these ICUs could be collected and analyzed using traditional methods to determine the overall infection incidence rate and the proportion of resistant bacteria, as well as to observe changes over time. However, several inconveniences are associated with traditional methods. First, since the overall infection incidence rate and the proportion of resistant bacteria are representative values from all ICUs participating in KONIS rather than from individual ICUs, it is difficult to determine these values for each ICU and assess the current state of infection control. Additionally, analyzing with traditional methods requires plotting graphs for each ICU, reflecting data from as many as 17 years, which is practically very challenging. Second, it is difficult to simultaneously track and understand the situation in over 300 ICUs using traditional methods. It is hard to grasp 300 graphs at a glance, and even more challenging to continually track them. The new method presented in this paper was developed out of practical necessity, as it has become increasingly difficult to understand and track the situation of surveillance targets with the growing number of ICUs participating in KONIS. In this paper, existing data was transformed through a series of processes to visualize it in a single graph, making it easier to understand the situation across hundreds of ICUs. But to fully evaluate its usefulness, numerous follow-up studies and the judgment of readers will be necessary.

**Q6. Authors affirmed that such a graphical representation could be useful for “identifying factors for reducing infections” but this is not proven by the study.**

**Response:**

We agree with the reviewer’s comment. Our study aimed to develop the new indicators for visualizing the trends of multidrug-resistant bacterial infection monitored by KONIS. Although this new method was developed out of practical necessity, further validation studies and the judgment of readers will be necessary to fully evaluate its usefulness. We have clearly described this limitation in discussion section as follows:

**In line 52 (abstract) of the revised manuscript,**

**Original:** Graph transformation allowed the observation of the multidrug-resistant bacterial infection trends in ICUs. Arrow categories are useful in infection control and identifying factors for reducing infections.

**Revised:** Graph transformation allowed the observation of MDR bacterial infection trends in ICUs. Further studies should aim to confirm whether our arrow indicators are useful for infection control and in identifying factors for reducing infections.

**Q7. The whole abstract is not enough clear**

**Response:**

Thank you for your valuable comments. For the clarity of the abstract, we have revised manuscript as follows.

**In line 31 of the revised manuscript,**

**Background:** The Korean National Healthcare–associated Infections Surveillance System (KONIS) monitors multidrug–resistant (MDR) bacterial infections in intensive care units (ICUs). However, simultaneously monitoring hundreds of ICUs remains challenging. Our study aimed to visualize the trends of MDR gram–negative bacterial infections in ICUs monitored by KONIS.

**Methods:** We evaluated KONIS data from 137 ICUs (2006 to 2011) and from 368 ICUs (2017 to 2022). Pneumonia, urinary tract infection, and bloodstream infection caused by *Klebsiella pneumoniae*, *Pseudomonas aeruginosa*, and *Acinetobacter baumannii* were analyzed. Graph transformation was employed to convert the infection rate graphs of each ICU into arrows. The length and angle of the arrows reflect changes in carbapenem susceptibility and infection rate, respectively. ICUs are categorized into red (rapid shift from susceptible to resistant bacteria and increased infection rate), yellow (slow shift from susceptible to resistant bacteria and decreased infections rate), and green (shift from resistant to susceptible bacteria) groups. The proportional changes in each ICU category were compared during the first and last five years of the study periods.

**Results:** For *K. pneumoniae*, the proportion of red category ICUs increased (0% to 17%), while the proportions of yellow (33.3% to 7%) and green category ICUs (66.6% to 36%) decreased. For *P. aeruginosa*, the proportions of red (12% to 27%) and green category ICUs (38% to 46%) increased, while the proportion of yellow category ICUs decreased (8% to 2%). For *A. baumannii*, the proportions of red (19% to 14%) and yellow category ICUs (5% to 1%) decreased, while the proportion of green category ICUs increased (19% to 72%).

**Conclusions:** Graph transformation allowed the observation of MDR Gram-negative bacterial infection trends in ICUs. Further studies should aim to confirm whether our arrow indicators are useful for infection control and in identifying factors for reducing infections.

**Q8. Abstract, Results: reasons for the comparison between the first 5 years of observation and following 5-years are not understood (line 33)**

**Response:**

We appreciate your attention to this matter. Comparing only the first 5 years and the last 5 years in the statistical analysis of the overall 17-year infection trends can be due to two reasons. First, the first and last periods may provide more consistent and comparable data sets because, in the middle periods, there were some changes in KONIS guidelines aimed at improving data collection during the 17-year study period. Second, analyzing the entire 17-year period in detail can be statically complex and time-consuming. Focusing on the first and last five years simplifies the analysis and makes it more manageable. Limited resources and time may necessitate focusing on the most impactful periods rather than conducting a detailed analysis of every year.

**In line 120 of the revised manuscript,**

**Original:**

Among the ICUs that participated in the KONIS from the 3rd quarter of 2006 to the 2nd quarter of 2022, those who participated in the first 5 years (3rd quarter of 2006 to 2nd quarter of 2011) and those who participated in the last 5 years (3rd quarter of 2017 to 2nd quarter of 2022) were selected.

**Revised:**

Among the ICUs that participated in KONIS from 2006 to 2022, we selected those that participated in the first five years (2006 to 2011) and those that participated in the last five years (2017 to 2022). KONIS has collected and analyzed data on a quarterly basis according to its own guidelines. The first and last periods may provide more consistent and comparable data sets because, in the middle periods, there were some changes in KONIS guidelines aimed at improving data collection.

**Q9. Introduction line 69: “Furthermore…”. This affirmation should be demonstrated.**

**Response:**

We agree with the reviewer. We have deleted this sentence in the introduction section.

**Q10. Line79: therefore, is it a prevalence study? And why the Authors have chosen to include only infections “that occurred on the third day after ICU admission”?**

**Response:**

Thank you for calling attention to our error. “on third day” was typo-error, it should be read as “≥3 days after ICU admission.” Because the risk of HAI is generally low in short-term inpatients, patients who had been hospitalized for less than 3 days were excluded in KONIS in South Korea and NHSN in United States. For the clarity, we have revised as follows:

**In line 96 of the revised manuscript,**

**Original:**

The diseases analyzed were pneumonia, urinary tract infection (UTI), and bloodstream infection (BSI) that occurred on the third day after ICU admission.

**Revised:**

The diseases analyzed were pneumonia, urinary tract infection (UTI), and bloodstream infection (BSI). Because the risk of healthcare-associated infection is generally low in short-term inpatients, patients who had been hospitalized for less than 3 days were excluded [14].

**Q11. CRBSI were not included into the study? Why not?**

**Response:**

In the paper, CRBSI was included in BSI for data analysis. Similarly, CAUTI was included in UTI, and VAP was included in pneumonia for the analysis. This information was included in Table 2 as follows:

**Table 2. The rates of urinary tract infection, bloodstream infection, and pneumonia in** ICUs

|  | *A. baumannii* | |
| --- | --- | --- |
| Variable | First 5 years  (2006–2011) | Last 5 year  (2017–2022) |
| **Urinary tract infection** |  |  |
| **UTI rate** | 4.12 (2.68–4.82) | 1.23 (0.73–1.97) |
| **Catheter–associated UTI rate** | 4.66 (3.13–5.98) | 1.22 (0.72–1.90) |
| Urinary catheter utilization ratio | 0.88 (0.79–0.94) | 0.89 (0.85–0.92) |
| Number of urinary catheter–days | 1164.76 (868.86–1865.40) | 1181.80 (955.47–1492.17) |
| **Bloodstream infection** |  |  |
| **BSI rate** | 1.96 (1.42–2.59) | 1.76 (1.19–2.89) |
| **Central line–associated BSI rate** | 3.40 (2.21–4.30) | 1.48 (1.01–2.59) |
| Central line utilization ratio | 0.51 (0.43–0.66) | 0.57 (0.48–0.68) |
| Number of central line–days | 895.00 (738.31–1192.40) | 784.10 (581.12–1013.56) |
| **Pneumonia** |  |  |
| **Pneumonia rate** | 1.39 (0.87–1.88) | 0.70 (0.34–1.15) |
| **Ventilator–associated pneumonia rate** | 2.20 (1.39–4.05) | 0.34 (0.12–0.61) |
| Ventilator utilization ratio | 0.36 (0.28–0.48) | 0.37 (0.30–0.50) |
| Number of ventilator–days | 463.50 (364.17–929.50) | 546.88 (346.52–831.04) |

**Q12. Line 108: “ICUs where both CS and CR bacterial infections had occurred for more than two-quarters were finally selected.” Explain why.**

**Response:**

Thank you for the important comments. “two-quarters” should be read as “two quarters”. ICUs where both CS and CR bacterial infections had occurred for more than two quarters were finally selected to calculate the change of bacterial susceptibility. In the graph of infection rate over time, calculating the distribution center of the susceptible bacterial population, which is used as the starting point of the vector, requires at least two independent points. Since the x-axis represents time (quarters), the infection rate caused by susceptible bacteria must be available for at least two or more time points to determine the distribution center of the susceptible bacterial population. Similarly, to calculate the distribution center of the resistant bacterial population, which is used as the endpoint of the vector, the infection rates caused by resistant bacteria must be available for at least two or more quarters.

**In line 129 of the revised manuscript,**

**Original:**

ICUs where both CS and CR bacterial infections had occurred for more than two-quarters were finally selected.

**Revised:**

ICUs where both CS and CR bacterial infections had occurred for more than two quarters were finally selected to calculate the change of bacterial susceptibility.

**Q13. Line 102: The Authors stated: “Among the ICUs that participated in the KONIS from the 3rd quarter of 2006 to the 2nd quarter of 2022, those who participated in the first 5 years (3rd quarter of 2006 to 2nd quarter of 2011) and those who participated in the last 5 years (3rd quarter of 2017 to 2nd quarter of 2022) were selected”. Does it mean that two different groups of ICUs were selected? If so, Authors should clarify reasons and criteria for this selection method. Otherwise a clearer description of population stratification should be provided.**

**Response:**

Thank you for your valuable comments. During the study period, the number of ICUs participating in KONIS steadily increased from 137 (between 2006 and 2017) to 368 (between 2017 and 2022). As mentioned above the first and last periods may provide more consistent and comparable data sets because, during the middle periods, there were changes in KONIS guidelines aimed at improving data collection. Because we did not compare each ICU between the two study periods, all ICUs that met the inclusion criteria were included for each study period. Unlike previous studies, we did not evaluate the change in infection rates and MDR rates. Instead, we evaluated the infection control status of the ICUs. For example, the proportions of red (19% to 14%) and yellow category (5% to 1%) ICUs for *A. baumannii* decreased, while the proportion of green category ICUs increased (19% to 72%). This means that more ICUs in KONIS are effectively controlling drug-resistant *A. baumannii* in recent years. Therefore, including different numbers of ICUs rather than the same numbers of paired ICUs provided a real status of ICUs participating in KONIS. We have revised the manuscript as follows.

**In line 120 of the revised manuscript,**

**Original:**

Among the ICUs that participated in the KONIS from the 3rd quarter of 2006 to the 2nd quarter of 2022, those who participated in the first 5 years (3rd quarter of 2006 to 2nd quarter of 2011) and those who participated in the last 5 years (3rd quarter of 2017 to 2nd quarter of 2022) were selected.

**Revised:**

Among the ICUs that participated in KONIS from 2006 to 2022, we selected those that participated in the first five years (2006 to 2011) and those that participated in the last five years (2017 to 2022). KONIS has collected and analyzed data on a quarterly basis according to its own guidelines. The first and last periods may provide more consistent and comparable data sets because, in the middle periods, there were some changes in KONIS guidelines aimed at improving data collection.

**Reviewer #2’s comments:**

**Q1. One of the most important bacterial nosocomial bacterial pathogen *Staphylococcus aureus* (MRSA) has not been included in this study. Details of MRSA may be included if data is available.**

**Response:**

We appreciate your attention to this matter. MRSA has decreased both in South Korea and worldwide (Bae et al., J Korean Med Sci. 2023; 38, e73; Diekema et al., Open Forum Infect Dis. 2019; 6: S47-S53). A recent study evaluated the changing epidemiology of pathogenic bacteria over the past 20 years in Korea (Bae et al., J Korean Med Sci. 2023; 38, e73). The prevalence of *S. aureus* was the highest (13.1%) until 2012 but decreased to 10.3% in 2016, consistent with the decrease in oxacillin resistance from 76.1% in 2008 to 62.5% in 2016. In contrast, the cefotaxime resistance of *E. coli* increased from 9.0% in 1997 to 34.2% in 2016. The resistance rates in *P. aeruginosa* and *A. baumannii* increased from 13.9% to 30.8% and 0.7% to 73.5%, respectively, during the study period. These data highlight the importance of KONIS, especially in MDR gram-negative bacteria. Therefore, our study aimed to visualize the trends of MDR gram–negative bacterial infections monitored by KONIS. We have clarified the focus of this study as follows:

**In line 31 (abstract) of the revised manuscript,**

**Original:** Korean National Healthcare-associated Infections Surveillance System (KONIS) monitors multidrug-resistant bacterial infections in intensive care units (ICUs). However, the simultaneous monitoring of the hundreds of ICUs remains challenging.

**Revise:** The Korean National Healthcare–associated Infections Surveillance System (KONIS) monitors multidrug–resistant (MDR) bacterial infections in intensive care units (ICUs). However, simultaneously monitoring hundreds of ICUs remains challenging. Our study aimed to visualize the trends of MDR gram–negative bacterial infections monitored by KONIS.

**In line 52 of the revised manuscript,**

**Original:** Graph transformation allowed the observation of the multidrug-resistant bacterial infection trends in ICUs. Arrow categories are useful in infection control and identifying factors for reducing infections.

**Revise:** Graph transformation allowed the observation of MDR Gram-negative bacterial infection trends in ICUs. Further studies should aim to confirm whether our arrow indicators are useful for infection control and in identifying factors for reducing infections.

**In line 72 of the revised manuscript,**

**Original:** The KONIS is used to monitor trends in specific site and device–related infections and resistant bacteria in the ICU.

**Revise:** The KONIS is used to monitor trends in specific site and device–related infections and resistant bacteria in the ICU. In recent years, the burden of MDR Gram–negative bacterial infections in South Korea have increased significantly, highlighting the growing importance of the KONIS system [11].

**Q2. Duration of each quarter of the study is not clearly mentioned and difficult to understand.**

**Response:**

Thank you for the important comments. KONIS have collected and have analyzed the data on a quarterly basis according to its own guidelines. This is a common approach to obtain more stable and meaningful statistical results. It is particularly useful when the incidence rates are low, making it difficult to analyze on a monthly basis. This method helps reduce data variability and allows for clearer identification of trends. The year is divided into four quarters: the first quarter from January to March, the second quarter from April to June, the third quarter from July to September, and the fourth quarter from October to December. However, a quarterly-based year may confuse readers. Therefore, we have described the study period to a yearly basis in body of text to avoid readers’ confusion.

During the study period, the number of ICUs participating in KONIS steadily increased from 137 (between 2006 and 2017) to 368 (between 2017 and 2022). Comparing only the first 5 years and the last 5 years in the statistical analysis of the overall 17-year infection trends can be due to two reasons. First, the first and last periods may provide more consistent and comparable data sets because, in the middle periods, there were some changes in KONIS guidelines aimed at improving data collection during the 17-year study period. Second, analyzing the entire 17-year period in detail can be statically complex and time-consuming. Focusing on the first and last five years simplifies the analysis and makes it more manageable. Limited resources and time may necessitate focusing on the most impactful periods rather than conducting a detailed analysis of every year.

Because we did not compare each ICU between the two study periods, all ICUs that met the inclusion criteria were included for each study period. Unlike previous studies, we did not evaluate the change in infection rates and MDR rates. Instead, we evaluated the infection control status of the ICUs. For example, the proportions of red (19% to 14%) and yellow category (5% to 1%) ICUs for *A. baumannii* decreased, while the proportion of green category ICUs increased (19% to 72%). This means that more ICUs in KONIS are effectively controlling drug-resistant *A. baumannii* in recent years. Therefore, including different numbers of ICUs rather than the same numbers of paired ICUs provided a real status of ICUs participating in KONIS. For the clarity of the manuscript, we have revised it as follows:

**In line 120 of the revised manuscript,**

**Original:**

Among the ICUs that participated in the KONIS from the 3rd quarter of 2006 to the 2nd quarter of 2022, those who participated in the first 5 years (3rd quarter of 2006 to 2nd quarter of 2011) and those who participated in the last 5 years (3rd quarter of 2017 to 2nd quarter of 2022) were selected.

**Revised:**

Among the ICUs that participated in KONIS from 2006 to 2022, we selected those that participated in the first five years (2006 to 2011) and those that participated in the last five years (2017 to 2022). KONIS has collected and analyzed data on a quarterly basis according to its own guidelines. The first and last periods may provide more consistent and comparable data sets because, in the middle periods, there were some changes in KONIS guidelines aimed at improving data collection.

**In column heading of the tables 1 and 2,**

**Q3. Methods of detection of carbapenem resistance was not included in the methodology section.**

**Response:**

The susceptibility of bacteria to carbapenems was determined according to the guidelines of the Clinical and Laboratory Standards Institute (CLSI). During the study period, the susceptibility breakpoints for imipenem, meropenem, and doripenem in *K. pneumoniae* were revised from ≤4 to ≤1 mg/L in 2010. For *P. aeruginosa*, the susceptibility breakpoints were revised from ≤4 to ≤2 mg/L in 2012. Similarly, the susceptibility breakpoints for *A. baumannii* were revised from ≤4 to ≤2 mg/L in 2014.

**In line 89 of the revised manuscript,**

**Original:**

The bacterial infections were further stratified into carbapenem-susceptible (CS) and carbapenem-resistant (CR) types based on carbapenem susceptibility.

**Revised:**

The bacterial infections were further stratified into carbapenem–susceptible (CS) and carbapenem–resistant (CR) types based on carbapenem susceptibility. The susceptibility of bacteria to carbapenems was determined according to the guidelines of the Clinical and Laboratory Standards Institute (CLSI) [12]. During the study period, the susceptibility breakpoints for imipenem, meropenem, and doripenem in *K. pneumoniae* were revised from ≤4 to ≤1 mg/L in 2010. For *P. aeruginosa*, the susceptibility breakpoints were revised from ≤4 to ≤2 mg/L in 2012. Similarly, the susceptibility breakpoints for *A. baumannii* were revised from ≤4 to ≤2 mg/L in 2014 [12,13].

**Q4. Information provided in the figures is difficult to understand.**

**Response:**

Thank you for valuable comments. For better readability, the figure legends have been revised as follows.

**In line 413 of the revised manuscript,**

**Figure 2.** Angle and length of arrow and ICU category. **A:** A left-sided arrow indicates a change from resistance to susceptibility, whereas a right-sided arrow indicates a change from susceptibility to resistance. The length of the arrow correlates with the speed of transition from antibiotic-susceptible bacteria to antibiotic-resistant bacteria. **B:** An upward arrow indicates an increase in the overall infection rate, while a downward arrow indicates a decrease. **C:** ICUs are categorized into red (rapid shift from susceptible to resistant bacteria and increased infection rate), yellow (slow shift from susceptible to resistant bacteria and decreased infections rate), and green (shift from resistant to susceptible bacteria) groups.

**In line 424 of the revised manuscript,**

**Figure 4.** Distribution of ICUs. The endpoints of the arrows for individual ICUs are represented as points in polar coordinates. All starting points of arrows have been moved to the origin. **A:** *K. pneumoniae* infection during the first 5 years. **B:** *K. pneumoniae* infection during the last 5 years: **C:** *P. aerugionosa* infection during the first 5 years. **D:** *P. aerugionosa* infection during the last 5 years. **E:** *A. baumannii* infection during the first 5 years (total 43 ICUs). **F:** *A. baumannii* infection during the last 5 years.

**In line 430 of the revised manuscript,**

**Figure 5.** Arrow category trend during the first and last 5 years of the KONIS. **A:** *K. pneumoniae* infection during the first 5 years. **B:** *K. pneumoniae* infection during the last 5 years: **C:** *P. aerugionosa* infection during the first 5 years. **D:** *P. aerugionosa* infection during the last 5 years. **E:** *A. baumannii* infection during the first 5 years (total 43 ICUs). **F:** *A. baumannii* infection during the last 5 years.

We have revised our paper as described above and we believe we have addressed all questions and comment, but would be happy to provide further information or revision if necessary. Thank you for your consideration.

Sincerely yours,

Mi Suk Lee, MD, PhD

Professor,

Division of Infectious Diseases,

Department of Internal Medicine,

Kyung Hee University College of Medicine,

Kyung Hee University Hospital,

23, Kyung Hee Dae-ro, Dongdaemungu, Seoul, 02447,

Republic of Korea,

Phone: (82) 2 9588159, Fax number: (82) 2 9681848,

E-mail: mslee7@gmail.com
