# Supplementary material for: Visualization of multidrug-resistant bacterial infection trends in the intensive care units
Source: PLoS One. 2025 Aug 28;20(8):e0330765. doi: 10.1371/journal.pone.0330765 (PMC12393710; doi:10.1371/journal.pone.0330765)
Supplement: Attachment — Submitted filename: Response to Reviewers.docx [file pone.0330765.s006.docx]

22 May 2025

Ali Amanati

Academic Editor

PLOS ONE

Re: PONE-D-24-03240R1,

“Visualization of multidrug-resistant bacterial infection trends in the intensive care units”

**Dr. Ali Amanati:**

On behalf of the authors, I would like to thank you and the reviewers for helpful comments on our submitted manuscript, “Visualization of multidrug-resistant bacterial infection trends in the intensive care units”. We have carefully reviewed the comments and have revised the manuscript accordingly.

**Editor’s comments:**

**Q1. Request for Simplified Explanations of Figures.**

**Response :**

Thank you for your insightful comment. Based on your suggestion, we have concisely revised the figure description as follows.

**In line 409 of the revised manuscript,**

**Original:**

**Figure 1.** Process of graph transform**. A, D, G:** The overall infection rate (pneumonia, UTI, and BSI) at the individual ICU. **B, E, H:** The distribution center of carbapenem-susceptible (CS) and carbapenem-resistant (CR) bacterial infection rates with an error range at the individual ICU. **C, F, I:** Arrows with the distribution center of CS bacterial infection rate as the starting point and that of the CR bacterial infection rate as the endpoint at the individual ICU.

**Figure 2.** Angle and length of arrow and ICU category. **A:** A left-sided arrow indicates a change from resistance to susceptibility, whereas a right-sided arrow indicates a change from susceptibility to resistance. The length of the arrow correlates with the speed of transition from antibiotic-susceptible bacteria to antibiotic-resistant bacteria. **B:** An upward arrow indicates an increase in the overall infection rate, while a downward arrow indicates a decrease. **C:** ICUs are categorized into red (rapid shift from susceptible to resistant bacteria and increased infection rate), yellow (slow shift from susceptible to resistant bacteria and decreased infections rate), and green (shift from resistant to susceptible bacteria) groups.

**Figure 3.** Flowchart of the enrollment of the study ICUs in this Study. BSI = bloodstream infection, ICU = intensive care unit, KONIS = Korean National Healthcare–associated Infections Surveillance System, UTI = urinary tract infection.

**Figure 4.** Distribution of ICUs. The endpoints of the arrows for individual ICUs are represented as points in polar coordinates. All starting points of arrows have been moved to the origin. **A:** *K. pneumoniae* infection during the first 5 years. **B:** *K. pneumoniae* infection during the last 5 years: **C:** *P. aerugionosa* infection during the first 5 years. **D:** *P. aerugionosa* infection during the last 5 years. **E:** *A. baumannii* infection during the first 5 years (total 43 ICUs). **F:** *A. baumannii* infection during the last 5 years.

**Figure 5.** Arrow category trend during the first and last 5 years of the KONIS. **A:** *K. pneumoniae* infection during the first 5 years. **B:** *K. pneumoniae* infection during the last 5 years: **C:** *P. aerugionosa* infection during the first 5 years. **D:** *P. aerugionosa* infection during the last 5 years. **E:** *A. baumannii* infection during the first 5 years (total 43 ICUs). **F:** *A. baumannii* infection during the last 5 years.

**Revised:**

**Figure 1.** Process of graph transform**.**

**A, D, G:** The overall infection rate (pneumonia, UTI, and BSI) at the individual ICU.

**B, E, H:** The distribution center of carbapenem-susceptible (CS) and carbapenem-resistant (CR) bacterial infection rates with an error range at the individual ICU.

**C, F, I:** Arrows showing the transition from CS to CR infection rate distribution centers in individual ICUs.

**Figure 2.** Angle and length of arrow and ICU category.

**A:** A left-sided arrow indicates a shift from resistance to susceptibility, whereas a right-sided arrow indicates a change from susceptibility to resistance. The length of the arrow inversely correlates with the transition speed from antibiotic-susceptible bacteria to antibiotic-resistant bacteria.

**B:** An upward arrow indicates an increase in the overall infection rate, while a downward arrow indicates a decrease.

**C:** ICUs are categorized into red (rapid shift from susceptible to resistant bacteria and increased infection rate), yellow (slow shift from susceptible to resistant bacteria and decreased infections rate), and green (shift from resistant to susceptible bacteria) groups.

**Figure 3.** Flowchart of the enrollment in this study.

BSI = bloodstream infection, ICU = intensive care unit, KONIS = Korean National Healthcare–associated Infections Surveillance System, UTI = urinary tract infection.

**Figure 4.** Distribution of ICUs.

Arrow endpoints for individual ICUs are plotted in polar coordinates, with all starting points shifted to the origin.

**A:** *K. pneumoniae* infection during the first 5 years.

**B:** *K. pneumoniae* infection during the last 5 years:

**C:** *P. aerugionosa* infection during the first 5 years.

**D:** *P. aerugionosa* infection during the last 5 years.

**E:** *A. baumannii* infection during the first 5 years

**F:** *A. baumannii* infection during the last 5 years.

**Figure 5.** Arrow category trend during the first and last 5 years of the KONIS.

**A:** *K. pneumoniae* infection during the first 5 years.

**B:** *K. pneumoniae* infection during the last 5 years:

**C:** *P. aerugionosa* infection during the first 5 years.

**D:** *P. aerugionosa* infection during the last 5 years.

**E:** *A. baumannii* infection during the first 5 years.

**F:** *A. baumannii* infection during the last 5 years.

**Q2. Clarify the Purpose of Each Figure: The authors should explicitly state what each figure represents. They should explain the significance of the data shown and how it relates to the overall findings of the study.**

**Response :**

Thank you for your valuable comments. The purpose of each figure is as follows:

Figure 1: This figure explains the method of representing the overall infection rate graph over time at the individual ICU level by sequentially transforming it into a single vector. Through three examples, it illustrates the three vector categories, which will be discussed later.

Figure 2: This figure explains the meaning of the vector's length and angle. It also aims to categorize the ICUs into three groups based on the vector length and angle.

Figure 4: In this figure, each ICU is represented as a single point, with all monitored ICUs simultaneously displayed on one graph. Vectors are drawn from the origin to each point, and each vector reflects the changes in the distribution of susceptible and resistant bacterial groups, as well as the infection rate, at each individual ICU.

Figure 5: The aim was to present the proportion of ICUs in the red category, which require vigilant monitoring, and the proportion of ICUs in the green category, where infection control is being effectively managed, in a manner that allows for easy comprehension.

**In line 163 of the revised manuscript,**

**Revised:**

Figure 1 explains the method of representing the overall infection rate graph over time at the individual ICU level by sequentially transforming it into a single vector. Through three examples, it illustrates the three vector categories, which will be discussed later.

**In line 193 of the revised manuscript,**

**Revised:**

Figure 2 explains the meaning of the vector's length and angle. It also aims to categorize the ICUs into three groups based on the vector length and angle.

**In line 229 of the revised manuscript,**

**Revised:**

Each ICU is represented as a single point, with all monitored ICUs simultaneously displayed on one graph. Vectors are drawn from the origin to each point, and each vector reflects the changes in the distribution of susceptible and resistant bacterial groups, as well as the infection rate, at each individual ICU.

**In line 245 of the revised manuscript,**

**Revised:**

The aim of figure 5 was to present the proportion of ICUs in the red category, which require vigilant monitoring, and the proportion of ICUs in the green category, where infection control is being effectively managed, in a manner that allows for easy comprehension.

**Q3. Detailed Description of Vectors: The authors must provide a step-by-step explanation of how the vectors are calculated. The specific data points used for calculations. What formulas have been applied to derive the length and angle of the vectors? An example calculation should be added to illustrate the process clearly.**

**Response :**

Thank you for your valuable comments

Step 1. At the ICU level, we categorized a single pathogen based on its carbapenem susceptibility and calculated the overall rate of infections (pneumonia, UTI, and BSI) by time period (year, quarter). The formula used for these calculations is provided below.

Overall infection rate = number of infections (pneumonia, UTI, or BSI) / total patient–days × 1000

Step 2. The central distribution points for each of the carbapenem-susceptible and carbapenem-resistant groups were calculated. The formula used for these calculations is as follows.

x : time (year, quarter)

(The 5-year observation period was divided into a total of 20 quarters, which were sequentially matched in order from 1 to 20.)

average of x = (sum of existed x) / (number of existed x)

y : overall infection rate

average of y = (sum of existed y) / (number of existed y)

Step 3. The length and angle of the vector were calculated using the formula provided below. (In this paper, a scale-up factor of 20 was selected.)

Length of vector =sqrt[(carbapenem-resistant group’s average of x – carbapenem-susceptible group’s average of x)^2^+{(carbapenem-resistant group’s average of y * scale-up factor) – (carbapenem-susceptible group’s average of y * scale-up factor)}^2^]

Angle of vector = (360/2π) * arctan[{(carbapenem-resistant group’s average of y * scale-up factor) – (carbapenem-susceptible group’s average of y * scale-up factor)}/(carbapenem-resistant group’s average of x – carbapenem-susceptible group’s average of x)]

**In line 161 of the revised manuscript,**

**Revised:**

The several equations were used for vector calculation (supplementary material #2).

**Supplementary materials 2**

**Vector calculation**

Step 1. At the ICU level, we categorized a single pathogen based on its carbapenem susceptibility and calculated the overall rate of infections (pneumonia, UTI, and BSI) by time period (year, quarter). The formula used for these calculations is provided below.

Overall infection rate = number of infections (pneumonia, UTI, or BSI) / total patient–days × 1000

Step 2. The central distribution points for each of the carbapenem-susceptible and carbapenem-resistant groups were calculated. The formula used for these calculations is as follows.

x : time (year, quarter)

(The 5-year observation period was divided into a total of 20 quarters, which were sequentially matched in order from 1 to 20.)

average of x = (sum of existed x) / (number of existed x)

y : overall infection rate

average of y = (sum of existed y) / (number of existed y)

Step 3. The length and angle of the vector were calculated using the formula provided below. (In this paper, a scale-up factor of 20 was selected.)

Length of vector =sqrt[(carbapenem-resistant group’s average of x – carbapenem-susceptible group’s average of x)^2^+{(carbapenem-resistant group’s average of y * scale-up factor) – (carbapenem-susceptible group’s average of y * scale-up factor)}^2^]

Angle of vector = (360/2π) * arctan[{(carbapenem-resistant group’s average of y * scale-up factor) – (carbapenem-susceptible group’s average of y * scale-up factor)}/(carbapenem-resistant group’s average of x – carbapenem-susceptible group’s average of x)]

**Reviewer #2’s comments:**

**Q1. This study has incorporated three important nosocomial bacterial pathogens however, one common organism methicillin resistant Staphylococcus aureus (MRSA) has not been included.**

**Response :**

We appreciate your attention to this matter. MRSA has decreased both in South Korea and worldwide (Bae et al., J Korean Med Sci. 2023; 38, e73; Diekema et al., Open Forum Infect Dis. 2019; 6: S47-S53). A recent study evaluated the changing epidemiology of pathogenic bacteria over the past 20 years in Korea (Bae et al., J Korean Med Sci. 2023; 38, e73). The prevalence of *S. aureus* was the highest (13.1%) until 2012 but decreased to 10.3% in 2016, consistent with the decrease in oxacillin resistance from 76.1% in 2008 to 62.5% in 2016. In contrast, the cefotaxime resistance of *E. coli* increased from 9.0% in 1997 to 34.2% in 2016. The resistance rates in *P. aeruginosa* and *A. baumannii* increased from 13.9% to 30.8% and 0.7% to 73.5%, respectively, during the study period. These data highlight the importance of KONIS, especially in MDR gram-negative bacteria. Therefore, our study aimed to visualize the trends of MDR gram–negative bacterial infections monitored by KONIS.

**Reviewer #3’s comments:**

**Q1. It is not clear to the reader whether the overview of infection rates and MDR-rates is meant for a central monitoring institution (here KONIS) or also for the individual ICUs? Will each ICU receive a notification of their standing in the monitoring process, and is this annually, or do they only have access to the country-wide overview? The most logical would be the former, i.e. each ICU is notified with individual and national data, which would benefit benchmarking?**

**Response :**

Thank you for your valuable comments. If the surveillance system proposed in this paper is implemented, it will provide individual ICUs with information on which category—red, yellow, or green—they belong to for each specific pathogen, based on a properly selected monitoring cycle. Additionally, it will provide data on the proportion of ICUs in each category (red, yellow, or green) at the national level. This will offer valuable feedback to individual ICUs in their infection management efforts. Furthermore, from the perspective of operating the surveillance system, by presenting changes in the proportions of red, yellow, and green categories for each specific pathogen, it will serve as a national-level feedback.

**Q2. The figure (fig. 4) with the circles and arrows marked as red spots (they must start in the central point in the circle?) is the most difficult to read and understand, while the figures with colored areas in circle (green, yellow and red) are clearly most informative. Also difficult to read is Figure 1. It is a little confusing that short arrows are worst (rapid change), although the authors try to argue for this version.**

**Response :**

We appreciate your attention to this matter. In the case of figure 1 and 4, which represent intermediate graphs resulting from the vector transformation process, both the ICUs under surveillance and the administrators managing the surveillance system can intuitively reach the same conclusion by figure 5. In practice, when implementing the surveillance system proposed in this paper, only figure 5 will be provided.

**Q3. BSI definition is problematic: Bacteria in blood cannot be present in other infections? What about a K.penumoniae in urine or sputum, and in blood, which is usually the case; will it not be counted as a BSI?**

**Response :**

Thank you for your valuable feedback. The definition of BSI was determined based on the existing KONIS criteria currently in practice. In this study, we conducted vector analysis including BSI, pneumonia, and UTI; therefore, we believe that any potential definition overlap would not have a significant impact on the results.

**Q4. Table 1: Size of ICU beds should be number of ICU beds. Infection nurse is usually called an infection control nurse.**

**Response :**

Thank you for your detailed comments. We have reflected on your feedback and made the following revisions accordingly.

**In line 392 of the revised manuscript,**

**Original:**

**Revised:**

**Q5. Table 2: What do infection rates mean? Per number of patients or bed days or...? How is catheter-utilization or ventilator utilization ratio calculated?**

**Response :**

Thank you for your valuable feedback. The calculation formulas for the points you mentioned are as follows.

(1) infection rate = number of infections / total patient–days × 1000

(2) urinary catheter utilization ratio = number of urinary catheter-days / number of patient-days

(3) ventilator utilization ratio = number of ventilator-days / number of patient-days

(4) central line utilization ratio = number of central line-days / number of patient-days

**In line 403 of the revised manuscript,**

**Original:**

Data are presented as no. (%) or median (interquartile range) unless otherwise indicated.

Abbreviations: BSI = bloodstream infection, ICU = intensive care unit, UTI = urinary tract infection.

**Revised:**

Data are presented as no. (%) or median (interquartile range) unless otherwise indicated.

Abbreviations: BSI = bloodstream infection, ICU = intensive care unit, UTI = urinary tract infection.

infection rate = number of infections / total patient–days × 1000

urinary catheter utilization ratio = number of urinary catheter-days / number of patient-days

ventilator utilization ratio = number of ventilator-days / number of patient-days

central line utilization ratio = number of central line-days / number of patient-days

**Q6. Not all ICUs apparently have quarters with infections with the 3 different bacterial species; this must also be an interesting number to follow? And why do they not have these infections - because they do not monitor or because they are better to treat their patients?**

**Response :**

Thank you for your valuable suggestion. There are indeed ICUs in which infections (pneumonia, UTI, BSI) caused by the three pathogens discussed in this paper were not observed, and studying the common characteristics of these ICUs could be very helpful for infection management. Since hospitals differ in size and capacity, and the severity of patients also varies, we will consider this aspect as a potential subject for future research.

**Reviewer #4’s comments:**

**Q1. Why was only one organism chosen per ICU, when multiple drug-resistant bacteria cause infection in ICUs?**

**Response :**

Thank you for your important question. In this paper, we address three types of bacteria: *K. pneumoniae*, *P. aeruginosa*, and *A. baumannii*. For each ICU, we analyzed the transition from carbapenem-susceptible to carbapenem-resistant strains in the same manner for each of the three types of bacteria. Although the same factors may influence the results, we believed that the bacteria might respond differently, so we conducted separate analyses for each individual bacteria.

**Q2. How did the authors find arrow categories as useful tools in infection control, as compared to the conventional method, as there is no comparative data?**

**Response :**

Thank you for your insightful comment. The method introduced in this study was designed as a novel approach to enhance the convenience of surveillance system operations. As such, the absence of comparable conventional methods can be considered both an advantage and a limitation. In practical applications, it will be necessary to gradually incorporate the proposed method, accumulate data, and refine the approach over time.

**Q3. How did they identify factors for reducing infections?**

**Response :**

Thank you for your important question. Firstly, it may be possible to identify significant factors by comparing the specific characteristics of ICUs in the green category with those in the red category. Secondly, another approach could involve identifying the common characteristics of ICUs that were initially in the red category but later transitioned to the green category.

**Q4. As the authors have mentioned, there are several limitations to the study; it would be prudent to carry out a pilot study to compare changes in drug-resistant bacteria in ICUs by the conventional method to substantiate their claim.**

**Response :**

Thank you for your valuable comments. In this study, we introduced the visualization of raw data through data processing. Although the supporting evidence remains limited, our primary focus was on the potential applicability of this new method. In the future, we plan to gradually implement this approach in practical settings, accumulate relevant data, and conduct pilot studies as you suggested.

**Reviewer #5’s comments:**

**Q1. In my opinion, the discussion could still be better. At least comparison of the current observatory methods proposed by the authors could be made with previous methods. No citation was made in the entire discussion.**

**Response :**

Thank you for your thoughtful feedback. In this study, we proposed a method for visualizing raw data through data processing. The method presented in this study was developed as a novel approach to improve the efficiency of surveillance system operations. Given this, the lack of comparable conventional methods can be seen as both a strength and a limitation. In real-world applications, a step-by-step implementation of the proposed method will be essential, along with continuous data accumulation and iterative refinement.

**Q2. Also, the authors could help readers by briefly summarizing at what points Student’s t-test, Mann Whitney test, chi-square test, and Fisher’s exact test were employed.**

**Response :**

Thank you for your insightful comment. In table 1 and 2, SPSS (Student’s t-test, Mann-Whitney test, chi-square test, Fisher’s exact test) was used. For comparing continuous data, Student’s t-test was applied when the normality test conditions were met, and Mann-Whitney test was used when the normality test conditions were not satisfied. For comparing categorical data, chi-square test was used, and Fisher’s exact test was applied when more than 20% of the cells had expected frequencies less than 5.

**In line 204 of the revised manuscript,**

**Original:**

Python and Microsoft Excel were used for the overall data extraction process, classification, and graph drawing (supplementary material #2). Tables and some graphs were created using Microsoft Excel, Microsoft Power point and SPSS (Student’s t-test, Mann-Whitney test, chi-square test, Fisher’s exact test).

**Revised:**

Python and Microsoft Excel were used for the overall data extraction process, classification, and graph drawing (supplementary material #3). In table 1 and 2, SPSS (Student’s t-test, Mann-Whitney test, chi-square test, Fisher’s exact test) was used. For comparing continuous data, Student’s t-test was applied when the normality test conditions were met, and Mann-Whitney test was used when the normality test conditions were not satisfied. For comparing categorical data, chi-square test was used, and Fisher’s exact test was applied when more than 20% of the cells had expected frequencies less than 5

**Reviewer #6’s comments:**

**Response :**

Thank you for your valuable comments. Through various revisions and improvements, we hope that this work can be of some assistance to infectious disease scholars and those involved in infection surveillance.

**Reviewer #7’s comments:**

**Q1. Validation of Graph Transformation Technique: How do the results from graph transformation compare to standard trend analysis methods, such as regression or time-series analysis? Do the ICU categories (red, yellow, green) yield the same conclusions in different validation datasets?**

**If feasible, compare the graph transformation method with traditional methods. Including even a brief mention of ongoing validation efforts in the discussion would increase confidence in the approach i.e. the validity of the study**

**Response :**

Thank you for your insightful feedback. The graph transformation method does not generate entirely new findings but rather restructures existing data to enhance its readability for KONIS operators. Because this approach differs fundamentally from traditional methods, a direct comparison is not straightforward. Thus, this paper focuses on introducing the potential of this new technique. In the future, we aim to gradually apply this method in practice, accumulate data, and conduct further validation and refinements to assess its effectiveness.

**Q2. Interpretation of Polarization in Pseudomonas aeruginosa Trends: While the authors note an unexpected rise in both "red" and "green" ICUs for P. aeruginosa, they do not explore potential reasons in depth. Providing specific ideas or analyzing the characteristics of the ICUs could enhance this discussion.**

**Expand the discussion on the polarization phenomenon observed with Pseudomonas aeruginosa.**

**Response :**

Thank you for your insightful comment. In the analysis of *P. aeruginosa*, the proportion of newly participating ICUs in the green and red categories in the last 5 years of KONIS, which initially did not participate in KONIS but joined in the later stages, was 78.5% and 76%, respectively. Most of the newly participating ICUs in the later stages of KONIS were small and medium-sized hospitals. It is speculated that the polarization observed may have been due to the lack of standardized infection control protocols, with significant differences between hospitals.

**In line 264 of the revised manuscript,**

**Original:**

In the case of PA, the proportion of aggressive ICUs, characterized by a rapid transition from CSPA to CRPA with a simultaneous increase in the infection rate, increased. Concurrently, the proportion of ICUs showing a reversal in the infection trend from CRPA to CSPA increased. This polarization phenomenon is an unexpected outcome. Therefore, a comparative analysis of the aggressive ICU characteristics and reverse ICU characteristics is needed to identify the key factors influencing this pattern.

**Revised:**

In the case of PA, the proportion of aggressive ICUs, characterized by a rapid transition from CSPA to CRPA with a simultaneous increase in the infection rate, increased. Concurrently, the proportion of ICUs showing a reversal in the infection trend from CRPA to CSPA increased. This polarization phenomenon is an unexpected outcome. Therefore, a comparative analysis of the aggressive ICU characteristics and reverse ICU characteristics is needed to identify the key factors influencing this pattern. In the analysis of *P. aeruginosa*, the proportion of newly participating ICUs in the green and red categories in the last 5 years of KONIS, which initially did not participate in KONIS but joined in the later stages, was 78.5% and 76%, respectively. Most of the newly participating ICUs in the later stages of KONIS were small and medium-sized hospitals. It is speculated that the polarization observed may have been due to the lack of standardized infection control protocols, with significant differences between hospitals.

**Q3. Generality and Broader Implications: The revised manuscript has improved its technical analysis but needs a more robust discussion about how the method can be applied across different healthcare systems. How well can this method adapt to systems with varying surveillance setups or different types of pathogens?**

**Add a section discussing how this method could be adapted to other situations or pathogens, this will strengthen the generalizability of the study.**

**Response :**

Thank you for your valuable comments. If sufficient data were available, it might be possible to approach the issue in the same manner by varying the combinations of pathogens and antibiotics/antivirals/antifungals. This could be done on a larger scale, such as at the national level, or on a smaller scale, such as at the hospital or even the ward level. However, selecting the appropriate reference values (e.g., cut-off length, scale-up factor) would likely be a crucial aspect of this approach.

**In line 299 of the revised manuscript,**

**Original:**

In this paper, existing data was transformed through a series of processes to visualize it in a single graph, making it easier to understand the situation across hundreds of ICUs. But to fully evaluate its usefulness, numerous follow-up studies and the judgment of readers will be necessary.

**Revised:**

~~In this paper, existing data was transformed through a series of processes to visualize it in a single graph, making it easier to understand the situation across hundreds of ICUs. But to fully evaluate its usefulness, numerous follow-up studies and the judgment of readers will be necessary.~~

This approach addresses key limitations of traditional methods, which rely on overall summary statistics or ICU specific trend graphs over many years often impractical for large-scale surveillance. The new method allows a comprehensive and scalable view of surveillance data, accommodating the growing number of participating ICUs and enabling clearer insights into individual ICU trends.

If sufficient data were available, it might be possible to approach the issue in the same manner by varying the combinations of pathogens and antibiotics/antivirals/antifungals. Also this could be done on a larger scale, such as at the national level, or on a smaller scale, such as at the hospital or even the ward level. However, selecting the appropriate reference values (e.g., cut-off length, scale-up factor) would likely be a crucial aspect of this approach.

**Q4. Addressing Reviewer Concerns About Bias: Reviewers expressed concerns about potential bias due to excluded ICUs and the small sample size in earlier years. For instance, only three ICUs were analyzed for Klebsiella pneumoniae in the first five years. The authors mention that the data is consistent but should discuss how these exclusions could impact the results. They should also clarify whether they conducted any sensitivity analyses.**

**Response :**

Thank you for your insightful comment. The selection criteria for ICUs reflect the minimal conditions required to satisfy the calculation formula of the vector transformation process. ICUs that do not meet these conditions cannot undergo vector transformation even if included, making analysis impossible. In the case of Klebsiella pneumoniae during the first five years, the number of included ICUs was significantly insufficient, requiring careful interpretation. Since the primary objective of this study is to introduce the vector transformation process, we plan to conduct further research using additional data accumulated through the operation of KONIS in the future.

**Q5. Ethical Considerations and Data Accessibility: Although the authors explained ethical approvals and how they maintained information privacy, replicating the study remains challenging due to limited data access. Including a synthetic dataset or providing clear instructions for repeating the analysis using publicly available data would enhance the manuscript's utility. If feasible, provide a synthetic dataset or an example analysis pipeline in the supplementary materials to enable readers to apply the method to their own data.**

**Response :**

Thank you for your valuable feedback. Due to security restrictions, we are unable to provide the raw data. Instead, we have generated and included a synthetic dataset. Additionally, we analyzed the synthetic dataset using the Python code provided in Supplementary Materials 3 and have included the corresponding results.

**In line 339 of the revised manuscript,**

**Original:**

**Data Availability:** Data cannot be shared publicly because of potentially sensitive information and the terms under which the data were collected indicated the data were to be used only for the purposes outlined in this study. Data may be available from KONIS ICU Steering Committee Office (contact via konis2@koshic.org) for researchers who meet the criteria for access to confidential data.

**Revised:**

**Data Availability:** Data cannot be shared publicly because of potentially sensitive information and the terms under which the data were collected indicated the data were to be used only for the purposes outlined in this study. Instead, we have generated and included a synthetic dataset. Additionally, we analyzed the synthetic dataset using the python code provided in Supplementary Materials 2 and have included the corresponding results.

**Reviewer #8’s comments:**

**Q1. (General comment) Please avoid the use of the same word more than once in a sentence. (e.g. "Graph transformation was employed to convert the infection rate graphs of each ICU into arrows." graph and rate graphs ...).**

**Response :**

Thank you for your insightful comments. We have revised the manuscript as follows.

**In line 39 of the revised manuscript,**

**Original:**

Graph transformation was employed to convert the infection rate graphs of each ICU into arrows.

**Revised:**

Transformation was employed to convert the infection rate graphs of each ICU into arrows.

**Q2. (General comment) Do not start a sentence with an abbreviation (e.g. UTI, BSI).**

**Response :**

Thank you for your detailed and insightful comments. We have revised the manuscript accordingly, as shown below.

**In line 114 of the revised manuscript,**

**Original:**

UTI was diagnosed if one of the following criteria was present:

**Revised:**

Urinary tract infection was diagnosed if one of the following criteria was present:

**In line 118 of the revised manuscript,**

**Original:**

BSI was defined as the presence of one or more pathogenic bacteria on blood culture or non–culture–based testing

**Revised:**

Blood stream infection was defined as the presence of one or more pathogenic bacteria on blood culture or non–culture–based testing

**Q3. (General comment) Do not use abbreviations in section and subsections name (e.g., ICUs).**

**Response :**

Thank you for your valuable feedback. We have revised the manuscript as follows.

**In line 126 of the revised manuscript,**

**Original:**

ICUs

**Revised:**

Intensive care units

**In line 179 of the revised manuscript,**

**Original:**

ICU categorization based on arrow indicators

**Revised:**

Intensive care unit categorization based on arrow indicators

**In line 227 of the revised manuscript,**

**Original:**

The visualization of infection trends in ICUs

**Revised:**

The visualization of infection trends in Intensive care units

**Q4. (General comment) To be clinical relevant you should also include information about the total number of patients hospitalized in ICU in the investigated period.**

**Response :**

Thank you for your valuable feedback. As the length of hospital stays varies by patient, we have mentioned the patient-days in ICU in Table 1. In the analysis of *A. baumannii*, the average patient-days in ICU during the KONIS first 5 years (2006-2011) was 1710.00 (1361.35–2164.33), while the average patient-days in ICU during the KONIS last 5 years (2017-2022) was 1366.28 (1078.8–1743.75). In the analysis of *K. pneumoniae*, the average patient-days in ICU during the KONIS first 5 years (2006-2011) was 1756.75 (1696.88–2077.06), while the average patient-days in ICU during the KONIS last 5 years (2017-2022) was 1330.50 (1091.83–1731.90). In the analysis of *P. aeruginosa*, the average patient-days in ICU during the KONIS first 5 years (2006-2011) was 1804.36 (1332.98–2214.20), while the average patient-days in ICU during the KONIS last 5 years (2017-2022) was 1366.28 (1090.54–1847.91).

**Q5. (General comment) It has sense to report median value for qualitative variable? For example, how do you interpret Pneumonia rate of 1.39? From my point of view is useless. The same comment is also valid for Q1 and Q3.**

**Response :**

Thank you for your valuable feedback. In this paper, rather than focusing on the intrinsic meaning of the values themselves, we aimed to determine the central distribution points of both susceptible and resistant strains, and to analyze and interpret their relative positions (i.e., before/after, above/below).

**Q6. (General comment) One information should stay in only one place in the manuscript otherwise is duplicated (within manuscript duplication/triplication etc.). See for example "Significance and implications" section.**

**Response :**

Thank you for your valuable feedback. We have revised the manuscript as follows.

**In line 292 of the revised manuscript,**

**Original:**

**Significance and implications**

Through the series of graph transformation processes presented in this study, the trends in infections and resistant bacteria within individual ICUs within several years were condensed into a single arrow or dot. Subsequently, hundreds of ICUs subjected to monitoring are expressed as individual dots and plotted simultaneously on a single graph. The length and angle of the arrow reflect the transition tendency from antibiotic–susceptible to antibiotic–resistant bacteria and changes in the infection rate. Based on this information, we divided the ICUs subject to surveillance into red, yellow, and green categories. In addition, we divided the data into the first 5 years and the last 5 years of the KONIS, enabling the analysis of the proportional change in the ICUs in each category.

Through this process, we were able to easily observe the trends of infections and resistant bacteria in hundreds of ICUs under surveillance. This stands out as a strength of this study. Over time, the number of ICUs participating in KONIS has steadily increased, with more than 300 ICUs participating by 2022. Data from these ICUs could be collected and analyzed using traditional methods to determine the overall infection incidence rate and the proportion of resistant bacteria, as well as to observe changes over time. However, several inconveniences are associated with traditional methods. First, since the overall infection incidence rate and the proportion of resistant bacteria are representative values from all ICUs participating in KONIS rather than from individual ICUs, it is difficult to determine these values for each ICU and assess the current state of infection control. Additionally, analyzing with traditional methods requires plotting graphs for each ICU, reflecting data from as many as 17 years, which is practically very challenging. Second, it is difficult to simultaneously track and understand the situation in more than 300 ICUs using traditional methods. It is hard to grasp more than 300 graphs at a glance, and even more challenging to continually track them. The new method presented in this paper was developed out of practical necessity, as it has become increasingly difficult to understand and track the situation of surveillance targets with the growing number of ICUs participating in KONIS. In this paper, existing data was transformed through a series of processes to visualize it in a single graph, making it easier to understand the situation across hundreds of ICUs. But to fully evaluate its usefulness, numerous follow-up studies and the judgment of readers will be necessary.

**Revised:**

**Significance and implications**

Through the graph transformation processes presented in this study, trends in infections and resistant bacteria within individual ICUs over several years were condensed into a single arrow or dot. By plotting hundreds of ICUs as individual points on a single graph, we were able to intuitively visualize the transition tendencies from antibiotic-susceptible to antibiotic-resistant bacteria and changes in infection rates. Based on this visualization, ICUs were categorized into red, yellow, and green groups. We also compared the first and last five years of KONIS data to observe changes in group proportions over time.

This approach addresses key limitations of traditional methods, which rely on overall summary statistics or ICU specific trend graphs over many years often impractical for large-scale surveillance. The new method allows a comprehensive and scalable view of surveillance data, accommodating the growing number of participating ICUs and enabling clearer insights into individual ICU trends.

If sufficient data were available, it might be possible to approach the issue in the same manner by varying the combinations of pathogens and antibiotics/antivirals/antifungals. Also this could be done on a larger scale, such as at the national level, or on a smaller scale, such as at the hospital or even the ward level. However, selecting the appropriate reference values (e.g., cut-off length, scale-up factor) would likely be a crucial aspect of this approach.

**Q7. (Introduction) Avoid expressions such as "In recent years" in scientific writing.**

**Response :**

Thank you for your detailed comments. We have revised the manuscript accordingly, as shown below.

**In line 79 of the revised manuscript,**

**Original:**

In recent years, the burden of MDR Gram–negative bacterial infections in South Korea have increased significantly, highlighting the growing importance of the KONIS system

**Revised:**

The burden of MDR Gram–negative bacterial infections in South Korea have increased significantly, highlighting the growing importance of the KONIS system [11].

**Q8. (Introduction) Be specific when you say "increased" and let the reader from which number and when to which number - when.**

**Response :**

Thank you for your valuable feedback. We have revised the manuscript as follows.

**In line 59 of the revised manuscript,**

**Original:**

Worldwide, nosocomial and multi–drug resistant bacterial infections are increasing and emerge as an ongoing social issue [1-3]

**Revised:**

Worldwide, nosocomial and multi-drug resistant bacterial infections have increased significantly. For example, CRAB accounted for over 50% of carbapenem-resistant gram-negative bacilli in ICUs [1], CRE prevalence in the U.S. rose from 1.2% in 2001 to 4.2% in 2011 [2], and reports of carbapenemase-producing P. aeruginosa have increased globally since 2010 [3].

**Q9. (Methods) "The diseases analyzed were pneumonia, urinary tract infection (UTI), and bloodstream infection (BSI)." why these diseases?**

**Response :**

Thank you for your important feedback. This paper was written using KONIS data, and the diseases mentioned were selected because they are both common and significant in ICUs, as well as being under nationwide surveillance in ICUs through KONIS.

**Q10. (Methods) How many ICUs participated in KONIS?**

**Response :**

Thank you for your valuable feedback. In the first 5 years of KONIS (2006-2011), a total of 137 ICUs participated in KONIS, while in the last 5 years of KONIS (2017-2022), a total of 368 ICUs participated. This information is mentioned in Figure 3.

**Q11. (Methods) Briefly present the KONIS.**

**Response :**

Thank you for your valuable feedback. After careful consideration, we have provided a detailed explanation in the Introduction regarding what KONIS is, its role, and the type of data it collects and monitors.

**Q12. (Methods) "KONIS has collected and analyzed data on a quarterly basis according to its own guidelines" reference(s) is/are needed.**

**Response :**

Thank you for your valuable comments. We have revised the manuscript accordingly.

**In line 129 of the revised manuscript,**

**Original:**

KONIS has collected and analyzed data on a quarterly basis according to its own guidelines.

**Revised:**

KONIS has collected and analyzed data on a quarterly basis according to its own guidelines [4,5].

**Q13. (Statistical analysis) Please tell the readers what method was used when.**

**Response :**

Thank you for your comment. SPSS (Student’s t-test, Mann-Whitney test, chi-square test, Fisher’s exact test) was used in tables 1 and 2. For the comparison of continuous data, Student’s t-test was utilized when the normality assumption was satisfied, while the Mann-Whitney test was applied when the normality assumption was not met. For categorical data, the chi-square test was used, and Fisher’s exact test was employed when more than 20% of the cells had expected frequencies less than 5.

**In line 204 of the revised manuscript,**

**Original:**

Python and Microsoft Excel were used for the overall data extraction process, classification, and graph drawing (supplementary material #2). Tables and some graphs were created using Microsoft Excel, Microsoft Power point and SPSS (Student’s t-test, Mann-Whitney test, chi-square test, Fisher’s exact test).

**Revised:**

Python and Microsoft Excel were used for the overall data extraction process, classification, and graph drawing (supplementary material #3). In table 1 and 2, SPSS (Student’s t-test, Mann-Whitney test, chi-square test, Fisher’s exact test) was used. For comparing continuous data, Student’s t-test was applied when the normality test conditions were met, and Mann-Whitney test was used when the normality test conditions were not satisfied. For comparing categorical data, chi-square test was used, and Fisher’s exact test was applied when more than 20% of the cells had expected frequencies less than 5

**Q14. (Results) "Among the selected ICUs, the causative pathogen was classified into CS and CR bacteria according to the carbapenem susceptibility status. ICUs where CS and CR bacterial infections occurred for more than two quarters were finally selected." & "For each selected ICU, an overall infection rate versus time graph was generated using Python. Subsequently, graph transformation was performed to create arrows for each ICU, with the endpoints indicated in polar coordinates." & "Each point on the graph signifies the endpoint of the arrow of an individual ICU, and the arrow for each ICU is formed by connecting the indicated point and origin. The arrows representing individual ICUs had various lengths and angles, with the diversity in .. "This information belongs to the methods section.**

**Response :**

Thank you for your detailed comments. We have revised the manuscript accordingly.

**In line 214 of the revised manuscript,**

**Original:**

A total of 137 ICUs participated in the first 5 years (2006 to 2011) and 368 ICUs participated in the last 5 years (2017 to 2022). Among these, ICUs in which infections (pneumonia, UTI, and BSI) caused by KP, PA, and AB occurred in at least one quarter were selected. During the first 5 years, KP infection was reported in 122 ICUs, PA infection in 124 ICUs, and AB infection in 123 ICUs. In the last 5 years, KP infection was reported in 316 ICUs, PA infection in 316 ICUs, and AB infection was reported in 309 ICUs.

Among the selected ICUs, the causative pathogen was classified into CS and CR bacteria according to the carbapenem susceptibility status. ICUs where CS and CR bacterial infections occurred for more than two quarters were finally selected. A total of 3 ICUs with KP infection, 43 ICUs with AB infection, and 50 ICUs with PA infection in the first 5 years, and 81 ICUs with KP infection, 92 ICUs with PA infection, and 102 ICUs with AB infection in the last 5 years were included in the final analysis (Fig 3). The characteristics of ICUs included in this study were shown in Tables 1 and 2.

**Revised:**

A total of 137 ICUs participated in the first 5 years (2006 to 2011) and 368 ICUs participated in the last 5 years (2017 to 2022). Among these, ICUs in which infections (pneumonia, UTI, and BSI) caused by KP, PA, and AB occurred in at least one quarter were selected. During the first 5 years, KP infection was reported in 122 ICUs, PA infection in 124 ICUs, and AB infection in 123 ICUs. In the last 5 years, KP infection was reported in 316 ICUs, PA infection in 316 ICUs, and AB infection was reported in 309 ICUs.

~~among the selected ICUs, the causative pathogen was classified into CS and CR bacteria according to the carbapenem susceptibility status.~~ ICUs where CS and CR bacterial infections occurred for more than two quarters were finally selected. A total of 3 ICUs with KP infection, 43 ICUs with AB infection, and 50 ICUs with PA infection in the first 5 years, and 81 ICUs with KP infection, 92 ICUs with PA infection, and 102 ICUs with AB infection in the last 5 years were included in the final analysis (Fig 3). The characteristics of ICUs included in this study were shown in Tables 1 and 2.

**In line 228 of the revised manuscript,**

**Original:**

For each selected ICU, an overall infection rate versus time graph was generated using Python. Subsequently, graph transformation was performed to create arrows for each ICU, with the endpoints indicated in polar coordinates. The graph below shows a collection of the arrow endpoints corresponding to the ICUs associated with each pathogen and observation period (Fig 4). Each point on the graph signifies the endpoint of the arrow of an individual ICU, and the arrow for each ICU is formed by connecting the indicated point and origin. The arrows representing individual ICUs had various lengths and angles, with the diversity in both dimensions increasing more in the last 5 years compared with that in the first 5 years for all pathogens.

**Revised:**

~~For each selected ICU, an overall infection rate versus time graph was generated using Python. Subsequently, graph transformation was performed to create arrows for each ICU, with the endpoints indicated in polar coordinates~~. The graph below shows a collection of the arrow endpoints corresponding to the ICUs associated with each pathogen and observation period (Fig 4). ~~Each point on the graph signifies the endpoint of the arrow of an individual ICU, and the arrow for each ICU is formed by connecting the indicated point and origin.~~ ~~The arrows representing individual ICUs had various lengths and angles, with the diversity in both dimensions increasing more in the last 5 years compared with that in the first 5 years for all pathogens.~~

Each ICU is represented as a single point, with all monitored ICUs simultaneously displayed on one graph. Vectors are drawn from the origin to each point, and each vector reflects the changes in the distribution of susceptible and resistant bacterial groups, as well as the infection rate, at each individual ICU.

**Q15. (Results) "However, since only three ICUs were analyzed in the first 5 years, caution should be observed when interpreting the results." This is not a result.**

**Response :**

Thank you for your valuable comments. We have revised the manuscript accordingly.

**In line 237 of the revised manuscript,**

**Original:**

The proportions of ICUs in the yellow and green categories decreased (Fig 5 C and D). However, since only three ICUs were analyzed in the first 5 years, caution should be observed when interpreting the results.

**Revised:**

For KP infection, the proportion of ICUs in the red category increased in the last 5 years compared to the first 5 years, while the proportions in the yellow and green categories decreased (Fig. 5C and D).

**Q16. (Results) Pay attention to duplication of results in text and table/figure. A result must stay either in text or in table/figure.**

**Response :**

Thank you for your valuable comments. We have revised the manuscript accordingly.

**In line 237 of the revised manuscript,**

**Original:**

For KP infection in the first 5 years (3 ICUs), 0% belonged in the red category (0 ICU), 33.3% in the yellow category (1 ICU), 66.6% in the green category (2 ICUs), and 0% in the undetermined category (0 ICU). In the last 5 years (81 ICUs), 17% belonged in the red category (14 ICUs), 7% in the yellow category (6 ICUs), 36% in the green category (29 ICUs), and 40% in the undetermined category (32 ICUs). The proportion of red category ICUs increased in the last 5 years compared with that in the first 5 years. The proportions of ICUs in the yellow and green categories decreased (Fig 5 C and D).

For PA infection, in the first 5 years (total 50 ICUs), there were 12% in the red category (6 ICUs), 8% in the yellow category (4 ICUs), 38% in the green category (19 ICUs), and 42% were undetermined (21 ICUs). In the last 5 years (92 ICUs), the red category was 27% (25 ICUs), the yellow category was 2% (2 ICUs), the green category was 46% (42 ICUs), and the undetermined category was 25% (23 ICUs). The proportion of red and green category ICUs increased and yellow category ICUs decreased in the last 5 years compared to the first 5 years. (Fig 5 E and F).

For AB infections in the first 5 years (43 ICUs), 19% were classified in the red category (8 ICUs), 5% in the yellow category (2 ICUs), 19% in the green category (8 ICUs), and 58% in the undetermined category (25 ICUs). In the last 5 years (102 ICUs), 14% were classified in the red category (14 ICUs), 1% in the yellow category (1 ICU), 72% in the green category (74 ICUs), and 13% in the undetermined category (13 ICUs). The proportion of red and yellow category ICUs decreased in the last 5 years compared with that in the first 5 years. Meanwhile, the proportion of ICUs in the green category increased (Fig 5 A and B).

**Revised:**

For KP infection, the proportion of ICUs in the red category increased in the last 5 years compared to the first 5 years, while the proportions in the yellow and green categories decreased (Fig. 5C and D). For PA infection, both the red and green category ICUs increased in proportion, while the yellow category decreased during the last 5 years compared to the first 5 years (Fig. 5E and F). For AB infection, the proportion of ICUs in the green category increased, whereas the red and yellow categories decreased in the last 5 years compared with the first 5 years (Fig. 5A and B).

**Q17. (Results) If the value of Q1 and Q3 are included in the ranges you should use squared brackets ("[]").**

**Response :**

Thank you for your valuable comments. We have revised the manuscript accordingly.

**In line 392 of the revised manuscript,**

**Original:**

**Revised:**

**In line 398 of the revised manuscript,**

**Original:**

**Revised:**

**Q18. (Discussion) The shifts in trends must be explains (ex. more medical and less surgical in the second period compared to the first period ... why?).**

**Response :**

Thank you for your insightful comment. We have revised the manuscript accordingly.

**In line 276 of the revised manuscript,**

**Original:**

In the case of AB, the proportion of aggressive ICUs with a rapid transition from CSAB to CRAB and a simultaneous increase in infection rate decreased. Similarly, the proportion of indolent ICUs with a slow transition and simultaneously decreasing infection rate decreased. Conversely, the proportion of ICUs where the infection trend reversed from CRAB to CSAB increased. From an infection control perspective, these findings suggest a positive trend, and further studies are needed to determine the ICU characteristics contributing to these outcomes.

**Revised:**

In the case of AB, the proportion of aggressive ICUs with a rapid transition from CSAB to CRAB and a simultaneous increase in infection rate decreased. Similarly, the proportion of indolent ICUs with a slow transition and simultaneously decreasing infection rate decreased. Conversely, the proportion of ICUs where the infection trend reversed from CRAB to CSAB increased. From an infection control perspective, these findings suggest a positive trend, and further studies are needed to determine the ICU characteristics contributing to these outcomes.

In the latter 5 years of KONIS, the proportion of medical ICUs increased compared to surgical ICUs. This may be due to the expansion of participation by small- and medium-sized hospitals, which typically have more medical than surgical ICUs. Additionally, increased national attention to infection control and antimicrobial stewardship, especially in high-risk medical ICUs with prolonged stays and frequent antibiotic use, may have encouraged greater participation from these units. These factors likely contributed to the observed trend shifts between the two periods.

**Q19. (Discussion) The method you propose is at the seems to be useful at the level of hospital and you should discuss how these results could be used.**

**Response :**

Thank you for your valuable feedback. On a larger scale, comparisons could be made at the national level, on a smaller scale at the hospital level, and even at the ward level. However, the selection of appropriate reference values (such as cut-off length and scale-up factor) would be a key consideration in this approach.

**In line 299 of the revised manuscript,**

**Original:**

In this paper, existing data was transformed through a series of processes to visualize it in a single graph, making it easier to understand the situation across hundreds of ICUs. But to fully evaluate its usefulness, numerous follow-up studies and the judgment of readers will be necessary.

**Revised:**

This approach addresses key limitations of traditional methods, which rely on overall summary statistics or ICU specific trend graphs over many years often impractical for large-scale surveillance. The new method allows a comprehensive and scalable view of surveillance data, accommodating the growing number of participating ICUs and enabling clearer insights into individual ICU trends.

If sufficient data were available, it might be possible to approach the issue in the same manner by varying the combinations of pathogens and antibiotics/antivirals/antifungals. Also this could be done on a larger scale, such as at the national level, or on a smaller scale, such as at the hospital or even the ward level. However, selecting the appropriate reference values (e.g., cut-off length, scale-up factor) would likely be a crucial aspect of this approach.

**Reviewer #9’s comments:**

**Q1. The explanation of the graph transformation method is concise, but it might be too technical for readers unfamiliar with the concept. A brief explanation of why this method was chosen could enhance understanding, and it is better to emphasize the potential impact of the findings on healthcare practices and policies.**

**Response :**

Thank you for your important question. As KONIS monitors hundreds of ICUs, it became challenging to interpret the information at a glance. In an effort to find a more convenient and intuitive method, we arrived at the approach presented in this paper. If this method allows for effective monitoring of hundreds of ICUs simultaneously and in a way that is easy to understand, it could, in turn, contribute to improving infection control efforts in the future, even if only in a small way. For a better understanding of why this particular method was chosen, we kindly refer the reader to the discussion provided in the main text below. We have also included additional information on its application, and a more detailed equation has been provided in Supplementary Material #2 to facilitate comprehension

**In line 140 of the revised manuscript,**

**Original:**

From a macroscopic perspective, it was hypothesized that a physical force exists which transforms the susceptible bacterial population into a resistant bacterial population. This force was believed to reflect various factors, including antimicrobial stewardship and infection control. Since vectors are commonly used to represent force in physics, this concept was applied in the study.

**In line 161 of the revised manuscript,**

**Revised:**

The several equations were used for vector calculation (supplementary material #2).

**In line 299 of the revised manuscript,**

**Original:**

In this paper, existing data was transformed through a series of processes to visualize it in a single graph, making it easier to understand the situation across hundreds of ICUs. But to fully evaluate its usefulness, numerous follow-up studies and the judgment of readers will be necessary.

**Revised:**

This approach addresses key limitations of traditional methods, which rely on overall summary statistics or ICU specific trend graphs over many years often impractical for large-scale surveillance. The new method allows a comprehensive and scalable view of surveillance data, accommodating the growing number of participating ICUs and enabling clearer insights into individual ICU trends.

If sufficient data were available, it might be possible to approach the issue in the same manner by varying the combinations of pathogens and antibiotics/antivirals/antifungals. Also this could be done on a larger scale, such as at the national level, or on a smaller scale, such as at the hospital or even the ward level. However, selecting the appropriate reference values (e.g., cut-off length, scale-up factor) would likely be a crucial aspect of this approach.

**Q2. The introduction should clearly articulate the research problem and the significance of the study. This is essential to ensure that the reader understands the research's context and necessity. A lack of clarity in the introduction may confuse readers about the study's purpose and scope. The introduction should include a brief review of existing literature related to the study. This helps identify gaps in current knowledge and justify the need for the research. It is important to ensure that the literature review is up-to-date and relevant.**

**Response :**

Thank you for your important question. We have revised the manuscript as follows.

**In line 72 of the revised manuscript,**

**Original:**

Even during the coronavirus disease 2019 (COVID–19) pandemic, active surveillance was conducted using the KONIS. Research was performed to determine the impact of COVID–19 on healthcare–associated infections in the ICU [10].

The KONIS is used to monitor trends in specific site and device–related infections and resistant bacteria in the ICU. The burden of MDR Gram–negative bacterial infections in South Korea have increased significantly, highlighting the growing importance of the KONIS system [11]. However, more than 300 ICUs are subject to surveillance, and the surveillance duration varies for each ICU, extending up to 16 years. Despite of this variability, the graphical representation of infection and antibiotic–resistant bacteria trends within each ICU is possible. However, comprehending this information can pose a significant challenge. Simultaneously acquiring surveillance results from hundreds of ICUs makes it even more challenging to evaluate and improve infection control. Hence, this study aimed to visualize the trends in infections and MDR gram–negative bacterial infections in hundreds of ICUs monitored by the KONIS.

**Revised:**

Even during the coronavirus disease 2019 (COVID–19) pandemic, active surveillance was conducted using the KONIS. Research was performed to determine the impact of COVID–19 on healthcare–associated infections in the ICU [10]. However, most previous studies have focused on aggregated data or infection types, with limited investigation into infection and resistance trends at the individual ICU level.

The KONIS is used to monitor trends in specific site and device–related infections and resistant bacteria in the ICU. The burden of MDR Gram–negative bacterial infections in South Korea have increased significantly, highlighting the growing importance of the KONIS system [11]. Given that more than 300 ICUs are monitored in KONIS, with varying surveillance durations extending up to 16 years, it remains difficult to effectively assess and compare ICU-specific infection trends. This complexity hinders real-time interpretation and the development of tailored infection control strategies at the ICU level.

Hence, this study aimed to visualize trends in infections and MDR gram-negative bacterial infections across individual ICUs using KONIS data. By applying a novel graph transformation method, we sought to condense complex surveillance results into interpretable patterns, enabling more intuitive comparisons and insights into ICU-specific resistance trends over time.

**Q3. The method section should provide sufficient detail to allow other researchers to replicate the study. This includes a comprehensive description of the data sources, the process of graph transformation, and how the infection rates were converted into arrows. The study uses data from the Korean National Healthcare-associated Infections Surveillance System (KONIS), covering 137 ICUs from 2006 to 2011 and 368 ICUs from 2017 to 2022. It is important to ensure that the selection criteria for these data sources are clearly explained, including any inclusion or exclusion criteria.**

**Response :**

Thank you for pointing out this important aspect. To facilitate the replication of our study by other researchers, we have included a synthetic dataset and Python code. Additionally, we have organized and provided the relevant equations to aid understanding.

**In line 161 of the revised manuscript,**

**Revised:**

The several equations were used for vector calculation (supplementary material #2).

**In line 203 of the revised manuscript,**

**Original:**

Python and Microsoft Excel were used for the overall data extraction process, classification, and graph drawing (supplementary material #2). Tables and some graphs were created using Microsoft Excel, Microsoft Power point and SPSS (Student’s t-test, Mann-Whitney test, chi-square test, Fisher’s exact test).

**Revised:**

Python and Microsoft Excel were used for the overall data extraction process, classification, and graph drawing (supplementary material #3). In table 1 and 2, SPSS (Student’s t-test, Mann-Whitney test, chi-square test, Fisher’s exact test) was used. For comparing continuous data, Student’s t-test was applied when the normality test conditions were met, and Mann-Whitney test was used when the normality test conditions were not satisfied. For comparing categorical data, chi-square test was used, and Fisher’s exact test was applied when more than 20% of the cells had expected frequencies less than 5

**In line 339 of the revised manuscript,**

**Original:**

**Data Availability:** Data cannot be shared publicly because of potentially sensitive information and the terms under which the data were collected indicated the data were to be used only for the purposes outlined in this study. Data may be available from KONIS ICU Steering Committee Office (contact via konis2@koshic.org) for researchers who meet the criteria for access to confidential data.

**Revised:**

**Data Availability:** Data cannot be shared publicly because of potentially sensitive information and the terms under which the data were collected indicated the data were to be used only for the purposes outlined in this study. Instead, we have generated and included a synthetic dataset. Additionally, we analyzed the synthetic dataset using the python code provided in Supplementary Materials 2 and have included the corresponding results.

**Q4. While the discussion acknowledges the limitations, it could benefit from a deeper analysis of their potential implications. For instance, discussing how these limitations might affect the results' interpretation or the findings' generalizability would provide a more comprehensive understanding. The discussion could be strengthened by comparing the new method with existing methods for monitoring infection trends.**

**Response :**

Thank you for your valuable comments. In this study, we introduced the visualization of raw data through data processing. Although the supporting evidence remains limited, our primary focus was on the potential applicability of this new method. In the future, we plan to gradually implement this approach in practical settings, accumulate relevant data, and conduct pilot.

**In line 299 of the revised manuscript,**

**Original:**

In this paper, existing data was transformed through a series of processes to visualize it in a single graph, making it easier to understand the situation across hundreds of ICUs. But to fully evaluate its usefulness, numerous follow-up studies and the judgment of readers will be necessary.

**Revised:**

This approach addresses key limitations of traditional methods, which rely on overall summary statistics or ICU specific trend graphs over many years often impractical for large-scale surveillance. The new method allows a comprehensive and scalable view of surveillance data, accommodating the growing number of participating ICUs and enabling clearer insights into individual ICU trends.

If sufficient data were available, it might be possible to approach the issue in the same manner by varying the combinations of pathogens and antibiotics/antivirals/antifungals. Also this could be done on a larger scale, such as at the national level, or on a smaller scale, such as at the hospital or even the ward level. However, selecting the appropriate reference values (e.g., cut-off length, scale-up factor) would likely be a crucial aspect of this approach.

We have revised our paper as described above and we believe we have addressed all questions and comment, but would be happy to provide further information or revision if necessary. Thank you for your consideration.

Sincerely yours,

Mi Suk Lee, MD, PhD

Professor,

Division of Infectious Diseases,

Department of Internal Medicine,

Kyung Hee University College of Medicine,

Kyung Hee University Hospital,

23, Kyung Hee Dae-ro, Dongdaemungu, Seoul, 02447,

Republic of Korea,

Phone: (82) 2 9588159, Fax number: (82) 2 9681848,

E-mail: mslee7@gmail.com
