# Supplementary material for: Visualization of multidrug-resistant bacterial infection trends in the intensive care units
Source: PLoS One. 2025 Aug 28;20(8):e0330765. doi: 10.1371/journal.pone.0330765 (PMC12393710; doi:10.1371/journal.pone.0330765)
Supplement: Attachment — Submitted filename: Response_to_Reviewers_auresp_3.docx [file pone.0330765.s007.docx]

3 July 2025

Ali Amanati

Academic Editor

PLOS ONE

Re: PONE-D-24-03240R2,

“Visualization of multidrug-resistant bacterial infection trends in the intensive care units”

**Dr. Ali Amanati:**

On behalf of the authors, I would like to thank you and the reviewers for helpful comments on our submitted manuscript, “Visualization of multidrug-resistant bacterial infection trends in the intensive care units”. We have carefully reviewed the comments and have revised the manuscript accordingly.

**Reviewer #5’s comments:**

**Q1. In my opinion, the discussion could still be better. At least comparison of the current observatory methods proposed by the authors could be made with previous methods. No citation was made in the entire discussion.**

**Response :**

Thank you for your thoughtful feedback. We have revised it as follows.

**In line 309 of the revised manuscript,**

**Original:**

This approach addresses key limitations of traditional methods, which rely on overall summary statistics or ICU specific trend graphs over many years often impractical for large-scale surveillance. The new method allows a comprehensive and scalable view of surveillance data, accommodating the growing number of participating ICUs and enabling clearer insights into individual ICU trends.

**Revised:**

This approach addresses key limitations of traditional methods, which rely on overall summary statistics or ICU specific trend graphs over many years, often impractical for large-scale surveillance. While systems such as GLASS (Global Antimicrobial Resistance Surveillance System) and NHSN (National Healthcare Safety Network) provide valuable aggregated data, they do not offer a granular view of resistance dynamics at the individual ICU level [15, 16]. The new method allows a more intuitive, comprehensive, and scalable view of surveillance data, accommodating the growing number of participating ICUs and enabling clearer insights into unit-specific trends.

**In line 404 of the revised manuscript,**

**Revised:**

15	World Health Organization. Global antimicrobial resistance and use surveillance system (GLASS) report 2022*.* Geneva: WHO; 2022.

16	Centers for Disease Control and Prevention*.* National Healthcare Safety Network (NHSN): Antimicrobial Use and Resistance (AUR) Module*.* Atlanta: CDC; 2023.

**Q2. Also, the authors could help readers by briefly summarizing at what points Student’s t-test, Mann Whitney test, chi-square test, and Fisher’s exact test were employed.**

**Response :**

Thank you for your insightful comment. In table 1 and 2, SPSS (Student’s t-test, Mann-Whitney test, chi-square test, Fisher’s exact test) was used. For comparing continuous data, Student’s t-test was applied when the normality test conditions were met, and Mann-Whitney test was used when the normality test conditions were not satisfied. For comparing categorical data, chi-square test was used, and Fisher’s exact test was applied when more than 20% of the cells had expected frequencies less than 5.

**In line 213 of the revised manuscript,**

**Original:**

Python and Microsoft Excel were used for the overall data extraction process, classification, and graph drawing (supplementary material #2). Tables and some graphs were created using Microsoft Excel, Microsoft Power point and SPSS (Student’s t-test, Mann-Whitney test, chi-square test, Fisher’s exact test).

**Revised:**

Python and Microsoft Excel were used for the overall data extraction process, classification, and graph drawing (supplementary material #3). In table 1 and 2, SPSS (Student’s t-test, Mann-Whitney test, chi-square test, Fisher’s exact test) was used. For comparing continuous data, Student’s t-test was applied when the normality test conditions were met, and Mann-Whitney test was used when the normality test conditions were not satisfied. For comparing categorical data, chi-square test was used, and Fisher’s exact test was applied when more than 20% of the cells had expected frequencies less than 5

**Reviewer #8’s comments:**

**Q1. (Abstract) It is not sufficiently clear why you choose the two periods or bacteria.**

**Response :**

Thank you for your important question. The analysis of KONIS data for this study began in 2023. Although data from the period 2006 to 2022 were available, the volume was too large to analyze the entire dataset comprehensively. Therefore, we limited our analysis to the initial five years and the most recent five years of the KONIS data to ensure feasibility and focus. As for the bacterial species, we selected *Klebsiella pneumoniae*, *Pseudomonas aeruginosa*, and *Acinetobacter baumannii*, as these organisms are of particular concern due to their association with emerging antibiotic resistance in recent years. Gram-positive bacteria such as MRSA were planned to be addressed in a subsequent study.

**Q2. (Abstract) Is increase/decrease statistically significant?**

**Response :**

Thank you for your insightful question. We have revised it as follows.

**In line 47 of the revised manuscript,**

**Original:**

**Results:** For *K. pneumoniae*, the proportion of red category ICUs increased (0% to 17%), while the proportions of yellow (33.3% to 7%) and green category ICUs (66.6% to 36%) decreased. For *P. aeruginosa*, the proportions of red (12% to 27%) and green category ICUs (38% to 46%) increased, while the proportion of yellow category ICUs decreased (8% to 2%). For *A. baumannii*, the proportions of red (19% to 14%) and yellow category ICUs (5% to 1%) decreased, while the proportion of green category ICUs increased (19% to 72%).

**Revised:**

**Results:** For *K. pneumoniae*, the proportion of red category ICUs increased (0% to 17%, p-value 0.586), while the proportions of yellow (33.3% to 7%, p-value 0.288) and green category ICUs (66.6% to 36%, p-value 0.290) decreased. For *P. aeruginosa*, the proportions of red (12% to 27%, p-value 0.016) and green category ICUs (38% to 46%, p-value 0.358) increased, while the proportion of yellow category ICUs decreased (8% to 2%, p-value 0.043). For *A. baumannii*, the proportions of red (19% to 14%, p-value 0.649) and yellow category ICUs (5% to 1%, p-value 0.187) decreased, while the proportion of green category ICUs increased (19% to 72%, p-value <0.001).

**Q3. (Introduction) Define abbreviations first time when are used in the body of the manuscript (CRAB. ICU, CRE).**

**Response :**

Thank you for your insightful question. We have revised it as follows.

**In line 63 of the revised manuscript,**

**Original:**

For example, CRAB accounted for over 50% of carbapenem-resistant gram-negative bacilli in ICUs [1], CRE prevalence in the U.S. rose from 1.2% in 2001 to 4.2% in 2011 [2], and reports of carbapenemase-producing P. aeruginosa have increased globally since 2010 [3].

**Revised:**

For example, carbapenem-resistant *Acinetobacter baumannii* (CRAB) accounted for over 50% of carbapenem-resistant gram-negative bacilli in intensive care units (ICUs) [1], carbapenem-resistant *Enterobacterales* (CRE) prevalence in the U.S. rose from 1.2% in 2001 to 4.2% in 2011 [2], and reports of carbapenemase-producing P. aeruginosa have increased globally since 2010 [3].

**Q4. (Introduction) It is unclear why the reported prevalence is for US but the study is done on Korean data.**

**Response :**

Thank you for your important question. The Korean National Healthcare–associated Infections Surveillance System (KONIS) was established by benchmarking the United States National Nosocomial Infections Surveillance System (NNIS). Given the global significance of carbapenem-resistant gram-negative bacterial infections, we believe this trend is not unique to Korea. Therefore, we have referenced studies from other countries as well. Since KONIS and NNIS are similar surveillance systems, we believe that the visualization method presented in this study could also be applicable to NNIS.

**Q5. (Introduction) How data are reported to KONIS?**

**Response :**

Thank you for your important question. KONIS data are reported through a secure web-based system managed by the Korea Disease Control and Prevention Agency. Participating hospitals submit monthly ICU patient-days and device-days, along with individual infection case reports based on standardized KONIS criteria. All data are anonymized, and no identifiable patient or hospital information is entered into the system. Data must be submitted within six weeks after each quarter ends.

**Q6. (Introduction) Is access to KONIS data free? How raw data can be accessed?**

**Response :**

Thank you for your insightful question. Access to KONIS data is restricted to protect patient and institutional confidentiality. Participating hospitals can view their own infection statistics and comparative summaries via the designated KONIS web system. However, access to the full raw data is limited to the KONIS principal investigator and is permitted only in accordance with the official procedures established by the Korean Society for Healthcare-associated Infection Control and Prevention (KOSHIC). Researchers may request data for academic purposes, but this requires a formal application under the KONIS Research Data Use Policy. Importantly, all data are anonymized, and hospital identities are strictly protected.

**Q7. (Introduction) "several studies using this information have been conducted [8]." if several it is expected to have more references.**

**Response :**

Thank you for your important question. We have revised it as follows.

**In line 73 of the revised manuscript,**

**Original:**

The KONIS data also contain information on causative pathogens and their antibiotic susceptibility, and several studies using this information have been conducted [8].

**Revised:**

The KONIS data also contain information on causative pathogens and their antibiotic susceptibility, and several studies using this information have been conducted [4, 8, 14].

**Q8. (Introduction) "have increased significantly" from which value to which values? in which period of time?**

**Response :**

Thank you for your important question. We have revised it as follows.

**In line 83 of the revised manuscript,**

**Original:**

The burden of MDR Gram–negative bacterial infections in South Korea have increased significantly, highlighting the growing importance of the KONIS system [11].

**Revised:**

The burden of MDR Gram–negative bacterial infections in South Korea has increased significantly, as evidenced by the rise in imipenem resistance of Pseudomonas aeruginosa from 13.9% to 30.8% and Acinetobacter baumannii from 7% to 73.5% between 1997 and 2016 [11].

**Q9. (Introduction) Briefly introduce the process for ICUs monitored in KONIS. Are all ICUs in the country?**

**Response :**

Thank you for your insightful question. KONIS is a nationwide surveillance system in which adult ICUs voluntarily participate. Each hospital selects one medical and one surgical ICU for monitoring, submitting standardized data on patient-days, device-days, and ICU-acquired infections (UTI, BSI, and pneumonia) via a web-based system. Data collection follows strict diagnostic and reporting guidelines defined in the KONIS Manual. Not all ICUs in Korea are included. Participation is voluntary and based on eligibility criteria, such as operating an infection control team.

**Q10. (Introduction) It is not sufficiently clear if such evaluations were already conducted or not.**

**Response :**

Thank you for the valuable comment. We have revised it as follows.

**In line 91 of the revised manuscript,**

**Original:**

Hence, this study aimed to visualize trends in infections and MDR gram-negative bacterial infections across individual ICUs using KONIS data. By applying a novel graph transformation method, we sought to condense complex surveillance results into interpretable patterns, enabling more intuitive comparisons and insights into ICU-specific resistance trends over time.

**Revised:**

Therefore, this study aimed to fill this gap by visualizing trends in infections and MDR gram-negative bacterial infections across individual ICUs using KONIS data. To our knowledge, no prior research has systematically evaluated or visualized antimicrobial resistance trends at the individual ICU level using KONIS data. By applying a novel graph transformation method, we sought to condense complex surveillance results into interpretable patterns, enabling more intuitive comparisons and insights into ICU-specific resistance trends over time.

**Q11. (Material and methods) Is KONIS data available from free to anyone? Please provide the source of data and describe how did you accessed it.**

**Response :**

Thank you for your valuable question. To ensure the confidentiality of patients and participating institutions, access to KONIS data is carefully controlled. Each hospital involved can access its own infection data and comparative reports through the dedicated KONIS web platform. However, full access to the raw dataset is restricted to the KONIS principal investigator and is granted only following the official protocols set by the Korean Society for Healthcare-associated Infection Control and Prevention (KOSHIC). Researchers interested in using the data for academic purposes must submit a formal request in compliance with the KONIS Research Data Use Policy. It is important to note that all data are anonymized, and the identities of the hospitals remain strictly confidential.

**Q12. (Material and methods) How the disease were selected?**

**Response :**

Thank you for your question. This study was conducted using KONIS data, and we selected infections monitored by KONIS—pneumonia, urinary tract infection, and bloodstream infection—as the subjects of our analysis.

**Q13. (Material and methods) "is generally low" please be specific.**

**Response :**

Thank you for your important question. We have revised it as follows.

**In line 111 of the revised manuscript,**

**Original:**

The diseases analyzed were pneumonia, urinary tract infection (UTI), and bloodstream infection (BSI). Because the risk of healthcare-associated infection is generally low in short-term inpatients, patients who had been hospitalized for less than 3 days were excluded [14].

**Revised:**

The diseases analyzed were pneumonia, urinary tract infection (UTI), and bloodstream infection (BSI). Because the risk of healthcare-associated infections among patients hospitalized for less than 3 days was reported to be below 1% in previous KONIS validation studies, these patients were excluded from the analysis [14].

**Q14. (Material and methods) Why 2022? (we are in 2025).**

**Response :**

Thank you for your valuable comment. The analysis of KONIS data for this study was initiated in 2023. While data spanning from 2006 to 2022 were accessible, the extensive volume posed practical limitations for a comprehensive analysis. To maintain analytical clarity and feasibility, we focused on two specific periods: the first five years and the most recent five years of the dataset.

**Q15. (Results) "Among these, ICUs in which infections (pneumonia, UTI, and BSI) caused by KP, PA, and AB occurred in at least one quarter were selected." This sentence belongs to methods.**

**Response :**

Thank you for your important question. We have revised it as follows.

**In line 223 of the revised manuscript,**

**Original:**

A total of 137 ICUs participated in the first 5 years (2006 to 2011) and 368 ICUs participated in the last 5 years (2017 to 2022). Among these, ICUs in which infections (pneumonia, UTI, and BSI) caused by KP, PA, and AB occurred in at least one quarter were selected.

**Revised:**

A total of 137 ICUs participated in the first 5 years (2006 to 2011) and 368 ICUs participated in the last 5 years (2017 to 2022). ~~Among these, ICUs in which infections (pneumonia, UTI, and BSI) caused by KP, PA, and AB occurred in at least one quarter were selected.~~

**Q16. (Results) Do not start a sentence with an abbreviation.**

**Response :**

Thank you for your valuable question. We have revised it as follows.

**In line 229 of the revised manuscript,**

**Original:**

ICUs where CS and CR bacterial infections occurred for more than two quarters were finally selected.

**Revised:**

Intensive care units (ICUs) where CS and CR bacterial infections occurred for more than two quarters were finally selected.

**Q17. (Results) Is the increased/decreased statistically significant?**

**Response :**

Thank you for your important question. We have revised it as follows.

**In line 246 of the revised manuscript,**

**Original:**

For KP infection, the proportion of ICUs in the red category increased in the last 5 years compared to the first 5 years, while the proportions in the yellow and green categories decreased (Fig. 5C and D). For PA infection, both the red and green category ICUs increased in proportion, while the yellow category decreased during the last 5 years compared to the first 5 years (Fig. 5E and F). For AB infection, the proportion of ICUs in the green category increased, whereas the red and yellow categories decreased in the last 5 years compared with the first 5 years (Fig. 5A and B).

**Revised:**

For KP infection, the proportion of ICUs in the red category increased in the last 5 years compared to the first 5 years (p-value 0.586), while the proportions in the yellow and green categories decreased (p-value 0.288, p-value 0.290) (Fig. 5C and D). For PA infection, both the red and green category ICUs increased in proportion (p-value 0.016, p-value 0.358), while the yellow category decreased during the last 5 years compared to the first 5 years (p-value 0.043) (Fig. 5E and F). For AB infection, the proportion of ICUs in the green category increased (p-value <0.001)., whereas the red and yellow categories decreased in the last 5 years compared with the first 5 years (p-value 0.649, p-value 0.187) (Fig. 5A and B).

**Q18. (Results) The graphical representations are blurred.**

**Response :**

We sincerely appreciate your valuable feedback. In accordance with your suggestion, we have adjusted the resolution of the graphical representations (Figures 1–5) to 600 DPI.

**Q19. (Discussion) Please also discuss the generalizability of the reported results as well as the utility for ICUs and/or country.**

**Response :**

Thank you for your valuable comments. If sufficient data were available, it might be possible to approach the issue in the same manner by varying the combinations of pathogens and antibiotics/antivirals/antifungals. And this could be done on a larger scale, such as at the national level, or on a smaller scale, such as at the hospital or even the ward level. However, selecting the appropriate reference values (e.g., cut-off length, scale-up factor) would likely be a crucial aspect of this approach. We addressed this issue in the previous revision by adding it to the discussion section.

**In line 317 of the revised manuscript,**

**Original:**

If sufficient data were available, it might be possible to approach the issue in the same manner by varying the combinations of pathogens and antibiotics/antivirals/antifungals. Also this could be done on a larger scale, such as at the national level, or on a smaller scale, such as at the hospital or even the ward level. However, selecting the appropriate reference values (e.g., cut-off length, scale-up factor) would likely be a crucial aspect of this approach.

**Q20. (Conclusions) "Existing data was transformed through a series of processes to visualize it in a single graph, making it easier to understand the situation across hundreds of ICUs." This is not a conclusion.**

**Response :**

Thank you for your important question. We have revised it as follows.

**In line 339 of the revised manuscript,**

**Original:**

Through the series of graph transformation processes presented in this study, it was possible to easily observe the trends of infections and resistant bacteria in hundreds of ICUs subjected to surveillance. Existing data was transformed through a series of processes to visualize it in a single graph, making it easier to understand the situation across hundreds of ICUs.

**Revised:**

Through the series of graph transformation processes presented in this study, it was possible to easily observe the trends of infections and resistant bacteria in hundreds of ICUs subjected to surveillance. ~~Existing data was transformed through a series of processes to visualize it in a single graph, making it easier to understand the situation across hundreds of ICUs.~~

**Q21. (Conclusions) "Data cannot be shared publicly because of potentially sensitive information" This declaration is opposite with "All participated intensive care unit’s data were fully anonymized before we accessed them".**

**Response :**

Thank you for your important comment. While all participating intensive care units’ data were fully anonymized before we accessed them to protect patient and institutional confidentiality, the raw KONIS dataset cannot be shared publicly due to the sensitive nature of the information. Access to the full raw data is strictly controlled and limited to the KONIS principal investigator, following official protocols established by the Korean Society for Healthcare-associated Infection Control and Prevention (KOSHIC). To address concerns regarding data accessibility while maintaining confidentiality, we have included a synthetic dataset.

We have revised our paper as described above and we believe we have addressed all questions and comment, but would be happy to provide further information or revision if necessary. Thank you for your consideration.

Sincerely yours,

Mi Suk Lee, MD, PhD

Professor,

Division of Infectious Diseases,

Department of Internal Medicine,

Kyung Hee University College of Medicine,

Kyung Hee University Hospital,

23, Kyung Hee Dae-ro, Dongdaemungu, Seoul, 02447,

Republic of Korea,

Phone: (82) 2 9588159, Fax number: (82) 2 9681848,

E-mail: mslee7@gmail.com
