# Supplementary material for: Visualization of multidrug-resistant bacterial infection trends in the intensive care units
Source: PLoS One. 2025 Aug 28;20(8):e0330765. doi: 10.1371/journal.pone.0330765 (PMC12393710; doi:10.1371/journal.pone.0330765)
Supplement: Attachment — Submitted filename: Response_to_Reviewers_auresp_4.docx [file pone.0330765.s008.docx]

31 July 2025

Ali Amanati

Academic Editor

PLOS ONE

Re: PONE-D-24-03240R3,

“Visualization of multidrug-resistant bacterial infection trends in the intensive care units”

**Dr. Ali Amanati:**

On behalf of the authors, I would like to thank you and the reviewers for helpful comments on our submitted manuscript, “Visualization of multidrug-resistant bacterial infection trends in the intensive care units”. We have carefully reviewed the comments and have revised the manuscript accordingly.

**Reviewer #8’s comments:**

**Q1. (Abstract) It is not sufficiently clear why you choose the two periods or bacteria.**

**Response :**

Thank you for your thoughtful feedback. The selection of these two periods is the result of considering changes in the diagnostic criteria for healthcare-associated infections in intensive care units and changes in the size of beds in participating medical institutions. Among the diagnostic criteria, the criteria for urinary tract infection were partially changed in 2012 and 2016, and the two periods 2006-2011 and 2017-2022, which have remained relatively the same due to the addition of non-cultured diagnostic laboratory tests to the diagnostic criteria for bloodstream infection and pneumonia since 2016, and the rapid increase in the number of participating institutions and intensive care units since 2016, were compared.

The three strains of Acinetobacter baumannii, Klebsiella pneumoniae, and Pseudomonas aeruginosa were selected because they are the main causative agents that have been continuously increasing in the incidence and antibiotic resistance rate of healthcare-associated infections in intensive care units in Korea since 2017, while Staphylococcus aureus and Enterococci are relatively reduced or similar in incidence, so they were excluded from the comparison.

**In line 112 of the revised manuscript,**

**Revised:**

**Setting and Study Design**

This study was based on surveillance data from the Korean National Healthcare–associated Infections Surveillance System (KONIS), which monitors healthcare-associated infections (HAIs) in intensive care units (ICUs) nationwide. Participation in KONIS is voluntary and limited to medical institutions operating ICUs with ≥100 inpatient beds. As of 2022, 357 ICUs from 274 hospitals participated, representing approximately 85.9% of eligible institutions in Korea.

KONIS data are reported through the Korea Disease Control and Prevention Agency’s (KDCA) Integrated Disease and Health Management System, a secure internet-based platform. Each institution designates authorized personnel who register, authenticate with digital certificates, and submit HAI data directly to the KONIS system. While basic statistical summaries are accessible to all registered users, raw data use for academic purposes requires approval by the KONIS Steering Committee. This study was approved for use of raw data, which were provided in de-identified Excel format.

We selected two time periods for analysis: 2006–2011 and 2017–2022. These were chosen based on changes in KONIS diagnostic criteria and expansion of participating institutions. In particular, diagnostic standards for urinary tract infections were revised in 2012 and 2016, and laboratory-based diagnostics for bloodstream infections and pneumonia were introduced in 2016. Additionally, the number of participating ICUs grew rapidly after 2016, enabling more stable data comparison across ICUs in the selected periods.

Among all HAIs monitored in KONIS, we focused on urinary tract infection, bloodstream infection, and pneumonia, which are commonly associated with invasive device use. The study analyzed *Klebsiella pneumoniae*, *Pseudomonas aeruginosa,* and *Acinetobacter baumannii*, which have shown rising incidence and resistance rates in Korean ICUs in recent years. In contrast, *Staphylococcus aureus* and *Enterococcus spp*., which have shown declining or stable trends, were excluded from this analysis.

Although the present year is 2025, we utilized KONIS data from 2022 because annual datasets are finalized and publicly reported 12 to 18 months after data collection ends. The study was planned in 2023 based on the most recently available and complete dataset at that time.

**Q4. (Introduction) It is unclear why the reported prevalence is for US but the study is done on Korean data.**

**Response :**

Thank you for your insightful comment. The content has been changed to the following data based on the Korean intensive care unit.

**In line 62 of the revised manuscript,**

**Original:**

Worldwide, nosocomial and multi-drug resistant bacterial infections have increased significantly. For example, carbapenem-resistant *Acinetobacter baumannii* (CRAB) accounted for over 50% of carbapenem-resistant gram-negative bacilli in intensive care units (ICUs) [1], carbapenem-resistant *Enterobacterales* (CRE) prevalence in the U.S. rose from 1.2% in 2001 to 4.2% in 2011 [2], and reports of carbapenemase-producing P. aeruginosa have increased globally since 2010 [3].

**Revised:**

~~Worldwide,~~ Nosocomial and multi-drug resistant bacterial infections have increased significantly. As of 2022, *Acinetobacter baumannii* accounts for more than 50% of carbapenem-resistant gram-negative bacilli isolated in intensive care units in Korea, followed by *Klebsiella pneumoniae* and *Pseudomonas aeruginosa* accounting for more than 20% and 18%, respectively [1].

Reference [1] Korean Disease Control and Prevention Agency. Annual report of the Korean national healthcare-associated infections surveillance system (KONIS) 2022.

**Q5. (Introduction) How data are reported to KONIS?**

**Response :**

Thank you for your important question. KONIS data is registered and stored in the Integrated Disease and Health Management System, an Internet-based computer program with servers at the Korea Disease Control and Prevention Agency. The person in charge of the participating institution will log in with the certificate after registering as a user and registering a certified certificate, select KONIS from the system, and enter the infected patient data of the participating institution.

**In line 105 of the revised manuscript,**

**Revised:**

**Setting and Study Design**

This study was based on surveillance data from the Korean National Healthcare–associated Infections Surveillance System (KONIS), which monitors healthcare-associated infections (HAIs) in intensive care units (ICUs) nationwide. Participation in KONIS is voluntary and limited to medical institutions operating ICUs with ≥100 inpatient beds. As of 2022, 357 ICUs from 274 hospitals participated, representing approximately 85.9% of eligible institutions in Korea.

KONIS data are reported through the Korea Disease Control and Prevention Agency’s (KDCA) Integrated Disease and Health Management System, a secure internet-based platform. Each institution designates authorized personnel who register, authenticate with digital certificates, and submit HAI data directly to the KONIS system. While basic statistical summaries are accessible to all registered users, raw data use for academic purposes requires approval by the KONIS Steering Committee. This study was approved for use of raw data, which were provided in de-identified Excel format.

We selected two time periods for analysis: 2006–2011 and 2017–2022. These were chosen based on changes in KONIS diagnostic criteria and expansion of participating institutions. In particular, diagnostic standards for urinary tract infections were revised in 2012 and 2016, and laboratory-based diagnostics for bloodstream infections and pneumonia were introduced in 2016. Additionally, the number of participating ICUs grew rapidly after 2016, enabling more stable data comparison across ICUs in the selected periods.

Among all HAIs monitored in KONIS, we focused on urinary tract infection, bloodstream infection, and pneumonia, which are commonly associated with invasive device use. The study analyzed *Klebsiella pneumoniae*, *Pseudomonas aeruginosa,* and *Acinetobacter baumannii*, which have shown rising incidence and resistance rates in Korean ICUs in recent years. In contrast, *Staphylococcus aureus* and *Enterococcus spp*., which have shown declining or stable trends, were excluded from this analysis.

Although the present year is 2025, we utilized KONIS data from 2022 because annual datasets are finalized and publicly reported 12 to 18 months after data collection ends. The study was planned in 2023 based on the most recently available and complete dataset at that time.

**Q6. (Introduction) Is access to KONIS data free? How raw data can be accessed?**

**Response :**

Thank you for your insightful question. The person in charge of the participating organization must apply for access to KONIS system when logging in to the Korea Disease Control and Prevention Agency's Integrated Disease Management System for the first time, and after the permission is approved by the project management office, the data can be entered at the participating organization. All analytical statistical data that is not a security issue can be accessed by any registered user, and all data from the organization can be downloaded as an Excel file.
KONIS data from all institutions that have been disclosed can be checked through ‘<https://konis.cafe24.com/xe/>’ or report papers, etc. The general manager of KONIS has the right to view all original raw data registered in the KONIS system, and when viewing or using raw data for academic research purposes, they can only be viewed and used in accordance with certain procedures set by the Steering Committee.

**In line 108 of the revised manuscript,**

**Revised:**

**Setting and Study Design**

This study was based on surveillance data from the Korean National Healthcare–associated Infections Surveillance System (KONIS), which monitors healthcare-associated infections (HAIs) in intensive care units (ICUs) nationwide. Participation in KONIS is voluntary and limited to medical institutions operating ICUs with ≥100 inpatient beds. As of 2022, 357 ICUs from 274 hospitals participated, representing approximately 85.9% of eligible institutions in Korea.

KONIS data are reported through the Korea Disease Control and Prevention Agency’s (KDCA) Integrated Disease and Health Management System, a secure internet-based platform. Each institution designates authorized personnel who register, authenticate with digital certificates, and submit HAI data directly to the KONIS system. While basic statistical summaries are accessible to all registered users, raw data use for academic purposes requires approval by the KONIS Steering Committee. This study was approved for use of raw data, which were provided in de-identified Excel format.

We selected two time periods for analysis: 2006–2011 and 2017–2022. These were chosen based on changes in KONIS diagnostic criteria and expansion of participating institutions. In particular, diagnostic standards for urinary tract infections were revised in 2012 and 2016, and laboratory-based diagnostics for bloodstream infections and pneumonia were introduced in 2016. Additionally, the number of participating ICUs grew rapidly after 2016, enabling more stable data comparison across ICUs in the selected periods.

Among all HAIs monitored in KONIS, we focused on urinary tract infection, bloodstream infection, and pneumonia, which are commonly associated with invasive device use. The study analyzed *Klebsiella pneumoniae*, *Pseudomonas aeruginosa,* and *Acinetobacter baumannii*, which have shown rising incidence and resistance rates in Korean ICUs in recent years. In contrast, *Staphylococcus aureus* and *Enterococcus spp*., which have shown declining or stable trends, were excluded from this analysis.

Although the present year is 2025, we utilized KONIS data from 2022 because annual datasets are finalized and publicly reported 12 to 18 months after data collection ends. The study was planned in 2023 based on the most recently available and complete dataset at that time.

**Q9. (Introduction) Briefly introduce the process for ICUs monitored in KONIS. Are all ICUs in the country?**

**Response :**

Thank you for the valuable comment. KONIS ICU surveillance is carried out every year by receiving voluntary applications from medical institutions that operate intensive care units with a certain number of beds or more (participation of 1-2 intensive care units depending on the size). As of 2022, 357 ICUs of the 274 medical institutions participated with more than 100 inpatient beds, and 85.9% of the eligible medical institutions participated.

**In line 99 of the revised manuscript,**

**Revised:**

**Setting and Study Design**

This study was based on surveillance data from the Korean National Healthcare–associated Infections Surveillance System (KONIS), which monitors healthcare-associated infections (HAIs) in intensive care units (ICUs) nationwide. Participation in KONIS is voluntary and limited to medical institutions operating ICUs with ≥100 inpatient beds. As of 2022, 357 ICUs from 274 hospitals participated, representing approximately 85.9% of eligible institutions in Korea.

KONIS data are reported through the Korea Disease Control and Prevention Agency’s (KDCA) Integrated Disease and Health Management System, a secure internet-based platform. Each institution designates authorized personnel who register, authenticate with digital certificates, and submit HAI data directly to the KONIS system. While basic statistical summaries are accessible to all registered users, raw data use for academic purposes requires approval by the KONIS Steering Committee. This study was approved for use of raw data, which were provided in de-identified Excel format.

We selected two time periods for analysis: 2006–2011 and 2017–2022. These were chosen based on changes in KONIS diagnostic criteria and expansion of participating institutions. In particular, diagnostic standards for urinary tract infections were revised in 2012 and 2016, and laboratory-based diagnostics for bloodstream infections and pneumonia were introduced in 2016. Additionally, the number of participating ICUs grew rapidly after 2016, enabling more stable data comparison across ICUs in the selected periods.

Among all HAIs monitored in KONIS, we focused on urinary tract infection, bloodstream infection, and pneumonia, which are commonly associated with invasive device use. The study analyzed *Klebsiella pneumoniae*, *Pseudomonas aeruginosa,* and *Acinetobacter baumannii*, which have shown rising incidence and resistance rates in Korean ICUs in recent years. In contrast, *Staphylococcus aureus* and *Enterococcus spp*., which have shown declining or stable trends, were excluded from this analysis.

Although the present year is 2025, we utilized KONIS data from 2022 because annual datasets are finalized and publicly reported 12 to 18 months after data collection ends. The study was planned in 2023 based on the most recently available and complete dataset at that time.

**Q11. (Material and methods) Is KONIS data available from free to anyone? Please provide the source of data and describe how did you accessed it.**

**Response :**

Thank you for your valuable question. All analytical statistical data that is not a security problem can be accessed by any registered user, and all data from the organization can be downloaded as an Excel file. The KONIS materials of all participating institutions that have been disclosed can be checked through ‘<https://konis.cafe24.com/xe/>’or research papers, etc., and can be used free of charge, and the use of the original raw data requires application and approval in accordance with the KONIS Operating Regulations. The KONIS raw data used in this study were approved for use by the KONIS Steering Committee in accordance with the relevant regulations, and the relevant materials were provided as Excel files.

**In line 105 of the revised manuscript,**

**Revised:**

**Setting and Study Design**

This study was based on surveillance data from the Korean National Healthcare–associated Infections Surveillance System (KONIS), which monitors healthcare-associated infections (HAIs) in intensive care units (ICUs) nationwide. Participation in KONIS is voluntary and limited to medical institutions operating ICUs with ≥100 inpatient beds. As of 2022, 357 ICUs from 274 hospitals participated, representing approximately 85.9% of eligible institutions in Korea.

KONIS data are reported through the Korea Disease Control and Prevention Agency’s (KDCA) Integrated Disease and Health Management System, a secure internet-based platform. Each institution designates authorized personnel who register, authenticate with digital certificates, and submit HAI data directly to the KONIS system. While basic statistical summaries are accessible to all registered users, raw data use for academic purposes requires approval by the KONIS Steering Committee. This study was approved for use of raw data, which were provided in de-identified Excel format.

We selected two time periods for analysis: 2006–2011 and 2017–2022. These were chosen based on changes in KONIS diagnostic criteria and expansion of participating institutions. In particular, diagnostic standards for urinary tract infections were revised in 2012 and 2016, and laboratory-based diagnostics for bloodstream infections and pneumonia were introduced in 2016. Additionally, the number of participating ICUs grew rapidly after 2016, enabling more stable data comparison across ICUs in the selected periods.

Among all HAIs monitored in KONIS, we focused on urinary tract infection, bloodstream infection, and pneumonia, which are commonly associated with invasive device use. The study analyzed *Klebsiella pneumoniae*, *Pseudomonas aeruginosa,* and *Acinetobacter baumannii*, which have shown rising incidence and resistance rates in Korean ICUs in recent years. In contrast, *Staphylococcus aureus* and *Enterococcus spp*., which have shown declining or stable trends, were excluded from this analysis.

Although the present year is 2025, we utilized KONIS data from 2022 because annual datasets are finalized and publicly reported 12 to 18 months after data collection ends. The study was planned in 2023 based on the most recently available and complete dataset at that time

**Q12. (Material and methods) How the disease were selected?**

**Response :**

Thank you for your important question. Since 2006, the KONIS Intensive Care Unit Surveillance System has been operating by selecting urinary tract infections, bloodstream infections, and pneumonia, which are common infections related to the use of invasive instruments, as targets for surveillance of healthcare-associated infections in intensive care units. The strains of Acinetobacter baumannii, Klebsiella pneumoniae, and Pseudomonas aeruginosa, whose frequency and antibiotic resistance rates are increasing, were selected as target strains.

**In line 118 of the revised manuscript,**

**Revised:**

**Setting and Study Design**

This study was based on surveillance data from the Korean National Healthcare–associated Infections Surveillance System (KONIS), which monitors healthcare-associated infections (HAIs) in intensive care units (ICUs) nationwide. Participation in KONIS is voluntary and limited to medical institutions operating ICUs with ≥100 inpatient beds. As of 2022, 357 ICUs from 274 hospitals participated, representing approximately 85.9% of eligible institutions in Korea.

KONIS data are reported through the Korea Disease Control and Prevention Agency’s (KDCA) Integrated Disease and Health Management System, a secure internet-based platform. Each institution designates authorized personnel who register, authenticate with digital certificates, and submit HAI data directly to the KONIS system. While basic statistical summaries are accessible to all registered users, raw data use for academic purposes requires approval by the KONIS Steering Committee. This study was approved for use of raw data, which were provided in de-identified Excel format.

We selected two time periods for analysis: 2006–2011 and 2017–2022. These were chosen based on changes in KONIS diagnostic criteria and expansion of participating institutions. In particular, diagnostic standards for urinary tract infections were revised in 2012 and 2016, and laboratory-based diagnostics for bloodstream infections and pneumonia were introduced in 2016. Additionally, the number of participating ICUs grew rapidly after 2016, enabling more stable data comparison across ICUs in the selected periods.

Among all HAIs monitored in KONIS, we focused on urinary tract infection, bloodstream infection, and pneumonia, which are commonly associated with invasive device use. The study analyzed *Klebsiella pneumoniae*, *Pseudomonas aeruginosa,* and *Acinetobacter baumannii*, which have shown rising incidence and resistance rates in Korean ICUs in recent years. In contrast, *Staphylococcus aureus* and *Enterococcus spp*., which have shown declining or stable trends, were excluded from this analysis.

Although the present year is 2025, we utilized KONIS data from 2022 because annual datasets are finalized and publicly reported 12 to 18 months after data collection ends. The study was planned in 2023 based on the most recently available and complete dataset at that time

**Q14. (Material and methods) Why 2022? (we are in 2025).**

**Response :**

Thank you for your valuable comment. KONIS data was collects, refines, and analyzes from July of the current year to June of the following year, and there is a certain time delay in reporting the results. For reference, the final results for 2022 are reported in the second half of 2023 or early 2024. Please consider that the plan for this study was made in 2023 and is the result of compiling data from 2022.

**In line 124 of the revised manuscript,**

**Revised:**

**Setting and Study Design**

This study was based on surveillance data from the Korean National Healthcare–associated Infections Surveillance System (KONIS), which monitors healthcare-associated infections (HAIs) in intensive care units (ICUs) nationwide. Participation in KONIS is voluntary and limited to medical institutions operating ICUs with ≥100 inpatient beds. As of 2022, 357 ICUs from 274 hospitals participated, representing approximately 85.9% of eligible institutions in Korea.

KONIS data are reported through the Korea Disease Control and Prevention Agency’s (KDCA) Integrated Disease and Health Management System, a secure internet-based platform. Each institution designates authorized personnel who register, authenticate with digital certificates, and submit HAI data directly to the KONIS system. While basic statistical summaries are accessible to all registered users, raw data use for academic purposes requires approval by the KONIS Steering Committee. This study was approved for use of raw data, which were provided in de-identified Excel format.

We selected two time periods for analysis: 2006–2011 and 2017–2022. These were chosen based on changes in KONIS diagnostic criteria and expansion of participating institutions. In particular, diagnostic standards for urinary tract infections were revised in 2012 and 2016, and laboratory-based diagnostics for bloodstream infections and pneumonia were introduced in 2016. Additionally, the number of participating ICUs grew rapidly after 2016, enabling more stable data comparison across ICUs in the selected periods.

Among all HAIs monitored in KONIS, we focused on urinary tract infection, bloodstream infection, and pneumonia, which are commonly associated with invasive device use. The study analyzed *Klebsiella pneumoniae*, *Pseudomonas aeruginosa,* and *Acinetobacter baumannii*, which have shown rising incidence and resistance rates in Korean ICUs in recent years. In contrast, *Staphylococcus aureus* and *Enterococcus spp*., which have shown declining or stable trends, were excluded from this analysis.

Although the present year is 2025, we utilized KONIS data from 2022 because annual datasets are finalized and publicly reported 12 to 18 months after data collection ends. The study was planned in 2023 based on the most recently available and complete dataset at that time

We have revised our paper as described above and we believe we have addressed all questions and comment, but would be happy to provide further information or revision if necessary. Thank you for your revisions and your continued efforts to address the reviewer’s comments.

Sincerely yours,

Mi Suk Lee, MD, PhD

Professor,

Division of Infectious Diseases,

Department of Internal Medicine,

Kyung Hee University College of Medicine,

Kyung Hee University Hospital,

23, Kyung Hee Dae-ro, Dongdaemungu, Seoul, 02447,

Republic of Korea,

Phone: (82) 2 9588159, Fax number: (82) 2 9681848,

E-mail: mslee7@gmail.com
